# Effects of a Volatile Organic Compound Filter on Breath Profiles Measured by Secondary Electrospray High-Resolution Mass Spectrometry

**DOI:** 10.3390/molecules28010045

**Published:** 2022-12-21

**Authors:** Ronja Weber, Jérôme Kaeslin, Sophia Moeller, Nathan Perkins, Srdjan Micic, Alexander Moeller

**Affiliations:** 1 Department of Respiratory Medicine and Childhood Research Center, University Children’s Hospital Zurich, Steinwiesstrasse 75, 8032 Zurich, Switzerland; 2 Department of Chemistry and Applied Biosciences, Swiss Federal Institute of Technology, Vladimir-Prelog Weg 1-5/10, 8093 Zurich, Switzerland; 3 Division of Clinical Chemistry and Biochemistry, University Children’s Hospital Zurich, Steinwiesstrasse 75, 8032 Zurich, Switzerland; 4 Faculty of Medicine, University of Zurich, Raemistrasse 71, 8006 Zurich, Switzerland

**Keywords:** filter, SESI-HRMS, volatile organic compounds, contaminants, metabolites, ambient air

## Abstract

Environmental volatile organic compounds (VOCs) from the ambient air potentially influence on-line breath analysis measurements by secondary electrospray ionization high-resolution mass spectrometry (SESI-HRMS). The aim of this study was to investigate how inhaling through a VOC filter affects the detected breath profiles and whether it is feasible to integrate such filters into routine measurements. A total of 24 adult participants performed paired breath analysis measurements with and without the use of an activated carbon filter for inspiration. Concordance correlation coefficients (CCCs) and the Bland–Altman analysis were used to assess the agreement between the two methods. Additionally, the effect on a selection of known metabolites and contaminants was analyzed. Out of all the detected features, 78.3% showed at least a moderate agreement before and after filter usage (CCC > 0.9). The decrease in agreement of the remaining *m*/*z* features was mostly associated with reduced signal intensities after filter usage. Although a moderate-to-substantial concordance was found for almost 80% of the *m*/*z* features, the filter still had an effect by decreasing signal intensities, not only for contaminants, but also for some of the studied metabolites. Operationally, the use of the filter complicated and slowed down the conductance of measurements, limiting its applicability in clinical studies.

## 1. Introduction

Breath contains a variety of volatile organic compounds (VOCs), which can be of endogenous and environmental origin. The main goal of breath analysis is to identify endogenous VOCs that are linked to the human metabolism or such disease-associated processes as, for example, inflammation [1]. The detection of such biologically relevant exhaled compounds has the potential to revolutionize the diagnosis and monitoring of respiratory diseases. However, breath also contains a variety of environmental compounds that influence the measurements [2]. The composition of the ambient air in the room where the sample is taken might have a non-trivial impact on the measurements. Although this has been hypothesized, no study has assessed this systematically for on-line breath sampling by SESI-HRMS so far. While the strength of the SESI-HRMS methodology lies in the detection of heavier molecules with mass-to-charge (*m*/*z*) ratios up to 500 [3], airborne plasticizers with significant intensities are also detected in the upper range.

Using VOC-filtered air on inspiration reduces the number of ambient contaminants. There are generally two methods for taking samples with filtered ambient air, either using a VOC filter for inspiration [4,5,6,7,8] or using a reservoir of previously filtered or synthetic air [9,10,11]. Using an inspiratory filter complicates the conductance of measurements by adding a period of tidal breathing before the actual breath sampling. Most studies used a five-minute period of tidal breathing through the filter, which is based on references from inert gas wash-out [5]. However, it was demonstrated that the duration of pre-breathing until significant wash-out is reached depends on the properties of the individual molecule and can be 20 to 30 min or more [4,10,12]. A recent study even argues that using a reservoir of clean air adds additional unpredictable complexity to breath sampling [11]. According to our literature research, only a few pediatric studies did use a VOC filter or a purified air reservoir for inspiration. One recent study that investigated children with cystic fibrosis and Pseudomonas aeruginosa included five minutes of tidal breathing through a VOC filter before the breath sampling [6]. Another recent study used a filtered, clean air supply for measurements in children [9]. A reason for this might be the limited applicability in younger children as they are not able to properly seal the mouthpiece or mask for several minutes of pre-breathing.

Many previously published clinical studies with SESI-HRMS neither used a VOC filter for inspiration nor collected ambient air samples [13,14,15,16], and no conclusions about the potential role of airborne contaminants for this untargeted methodology without pre-separation could be drawn so far. Therefore, we wanted to investigate the inclusion of a filter as a methodological change in conducting breath analysis studies with this specific methodology. One aim of this study was to find out whether it is feasible for participants and operators to use a VOC filter for clinical breath analysis measurements by SESI-HRMS. We also aimed to investigate whether using a VOC filter reduces contaminants from the ambient air and how it affects the intensities of known human breath metabolites. We hypothesized that using a filter for inspiration would decrease the intensity of compounds, including contaminants and plasticizers but potentially also certain endogenous compounds that might also be present in the ambient air of our laboratory.

## 2. Results

**Figure 1:**
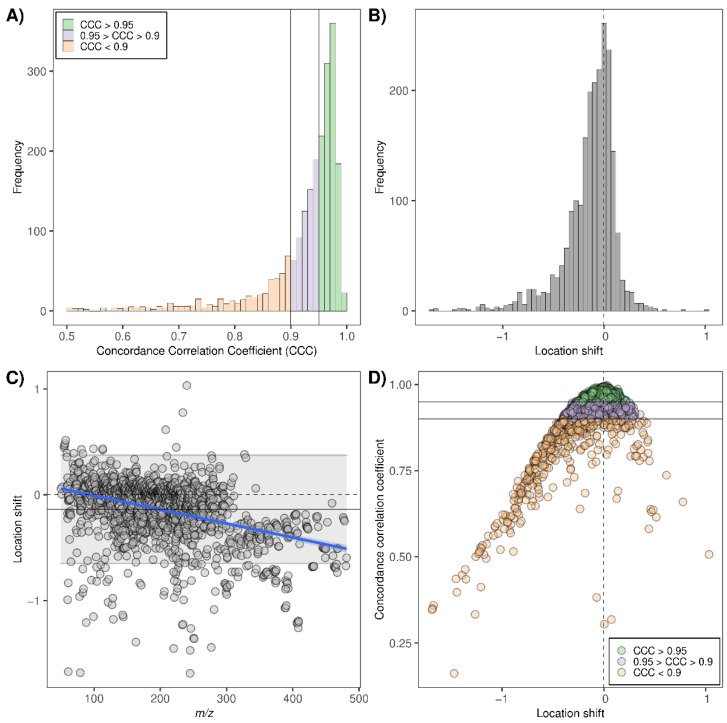
(**A**) Histogram of the CCCs calculated for all exhaled breath features. For visualization purposes and due to their lower frequency, CCC values below 0.5 were excluded from the plot. Two vertical lines represent CCC cut-offs 0.95 and 0.9. Green: CCC > 0.95; purple: 0.95 > CCC > 0.9; orange CCC < 0.9. (**B**) Histogram of the location shifts of all exhaled breath features. Vertical dashed line: location shift = 0. (**C**) Scatter plot of location shifts as a function of *m*/*z*. Horizontal dashed line: location shift = 0. Gray ribbon: 95% confidence band. Gray horizontal line: average location shift over all features. Blue line: simple linear regression capturing the trend of the location shift as a function of *m*/*z*. (**D**) Scatter plot of CCC as a function of the location shift for all exhaled features. Coloring and horizontal lines indicate CCC ranges as in plot (**A**). Vertical dashed line: location shift = 0.

A total number of 2193 *m*/*z* features resulting from exhaled breath were detected across all samples. Figure 1A shows the distribution of concordance correlation coefficients (CCCs) of the detected *m*/*z* features. When applying a more stringent criterion for agreement from [17], 1097 (50%) of the features had a CCC > 0.95, exhibiting a substantial to almost perfect agreement. In the moderate agreement range (0.9 < CCC < 0.95), 621 features were found, resulting in a total of 1717 features (78.3%) with a CCC > 0.9. The distribution of the location shift (Figure 1B) shows a left-skewed form (away from location shift 0) indicating a signal intensity reduction after applying the filter, which is reflected in a lower agreement for some of the exhaled breath features (Figure 1D). Furthermore, when investigating the location shift as a function of *m*/*z* (Figure 1C), a trend for signal reduction after filter application was observed towards heavier species.

### 2.1. Assessment of Exhaled Metabolites after Filter Usage

**Figure 2:**
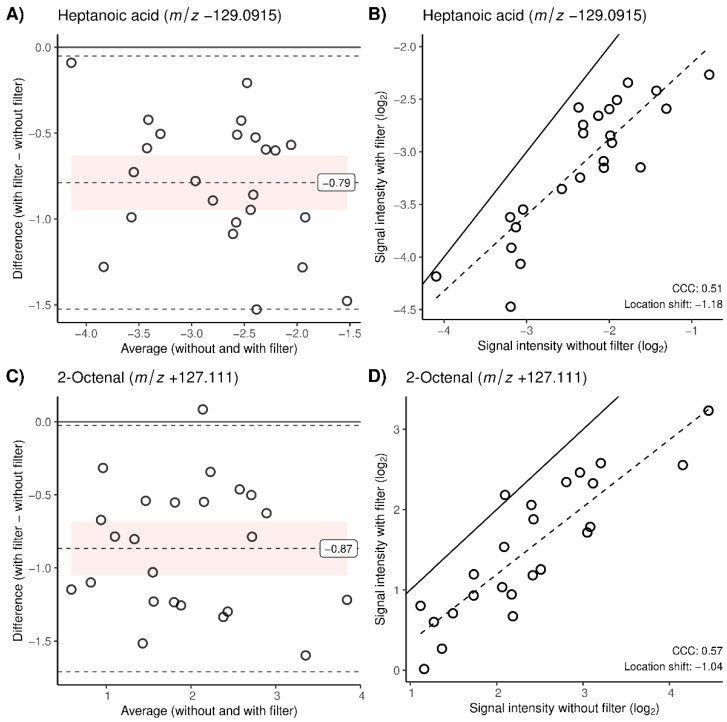
(**A**,**B**) Heptanoic acid (*m*/*z* −129.091) in negative ionization mode, (**C**,**D**) 2-octenal (*m*/*z* +127.111) in positive ionization mode. (**A**,**C**): Bland–Altman plots. Horizontal dashed line in the pink area represents the bias (average of with filter to without filter differences) with its value as a label on the right. A negative value of the bias indicates signal intensity reduction with filter usage. Pink ribbon: 95% confidence interval of the bias. Top and bottom dotted lines: agreement limits, i.e., ± 1.96s, s = standard deviation of the differences. Horizontal full line (black): line of zero difference. (**B**,**D**): signal intensities without filter usage vs. signal intensities with filter usage for all observed pairs. Diagonal full line (black): the line of perfect agreement. Dotted line: simple linear regression for intensity values with filter usage as a function of intensity values without filter usage. Included in the bottom right corner are concordance correlation coefficient (CCC) and the location shift.

A total of 69 metabolites, previously reported in human breath, could be found in the exhaled breath data of this study (mass error Δ*m*/*z* < 0.001 (10 ppm) to the reported *m*/*z* values in the literature). A total of 44 of the 69 found metabolites were also found in the ambient air samples, and 36 of them had a CCC > 0.95 and another 22 had a CCC between 0.9 and 0.95, which indicates that the majority of metabolites were in acceptable concordance with the measurements without a filter. Table 1 lists the 11 exhaled compounds that had lower agreement with a CCC < 0.9. They include fatty acids in negative ionization mode as well as aldehydes and amino acids in positive ionization mode. All compounds except for the water trimer and glycine had a clearly negative location shift and bias in the Bland–Altman plots (Figure S1), and therefore a lower intensity with filter usage. Most of them were also detected in ambient air, indicating that they were reduced by the filter. Figure 2 includes Bland–Altman and CCC plots of the paired signal intensities for the metabolites with the weakest concordance in each ionization mode: heptanoic acid for negative (Figure 2A,B) and 2-octenal (Figure 2C,D) for positive mode. The clear reduction is confirmed by the large biases and location shifts visualized in Figure 2 Interestingly, not all of the metabolites with a decreased abundance after filter application could be found in the ambient air samples. The two fatty acids, ω-oxoundecanoic acid and ω-hydroxyoctanoic acid, had a visibly negative location shift and bias (see Figure S1) and were not detected in the room air at all. Glycine was also not found in the ambient air samples but had a positive bias and location shift (see Figure S1). The water trimer had an increased intensity. It represents humidity and was therefore also found in the humidified air. Among the 58 metabolites with a moderate-to-substantial agreement (CCC > 0.9), 36 were found in ambient air samples. Figure S1 in the Supplementary Material includes Bland–Altman and CCC plots of the paired signal intensities without vs. with a filter for all the detected metabolites regardless of their CCC. Additionally, Table S1 lists all matched metabolites and whether they were found in ambient air.

### 2.2. Effect of the Filter on Contaminants

**Figure 3:**
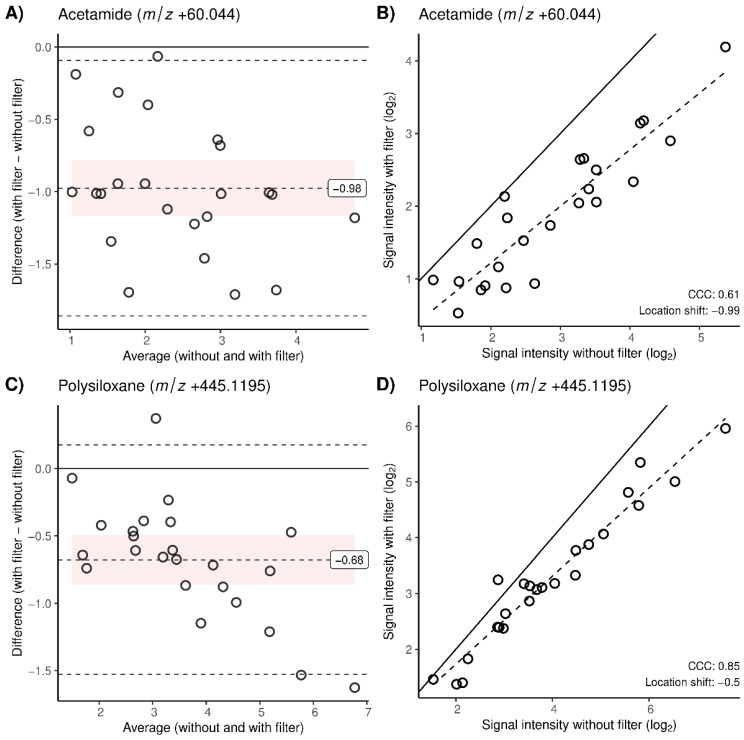
Same as in Figure 2, Bland–Altman and signal intensities of all pairs of measurements are presented for (**A**,**B**) acetamide (*m*/*z* +60.044) and (**C**,**D**) polysiloxane (*m*/*z* +445.1195), both contaminants in positive ionization mode.

A set of 23 common mass spectrometry contaminants could be detected (mass error Δ*m*/*z* < 0.001, i.e., < 10 ppm). In total, 16/23 were detected in ambient air, and 10 of the contaminants had a CCC < 0.9 and therefore a discrepancy before and after the filter usage. All of them were positively ionized and all except for one had a negative location shift and a decreased intensity after inspiration through the filter. Table 2 includes detailed information about the contaminants with a CCC < 0.9. Six of them were polysiloxanes, which are known to be present at high intensities in the air of our laboratory. Additionally, three solvents, methanol, acetamide and dimethyl formamide, were also found to be present in ambient air and to have reduced signal intensities as well as clearly negative location shifts and biases. Figure 3 shows Bland–Altman plots and plots of the paired signal intensities without vs. with a filter for acetamide (Figure 3A,B) and a polysiloxane (Figure 3C,D), visualizing their clear reduction. A fragment of phthalate esters was the only contaminant with a CCC < 0.9 that was not found in ambient air. However, its lower CCC was more related to two single observations with a stronger disagreement, which we could not explain. From the 13 contaminants with a CCC > 0.9, 7 were also found in ambient air. Bland–Altman plots of all the matched contaminants can be found in the Supplementary Figure S2 and the detailed information about them is listed in Table S2.

### 2.3. Effects on VOCs in Ambient Air

**Figure 4:**
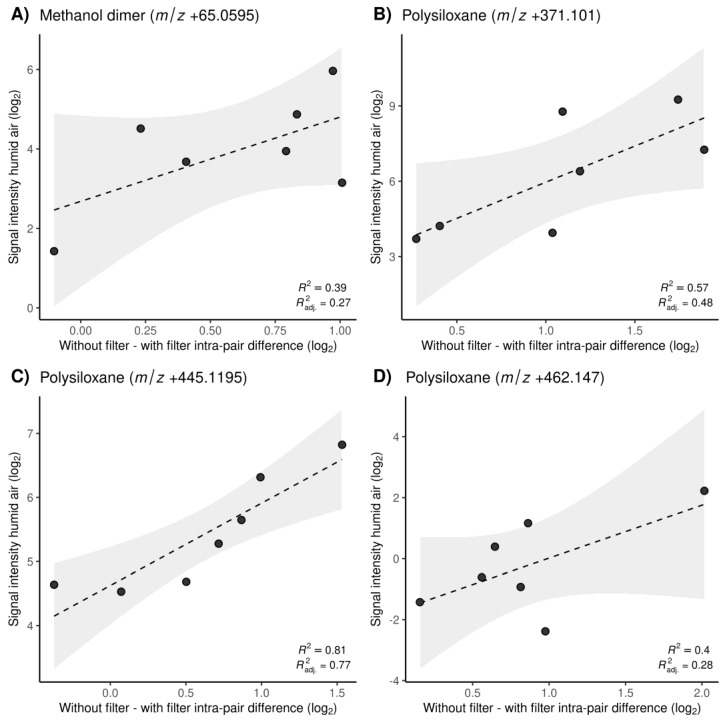
Plot of the *m*/*z* feature’s signal intensities in humidified ambient air samples on the y-axis vs. intra-pair signal intensity differences (without filter; with filter) on the x-axis. The paired observations were taken in the closest possible time proximity to the measured humidified air samples. The dashed line represents the regression line with a gray ribbon enclosed by the 95% confidence interval limits. (**A**) Methanol dimer (*m*/*z* 65.0595), (**B**–**D**) polysiloxanes (*m*/*z* 371.101, 445.1195, 462.147).

Lastly, the targeted metabolites and contaminants with a CCC < 0.9 and a negative location shift were put into a relationship with their respective intensities in matched ambient air samples. For this analysis, we only considered breath samples that were recorded immediately after the collection of ambient air samples. For the contaminants with a CCC < 0.9, there was a trend showing that higher intensities in ambient air resulted in larger intra-pair differences with and without the filter. This indicates that the filtering effect increases with higher concentrations of these compounds. On the x-axis of Figure 4, the within-subject difference of intensities before and after filter usage is shown for methanol dimer (Figure 4A) as well as for multiple polysiloxanes (Figure 4B–D). The y-axis shows the intensities of these features in ambient air. The trend was not visible for the metabolites that had a CCC < 0.9 and were present in ambient air. The corresponding plots can be found in Figure S3.

## 3. Discussion

**Figure 5:**
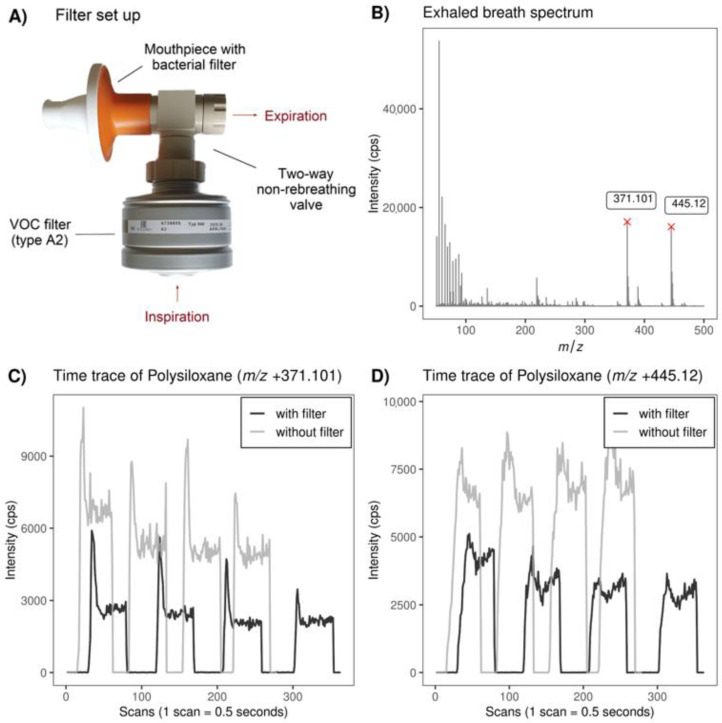
(**A**) Picture of the VOC filter connected to two-way non-rebreathing T-valve, which is custom-made from PEEK and has an attached mouthpiece with a bacterial filter; (**B**) Average mass spectrum of exhaled breath without filter recorded from one subject; (**C**) Time traces of the plasticizer *m*/*z* 371.101 (decamethylcyclopentasiloxane) with and without filter from one subject; (**D**) Time traces of the plasticizer *m*/*z* 445.12 (dodecamethylcyclohexasiloxane) with and without filter from one subject.

In this study, the changes in breath profiles upon using a VOC filter for inspiration were assessed. We regarded measurements without a VOC filter as a common standard for the analysis by concordance correlation coefficients, since most previous clinical studies by SESI-HRMS were conducted without an inspiration VOC filter [13,14,15,16]. We chose strict criteria for the interpretation of the CCC, as recommended by Mahon [17]. Interestingly, almost 80% of the detected *m*/*z* features had a CCC > 0.9, which indicates that they had a moderate-to-substantial concordance between the measurements with and without the filter. Still, the bias across the entirety of the breath profiles towards lower intensities with the filter (Figure 1B) confirms that the filter resulted in generally decreased intensities of the 20% compounds with a CCC < 0.9. This is in line with our hypothesis. Figure 1C shows a trend towards decreased intensities in the heavier mass range. For *m*/*z* > 350, all *m*/*z* features had a negative location shift, whereas there was some scatter in both directions for the lighter features. Many of the compounds that are commonly detected in this heavy mass range are contaminants, mostly plasticizers, that originate from the ambient air. These plasticizers are usually present at rather high intensities (see Figure 5B). As the efficiency of a VOC filter increases with higher molecular weights and higher concentrations [18], this could explain the trend across the *m*/*z* axis.

### 3.1. Impact of Filter Usage on Breath Profiles

According to the concordance correlation coefficients, most metabolites had a moderate-to-perfect agreement and were therefore, by our definitions, not affected by the filter. A total of 11 of the targeted metabolites had less agreement with a CCC < 0.9 (Table 1), 9 of them with a decreased intensity after the filter usage. For example, the Bland–Altman and CCC analysis of 2-octenal (positive mode) and heptanoic acid (negative mode) in Figure 2 showed a loss in concordance due to signal intensity reduction after the filter application. Both compounds were present in ambient air, which confirms that they were filtered from ambient air. There were two metabolites, glycine and the water trimer, that showed a lower concordance and increased intensity after the filter usage. Possible explanations for such complex behavior are listed in the next paragraph. Interestingly, two fatty acids with a low concordance (see Table 1) were not detected in ambient air at all but still had decreased intensities, which we could not explain. Out of the 69 studied metabolites, 44 were detected in ambient air. However, only eight of them had a low concordance. The water cluster represents a special case: it had an increased intensity but was also detected in ambient air, since the air was artificially humidified before sampling and the electrospray itself creates (H_2_O)_2_H_3_O^+^. A total of 22 metabolites had a high concordance and were not detected in ambient air, which makes sense as they should not be affected by the filter. However, 36 metabolites with a high concordance were found in the room air samples and not affected by the filter. For example, although all 23 studied aldehydes were present in the ambient air, only 3 of them had a CCC < 0.9 and therefore a discrepancy before and after filter application. The time of pre-breathing in this study might have been too short to have an effect on the majority of aldehydes and some other metabolites. Basanta et al. investigated the effect of inhaling VOC-filtered air on aldehydes by measuring serial exhalations up to 30 min over time by gas chromatography time-of-flight mass spectrometry (GC–TOF-MS). They concluded that aldehydes were washing out slowly and only reached a steady state after more than 20 min of breathing filtered air [4].

All in all, the results indicate that the effect of the VOC filter on the studied metabolites was complex, since not even all metabolites that were decreased with the filter were found in the ambient air samples, some of those detected in ambient air had increased intensities, and not all metabolites that were found in ambient air had a bad concordance. Additionally, the effect of the filter on the studied metabolites was also not dependent on their concentrations in ambient air (Figure S4). There are several aspects that might be responsible for these findings: (1) The time of pre-breathing used in this study (only 10 exhalations) might have been too short to have a clear filtering effect on certain compounds [4,10,12]. (2) The effects of a VOC filter depend on various factors, for example, compound-specific factors such as chemical properties, their concentration in the ambient air and in breath, endogenous concentration, gas exchange and previous exposure [4,10,12]. Those factors might be more relevant for metabolites than for clearly exogenous contaminants. (3) Using a filter for inspiration might affect the breathing pattern. Although our set-up had no resistance and participants were instructed to breathe tidally as usual, there was an observed tendency to hypoventilation. This could have implications on the concentrations of the endogenously produced VOCs. However, a study concluded that the breathing pattern had no influence on electronic nose measurements [5]. (4) There is no internal standard in SESI-HRMS and it has not been assessed so far whether the intensities of the humidified ambient air samples are quantitatively comparable with those of breath samples. The sampling conditions of ambient air were approximated to those of breath, considering flow, humidity and temperature. However, the measured intensities are highly dependent on sampling conditions and might not be directly comparable to the intensities in breath. (5) The materials from the filter set-up (valves, mouthpiece, etc.) might release interfering contaminants. (6) The filter affects the composition of exhaled breath and reduces the total signal intensity, which could potentially affect the ionization process. Ion suppression is a known problem of electrospray ionization (ESI) that can diminish low-abundance features in the presence of high-abundance features, and multiple mechanisms for this phenomenon are plausible [19,20]. It is not known to what extent SESI is susceptible to ion suppression. The literature regarding extractive electrospray ionization (EESI), a method similar to SESI, suggests that ambient electrospray ionization is less prone to ion suppression [21]. However, ion suppression has been previously reported in some SESI publications [3,22]. Our results indicate that the effect of the filter does not majorly affect ion suppression. Still, it is interesting that the intensity of the water cluster and some other compounds (see Figure 1C,D) had increased intensities after the filter application, which could be caused by an altered distribution of charge. Further research on SESI and the potential mechanisms of ion suppression and its relevance are needed. 

It is worth emphasizing that many metabolites were found to be present in ambient air. Those metabolites should be treated with more caution in regard to clinical findings, since it might influence their exhaled concentrations.

### 3.2. Behavior of Contaminants after Filter Application

As expected, polysiloxanes were the largest group of contaminants with reduced intensities after the filter usage. Figure 5B highlights how dominant those plasticizers were in an average mass spectrum of breath in positive ionization mode and how they were reduced after filter application, visualized as time traces of a single measurement (Figure 5C,D). A representative polysiloxane (*m*/*z* +445.1195) is visualized as a Bland–Altman and a plot of the paired signal intensities without vs. with the filter (Figure 3C,D) shows a negative bias and a deviation from perfect agreement, which was stronger with a higher intensity. Apart from the plasticizers, the solvents methanol and acetamide (*m*/*z* +60.044, Figure 3A,B), which were abundant in ambient air, were also decreased with the filter. The impact of the filter on all of these contaminants was concentration-dependent, meaning that higher concentrations in ambient air resulted in larger within-subject differences before and after the filter usage (Figure 4A–D). Similar to the metabolites, there were also seven contaminants with a high concordance despite their presence in ambient air, which again suggests that the time of pre-breathing might not have been long enough to filter those compounds.

### 3.3. Comparison to Previous Studies

Previous studies using other breath analysis methodologies assessed the influence of ambient contaminants on breath measurements. In general, two different approaches were discussed: the simultaneous collection of ambient air samples and a subsequent correction based on the principles of alveolar gradients [23], or the inspiration of filtered or synthetic air before breath sampling [4,5,6,7,8,9,10,11]. The calculation of alveolar gradients yields information about the origin of the detected VOCs by subtracting the measured VOC concentrations of the inspired ambient air from those in breath samples [11,23]. The background subtraction of the environmental signal is easy to perform but does not lead to consistent results according to Schubert et al. [24]. Those concepts are simple and easily implemented, but they do not take complexities such as gas exchange, airway interactions and lung ventilation into account and are therefore of limited applicability [25]. Due to the limitations of ambient air sampling by SESI-HRMS mentioned above, we decided not to further explore the use of ambient air samples for the calculation of alveolar gradients or background correction in this study.

A recent study by Salman et al. investigated the variability of VOCs in clinical room air and suggested a novel approach for the treatment of ambient air contaminants by defining a minimal concentration threshold [26]. Most ambient air contaminants were only found at concentrations lower than 3 µg m^−3^ in their GC–MS data set, and they concluded that it was unlikely that the detected compounds with higher intensities were originating from the room air. In contrast to most other studies, Hewitt et al. assessed the influence of different clinical sampling environments on breath data and did not find a significant impact, concluding that the sampling location and varying VOC levels in room air do not affect GC–MS breath measurements [27].

### 3.4. Applicability of a VOC Filter in Pediatric Studies

Although the short time of pre-breathing might have limited the filtering efficiency, we chose a protocol with only 10 inspirations through the VOC filter on purpose. As our group usually performs studies in children, we aimed to test a procedure that would also be translatable to pediatric studies. A long duration of tidal breathing is not feasible for young children. During the conduction of the experiments, we noticed that even the adapted protocol with only 10 inspirations might not be applicable in children. We used a commercial filter attached to a customized non-rebreathing valve made of PEEK, as shown in Figure 5A. Using the VOC filter complicates the on-line breath measurement, which consists of several full exhalations into the instrument with short breaks in between. With the methodology of SESI-HRMS it is not possible to measure tidal exhalations, since the resistance is too high. Therefore, participants had to switch from the mouthpiece of the filter to the one of the SESI source several times during a measurement. Even with adults, some measurements had to be repeated, as some participants accidentally inspired ambient air at some point. We concluded that the implementation of the VOC filter for pediatric studies with young children is questionable as it would further complicate the measurements. The switching of mouthpieces is prone to bad sealing or involuntary inspirations without the filter, which would add unknown factors and make measurements less comparable. The issue of using a VOC filter for a prolonged period of time in terms of clinical applicability was also raised in a review by Miekisch et al. [25]. A recent study that compared different sampling methods even argued that using a clean air supply might lead to additional complexity and confounding factors [11].

## 4. Materials and Methods

### 4.1. Study Design and Participants

The observational study was outlined as a matched pair design, comparing measurements before and after using a VOC filter of each participant. A total of 24 adult and healthy staff members working at the University Children’s Hospital Zurich were recruited for this study. All participants were informed about the details of the study and gave written consent. Data collection was completed within two weeks and the individual appointments were equally distributed throughout the measurement period. Since the focus of this study was on comparing paired measurements of healthy adult participants, no clinical data were collected. The study was approved by the local ethics committee (KEK-ZH-Nr. 2014-0076). Written informed consent was obtained from all participants.

### 4.2. Breath Analysis Measurements

A high-resolution time-of-flight (TOF) mass spectrometer (TripleTOF 5600+, AB Sciex, Concord, ON, Canada) linked to a secondary electrospray ionization source (SuperSESI, FIT FossilionTech, Madrid, Spain) was used for breath analysis. The electrospray solution consisted of 0.1% (*v*/*v*) formic acid solution in water and was sprayed into the ionization source with 20 µm diameter silica emitters (PepSep PSFSE360-50-20 Fused Silica Emitter, Bruker, Billerica, MA, USA). Each participant performed two breath analysis measurements in a row with a short break in between. The first measurement was conducted without inhaling through the VOC filter. The second measurement was performed with a VOC filter for inspiration. A filter of the type A2 (X-plore^®^ Rd40 940, A2, 67 38 855, Dräger, Lübeck, Germany) was used, which is designed to filter out organic compounds with a boiling point above 65 °C in gases or vapors. The filter was connected to a custom-made two-way non-rebreathing T-valve with an inner diameter of 15 mm. The T-piece was made from polyether ether ketone (PEEK) with two built-in diaphragm valves (835900, Hans Rudolph, Inc., Shawnee, KS, USA) to ensure that the airflow was unidirectional and only filtered air was inspired. The T-piece with the connected filter was constructed to provide as little resistance as possible. A spirometry filter (MADA Spirometry Filter 83, amc-30864, amc advanced metabolic control ag, Engelberg, Switzerland) was attached to it as a mouthpiece and to ensure that the T-piece was not contaminated by exhaled viruses or bacteria. The filter set-up is shown in Figure 5A. Each breath analysis measurement consisted of 4 long and complete exhalations at a constant defined pressure at 5 mbar with short breaks in between each exhalation. For the measurement with the VOC filter, the participants were asked to ex- and inspire at least ten times through the T-piece attached to the filter. It was emphasized that the breathing pattern through the filter should be normal tidal breathing to prevent hyperventilation. After the ten tidal inspirations through the filters, the participants were instructed to take a full inspiration through the filter and immediately switch to the mouthpiece connected to the ionization source and exhale into the instrument as during the previous measurement without filter.

### 4.3. Ambient Air Samples

Ambient air samples were collected during the course of the study at several time points spread across each day of measurements. The sampling conditions were approximated to those of breath sampling. The ambient air was heated to 40 °C and humidified before being analyzed at a flow of 0.3 L per minute by SESI-HRMS by attaching a vacuum pump to the exhaust of the ionization source. The *m*/*z* features from the ambient air samples above an intensity cut-off of 50 counts per second (cps) were matched to the features in breath. The studied metabolites and contaminants were searched in the ambient air features. Details about the processing can be found in the Supplementary Material.

### 4.4. Data Processing and Data Analysis

The breath profiles resulting from the mass spectral measurements were determined in the same way as described previously [13,28,29]. A more detailed exposition can be found in the Supplementary Material. Briefly, peak picking was implemented on the interpolated, averaged mass spectra corresponding to exhalation maneuvers. Trapezoidal integration was used to determine the signal intensities of the *m*/*z* features. Furthermore, *m*/*z* features that correlated with breath exhalation maneuvers and were present in at least 80% of all measurements were selected, log2-transformed and congregated into a matrix of breath profiles with and without filter usage for subsequent analysis. The humid air samples were processed in parallel in the same manner to obtain a list of features measured in the ambient air. The features in humid air samples with the raw intensity above 50 counts per second (cps) and ppm < 10 difference with features from human breath profiles were kept for examination.

An overall assessment of the agreement between the two methods (without and with the VOC filter) was performed by computing the CCCs [30]. Furthermore, Bland–Altman analysis [31] was performed on *m*/*z* features corresponding to previously reported human metabolites in SESI-HRMS experiments. A list of 88 compounds (50 in positive and 38 in negative ionization mode) from various chemical families was used in order to cover the broad range of compounds that are typically detectable in human breath with SESI-HRMS [14,15,16,32,33,34,35,36,37,38,39,40]. More details can be found in the Supplementary Table S1. In addition to known human metabolites, the influence of the filter on common contaminants from the ambient air was assessed. For this, a second list containing 54 volatile contaminants (42 in positive, 12 in negative ionization mode) was prepared based on a list of mass spectrometry contaminants from Keller et al. [41], including solvents, plasticizers and acids while excluding Na or Cu ionization adducts of these. Acetamide (*m*/*z* +60.044, identified by exact mass matching) was additionally added to the list, since this is a known airborne contaminant of our lab.

## 5. Conclusions

The results support our initial hypothesis that using a filter for inspiration decreases the intensity of high-intensity plasticizers, contaminants and some metabolites present in ambient air, measured by on-line SESI-HRMS. We also conclude from this feasibility study that using a filter for inspiration complicates the conduction of breath analysis measurements, even if only performing 10 tidal inspirations in advance. It is not possible to include a VOC filter for on-line breath analysis studies by SESI-HRMS in a young pediatric study population. 

The findings support our hypothesis that applying a VOC filter for on-line breath analysis measurements by SESI-HRMS reduces the intensity of plasticizers, contaminants and some airborne metabolites. Still, most *m*/*z* features including many metabolites had a substantial-to-moderate agreement with and without the filter. We recommend not to mix measurements with and without a filter within one study, as the data might not be comparable. Most importantly, we conclude that implementing a VOC filter in pediatric SESI-HRMS studies is not feasible. Nevertheless, this study showed that it might be beneficial to take measures to reduce the influence of ambient air on on-line breath analysis measurements. The simultaneous collection of air samples allows for the monitoring of changes in the composition of room air over time. Compounds with constant high intensities in ambient air, such as polysiloxanes, could then be excluded from data analysis. Additionally, relevant metabolites could be tracked in the ambient air to assess their robustness.

## Figures and Tables

**Table 1:** Exhaled metabolites with a CCC < 0.9. The location shift and Bland–Altman bias indicate whether the intensity was increased (positive values) or decreased (negative values) with filter usage.

Mode	*m*/*z*	Compound	CCC	Location Shift	Bland–Altman Bias	Elevated In	In Ambient Air
neg	129.0915	Heptanoic acid	0.51	−1.18	−0.79	No filter	Yes
neg	143.107	Octanoic acid	0.56	−1.14	−0.86	No filter	Yes
pos	127.111	2-Octenal	0.57	−1.04	−0.87	No filter	Yes
pos	55.039	Water trimer	0.63	0.47	0.12	Filter	Yes
pos	253.2145	4-Hydroxy-2,6-hexadecadienal	0.83	−0.15	−0.13	No filter	Yes
pos	143.106	4-Hydroxy-2-octenal	0.83	−0.51	−0.23	No filter	Yes
neg	199.134	ω-Oxoundecanoic acid	0.85	−0.51	−0.52	No filter	Yes
pos	116.07	Proline	0.88	−0.29	−0.14	No filter	No
neg	157.087	ω-Oxooctanoic acid	0.89	−0.47	−0.41	No filter	Yes
neg	159.1025	ω-Hydroxyoctanoic acid	0.89	−0.45	−0.45	No filter	No
pos	76.039	Glycine	0.89	0.13	0.06	Filter	No

**Table 2:** Environmental contaminants that had a CCC < 0.9 agreement before and after filter usage. The location shift and Bland–Altman bias indicate whether the intensity was increased (positive values) or decreased (negative values) with filter usage.

Mode	*m*/*z*	Compound	CCC	Location Shift	Bland–Altman Bias	Elevated In	In Ambient Air
pos	388.128	Polysiloxane	0.59	−1.07	−1.56	No filter	Yes
pos	60.044	Acetamide	0.61	−0.99	−0.98	No filter	Yes
pos	65.0595	Methanol dimer	0.67	−0.63	−0.67	No filter	Yes
pos	355.069	Polysiloxane	0.71	−0.78	−1.17	No filter	Yes
pos	371.101	Polysiloxane	0.73	−0.73	−1.20	No filter	Yes
pos	429.088	Polysiloxane	0.77	−0.68	−0.80	No filter	Yes
pos	462.147	Polysiloxane	0.78	−0.68	−1.00	No filter	Yes
pos	445.1195	Polysiloxane	0.85	−0.50	−0.68	No filter	Yes
pos	149.023	Fragment of phthalate esters	0.88	0.00	−0.00	-	No
pos	74.0595	Dimethyl formamide	0.89	−0.36	−0.30	No filter	Yes

## Data Availability

The data presented in this study are openly available in FigShare at https://doi.org/10.6084/m9.figshare.20601930.

## Supplementary Materials

The following supporting information can be downloaded at: https://www.mdpi.com/article/10.3390/molecules28010045/s1, Table S1: List of all studied metabolites including their concordance correlation coefficient, location shift and bias from Bland–Altman analysis; Table S2: List of all studied contaminants including their concordance correlation coefficient, location shift and Bland-Altman bias. Figure S1: Bland–Altman plots and CCC plots of all detected metabolites in exhaled breath samples; Figure S2: Bland–Altman and CCC plots for all detected contaminants in the exhaled breath profiles; Figure S3: Bland–Altman and CCC plots for all detected contaminants in the exhaled breath profiles. Figure S4: Plots consisting of metabolites found in ambient air samples measured immediately together with human breath samples (7 plot points).

## Sample Availability

There are no samples available.
