# Supplementary material for: Effects of a Volatile Organic Compound Filter on Breath Profiles Measured by Secondary Electrospray High-Resolution Mass Spectrometry
Source: Molecules. 2022 Dec 21;28(1):45. doi: 10.3390/molecules28010045 (PMC9822030; doi:10.3390/molecules28010045)
Supplement: Supplementary file 1 [file molecules-28-00045-s001.zip › molecules-2065082-supplementary.pdf]

Supplementary material - Effects of a volatile organic compound filter on breath profiles measured by secondary electrospray high-resolution mass spectrometry

Ronja Weber[1], Jérôme Kaeslin[2], Sophia Moeller[1], Nathan Perkins[3], Srdjan Micic[1*], Alexander Moeller[1,4,*]

[1] Department of Respiratory Medicine and Childhood Research Center, University Children's Hospital Zurich, Steinwiesstrasse 75, 8032 Zurich, Switzerland
[2] Department of Chemistry and Applied Biosciences, Swiss Federal Institute of Technology, Vladimir-Prelog Weg 3, 8093 Zurich, Switzerland
[3] Division of Clinical Chemistry and Biochemistry, University Children's Hospital Zurich, Steinwiesstrasse 75, 8032 Zurich, Switzerland
[4] Faculty of Medicine, University of Zurich, Raemistrasse 71, 8006 Zurich, Switzerland

* These authors contributed equally to this work.

Corresponding authors:

Alexander Moeller
Steinwiesstrasse 75
8032 Zurich
Switzerland
+41(0)442667079
alexander.moeller@kispi.uzh.ch

Srdjan Micic
Steinwiesstrasse 75
8032 Zurich
Switzerland
+41(0)442667079
srdjan.micic@kispi.uzh.ch

1. Supplementary information

1.1 Data pre-processing

The following section serves as a summary of the raw mass spectra processing pipeline as already implemented in our previous work [1–3].

The recorded raw mass spectra were aligned using the proprietary PeakView software (Version 2.2, Applied Biosystems Sciex, Toronto, ON, Canada) and afterwards converted from the proprietary wiff file format into the open file standard mzXML, using the MSConvert (Version 3, ProteoWizard Tools, Palo Alto, CA, US). Further processing was done in R v4.1.1 (R Foundation for Statistical Computing, Vienna, Austria). Piecewise cubic Hermite interpolation was applied to resample the mass spectra associated with the measurements without filter usage onto a linearly spaced *m/z*-axis with a Δ*m/z* step-size of 0.0005. Subsequently, trapezoidal integration was applied to compute the total ion chromatograms (TIC), which were subsequently used to distinguish the mass spectral scans corresponding to exhaled breath from the ones of the breaks between exhalations.

Peak picking was done for each subject individually on the mass spectrum averaged over all scans corresponding to exhalations where signal-to-noise ratio (SNR) was set to 2 with Friedman's super smoother calculated over the mass spectrum. As a next step, the peak positions across the different participants were combined into a single list, from which a kernel density estimate (Gaussian kernel, bandwidth = 0.0025) was calculated. The local maxima of the smoothed density function were used to compensate for small variations in the peak positions and to define the final list of *m/z* features that are representative for all measurements. Centroiding was performed by trapezoidal integration (mass window *m/z* ± Δ*m/z*, Δ*m/z* = 0.0025), which yielded time traces of *m/z* feature intensities for each sample. m/z features which were not associated with exhalation manoeuvres were eliminated by a simple linear regression model, which was built for each measurement between the standardised TIC and the standardised time trace of the individual *m/z* features. In case the estimated slope was < 0, the *m/z* features were not included in the final list. As an additional filtering parameter to restrict the number of included features to the ones associated with exhalations, the ratio of the sum of squares of the predicted values by the simple linear regression model of the *m/z* feature over the scans and the sum of squares of the standardised TIC was used. For both positive and negative ionization mode, 0.5 was determined as the minimal threshold for the ratio. Lastly, in order to eliminate features that were not consistently present across all samples, only *m/z* features which were present in at least 80% of all measurements were included for further processing steps. The signal intensities of the remaining *m/z* features were calculated by averaging over scans corresponding to exhalations and log2 transformed. The features were then depicted in the mass spectra recorded without filter usage and arranged into a matrix 2n x k matrix where n is the number of participants and k is the number of *m/z* features.

In the same manner, the humid air samples were processed to obtain a list of features and their log2 intensities coming from the ambient air. Additionally, features with the raw intensity of 50 counts per second (cps) and lower were excluded from the list. Matching of the *m/z* features found in human breath samples was done based on peak position differences. The threshold of 10 ppm and lower was used to find the matching features in both groups (human samples and humid air samples).

2. Supplementary tables

2.1 Table S1

List of all studied metabolites including their concordance correlation coefficient, location shift and bias from Bland-Altman analysis.

Mod.	*m/z*	Compound	Formula	Ion.	CCC	LSt	BA bias	Elev. in	Amb. air	Ref.
neg	129.0915	Heptanoic acid	C7H14O2	[M-H]-	0.51	-1.18	-0.79	No filter	Yes	[4]
neg	143.107	Octanoic acid	C8H16O2	[M-H]-	0.56	-1.14	-0.86	No filter	Yes	[4]
pos	127.111	2-Octenal	C8H14O	[M+H]+	0.57	-1.04	-0.87	No filter	Yes	[5,6]
pos	55.039	Water trimer	H2O	[M3+H]+	0.63	0.47	0.12	Filter	Yes	-
pos	253.2145	4-Hydroxy-2,6-hexadecadienal	C16H28O2	[M+H]+	0.83	-0.15	-0.13	No filter	Yes	[5,6]
pos	143.106	4-Hydroxy-2-octenal	C8H14O2	[M+H]+	0.83	-0.51	-0.23	No filter	Yes	[5,6]
neg	199.134	ω-Oxoundecanoic acid	C11H20O3	[M-H]-	0.85	-0.51	-0.52	No filter	No	[7]
pos	116.07	Proline	C5H9NO2	[M+H]+	0.88	-0.29	-0.14	No filter	Yes	[8]
neg	157.087	ω-Oxooctanoic acid	C8H14O3	[M-H]-	0.89	-0.47	-0.41	No filter	Yes	[7]
neg	159.1025	ω-Hydroxyoctanoic acid	C8H14O3	[M-H]-	0.89	-0.45	-0.41	No filter	No	[7]
pos	76.039	Glycine	C2H5NO	[M+H]+	0.89	0.13	0.06	Filter	No	[8]
pos	166.0865	Phenylalanine	C9H11NO2	[M+H]+	0.91	-0.31	-0.20	No filter	No	[8]
pos	77.0595	Acetone + H2O	C3H6O	[M+H2O +H]+	0.91	0.29	0.21	Filter	Yes	-
pos	211.169	4-Hydroxy-2,6-tridecadienal	C13H22O2	[M+H]+	0.91	-0.34	-0.15	No filter	Yes	[5,6]
neg	130.998	Oxaloacetic acid	C4H4O5	[M-H]-	0.91	0.11	0.08	Filter	No	[9]
pos	59.049	Acetone	C3H6O	[M+H]+	0.91	0.36	0.28	Filter	No	[10]
neg	157.123	Nonaoic acid	C9H18O2	[M-H]-	0.92	-0.32	-0.42	No filter	Yes	[4]
pos	197.1535	4-Hydroxy-2,6-dodecadienal	C12H20O2	[M+H]+	0.92	-0.15	-0.08	No filter	Yes	[5,6]
neg	173.0815	Octanedioic acid	C8H14O4	[M-H]-	0.92	-0.35	-0.31	No filter	Yes	[11]
neg	143.071	ω-Oxoheptanoic acid	C7H12O3	[M-H]-	0.92	-0.34	-0.29	No filter	Yes	[7]
neg	115.076	Hexanoic acid	C6H12O2	[M-H]-	0.93	-0.25	-0.27	No filter	Yes	[12]
pos	171.1375	4-Hydroxy-2-decenal	C10H18O2	[M+H]+	0.93	-0.31	-0.22	No filter	Yes	[5,6]
pos	157.122	4-Hydroxy-2-nonenal	C9H16O2	[M+H]+	0.93	-0.27	-0.15	No filter	Yes	[5,6]
pos	239.2	4-Hydroxy-2,6-pentadecadienal	C15H26O2	[M+H]+	0.93	0.12	0.06	Filter	Yes	[5,6]
neg	129.055	ω-Oxohexanoic acid	C6H10O3	[M-H]-	0.94	-0.28	-0.20	No filter	Yes	[7]
pos	213.1845	4-Hydroxy-2-tridecenal	C13H24O2	[M+H]+	0.94	-0.22	-0.16	No filter	Yes	[5,6]

neg	145.05	Hexanedioic acid	C6H10O4	[M-H]-	0.94	-0.02	-0.01	No filter	No	[7]
pos	227.2005	4-Hydroxy-2-tetradecenal	C14H26O2	[M+H]+	0.94	-0.06	-0.03	No filter	Yes	[5,6]
pos	225.1845	4-Hydroxy-2,6-tetradecadienal	C14H24O2	[M+H]+	0.94	-0.12	-0.06	No filter	Yes	[5,6]
neg	159.066	Heptanedioic acid	C7H12O4	[M-H]-	0.94	-0.27	-0.27	No filter	No	[7]
pos	199.1695	4-Hydroxy-2-dodecenal	C12H22O2	[M+H]+	0.94	-0.19	-0.09	No filter	Yes	[5,6]
neg	173.118	ω-Hydroxynonanoic acid	C9H18O3	[M-H]-	0.94	-0.27	-0.33	No filter	Yes	[7]
pos	90.0545	Alanine	C3H7NO2	[M+H]+	0.94	-0.03	-0.02	No filter	No	[8]
neg	171.102	ω-Oxononanoic acid	C9H16O3	[M-H]-	0.95	-0.26	-0.34	No filter	Yes	[7]
pos	185.153	4-Hydroxy-2-undecenal	C11H20O2	[M+H]+	0.95	-0.27	-0.13	No filter	Yes	[5,6]
pos	132.1015	(Iso)leucine	C6H13NO2	[M+H]+	0.95	-0.21	-0.17	No filter	Yes	[8]
pos	104.07	Aminobutanoate	C4H9NO2	[M+H]+	0.95	-0.15	-0.09	No filter	No	[11]
pos	141.1265	2-Nonenal	C9H16O	[M+H]+	0.95	-0.13	-0.11	No filter	Yes	[5,6]
neg	187.097	Nonanedioic acid	C9H16O4	[M-H]-	0.95	-0.22	-0.28	No filter	No	[7]
pos	183.1375	4-Hydroxy-2,6-undecadienal	C11H18O2	[M+H]+	0.95	-0.14	-0.08	No filter	Yes	[5,6]
neg	91.0035	Formic acid dimer	CH2O2	[M2-H]-	0.96	0.10	0.03	Filter	Yes	-
neg	115.003	Fumaric acid	C4H4O4	[M-H]-	0.96	0.05	0.04	Filter	No	[9]
neg	185.118	ω-Oxodecanoic acid	C10H18O3	[M-H]-	0.96	-0.18	-0.24	No filter	Yes	[7]
pos	218.1385	Propionylcarnitine	C10H19NO4	[M+H]+	0.96	-0.17	-0.13	No filter	No	[11]
pos	141.09	4-Hydroxy-2,6-octadienal	C8H12O2	[M+H]+	0.97	-0.10	-0.06	No filter	Yes	[5,6]
pos	118.065	Indole	C8H7N	[M+H]+	0.97	0.15	0.21	Filter	No	[10]
neg	117.019	Succinic acid	C4H6O4	[M-H]-	0.97	-0.03	-0.03	No filter	No	[9]
neg	133.014	Malic acid	C4H6O5	[M-H]-	0.97	-0.03	-0.02	No filter	No	[9]
pos	183.174	2-Dodecenal	C12H22O	[M+H]+	0.97	-0.06	-0.04	No filter	Yes	[5,6]
neg	131.0345	Pentanedioic acid	C5H8O4	[M-H]-	0.97	-0.08	-0.07	No filter	No	[7]
pos	204.123	Acetylcarnitine	C9H17NO4	[M+H]+	0.97	-0.13	-0.11	No filter	No	[11]
neg	167.0195	Uric acid	C5H4N4O3	[M-H]-	0.97	-0.10	-0.09	No filter	No	[13]
pos	211.2055	2-Tetradecenal	C14H26O	[M+H]+	0.97	0.04	0.03	Filter	Yes	[5,6]
neg	101.06	Pentanoic acid	C5H10O2	[M-H]-	0.97	0.03	0.04	Filter	Yes	[12]
neg	145.014	α-Ketoglutaric acid	C5H6O5	[M-H]-	0.97	-0.03	-0.02	No filter	Yes	[9]
pos	169.1585	2-Undecenal	C11H20O	[M+H]+	0.97	-0.08	-0.07	No filter	Yes	[5,6]
pos	239.2365	2-Hexadecenal	C16H30O	[M+H]+	0.98	0.02	0.02	Filter	Yes	[5,6]
neg	149.0455	Pentose	C5H10O5	[M-H]-	0.98	0.05	0.06	Filter	No	[11]
pos	155.106	4-Hydroxy-2,6-nonadienal	C9H14O2	[M+H]+	0.98	-0.09	-0.05	No filter	Yes	[5,6]

neg	173.0085	Aconitic acid	C6H6O6	[M-H]-	0.98	-0.01	-0.01	No filter	No	[7]
neg	179.0555	Hexose	C6H12O6	[M-H]-	0.98	0.03	0.05	Filter	No	[11]
pos	225.2215	2-Pentadecenal	C15H28O	[M+H]+	0.98	0.00	0.00	Filter	Yes	[5,6]
neg	181.071	Hexitol	C6H14O6	[M-H]-	0.98	0.02	0.03	Filter	No	[11]
pos	118.0855	Valine	C5H11NO2	[M+H]+	0.98	-0.03	-0.03	No filter	Yes	[8]
pos	155.1425	2-Decenal	C10H18O	[M+H]+	0.99	0.03	0.07	Filter	Yes	[5,6]
pos	169.122	4-Hydroxy-2,6-decadienal	C10H16O2	[M+H]+	0.99	-0.01	-0.01	No filter	Yes	[5,6]
pos	197.1895	2-Tridecenal	C13H24O	[M+H]+	0.99	0.02	0.02	Filter	Yes	[5,6]
neg	183.004	2,4-Dinitrophenol	C6H4N2O5	[M-H]-	0.99	0.05	-0.05	Filter	No	[13]
neg	197.0205	2-Methyl-4,6-dinitrophenol	C7H6N2O5	[M-H]-	0.99	-0.01	-0.01	No filter	No	[13]
Not found in data										
pos	69.0681	Isoprene	C5H8	[M+H]+						[14]
pos	106.0499	Serine	C3H7NO3	[M+H]+						[8]
pos	133.0607	Asparagine	C4H8N2O3	[M+H]+						[8]
pos	133.0971	Ornithine	C5H12N2O2	[M+H]+						[8]
pos	147.0764	Glutamine	C5H10N2O3	[M+H]+						[8]
pos	147.1128	Lysine	C6H14N2O2	[M+H]+						[8]
pos	175.1189	Arginine	C6H14N4O2	[M+H]+						[8]
pos	176.0706	Indole-3-acetate	C10H9NO2	[M+H]+						[15]
pos	206.0812	5-Methoxyindoleacetate	C11H11NO3	[M+H]+						[15]
pos	225.087	3-Hydroxy-L-kynurenine	C10H12N2O4	[M+H]+						[15]
pos	241.2162	4-Hydroxy-2-pentadecenal	C15H28O2	[M+H]+						[5,6]
pos	255.2319	4-Hydroxy-2-hexadecenal	C16H30O2	[M+H]+						[5,6]

Abbreviations: Mod. = Ionization mode, Ion. = Ionization, CCC = Concordance correlation coefficient, LS = Location shift, BA = Bland-Altman, Elev. in = Elevated in, Amb. air = Ambient air, Ref = Reference

2.2 Table S2

List of all studied contaminants including their concordance correlation coefficient, location shift and Bland-Altman bias.

**Mod.**	***m/z***	**Compound**	**Formula**	**Ion.**	**CCC**	**LS**	**BA bias**	**Elev. in**	**Amb. air**
pos	388.128	Polysiloxane	[C2H6SiO]5	[M+NH4]+	0.59	-1.07	1.56	No filter	Yes
pos	60.044	Acetamide	C2H5NO	[M+H]+	0.61	-0.99	0.98	No filter	Yes

pos	65.0595	Methanol	CH3OH	[M2+H]+	0.67	-0.63	0.67	No filter	Yes
pos	355.069	Polysiloxane	[C2H6SiO]5	[M+H-CH4]+	0.71	-0.78	1.17	No filter	Yes
pos	371.101	Polysiloxane	[C2H6SiO]5	[M+H]+	0.73	-0.73	1.20	No filter	Yes
pos	429.088	Polysiloxane	[C2H6SiO]6	[M+H-CH4]+	0.77	-0.68	0.80	No filter	Yes
pos	462.147	Polysiloxane	[C2H6SiO]6	[M+NH4]+	0.78	-0.68	1.00	No filter	Yes
pos	445.1195	Polysiloxane	[C2H6SiO]6	[M+H]+	0.85	-0.50	0.68	No filter	Yes
pos	149.023	Fragment of phthalate esters	C8H4O3	[f+H]+	0.88	0.00	0.00	Filter	No
pos	74.0595	Dimethyl formamide	C3H7NO	[M+H]+	0.89	-0.36	0.30	No filter	Yes
pos	195.064	Dimethyl phthalate	C10H10O4	[M+H]+	0.91	-0.26	0.16	No filter	Yes
pos	102.0545	Acetonitrile/Acetic Acid	(CH3CN)n(CH3COOH)m	[A1B1+H]+	0.93	-0.20	0.08	No filter	Yes
pos	279.1595	Dibutylphthalate	C16H22O4	[M+H]+	0.93	0.15	-0.12	Filter	Yes
pos	88.0385	Acetonitrile/Formic Acid	(CH3CN)n(HCOOH)m	[A1B1+H]+	0.94	0.13	-0.05	Filter	No
neg	255.2325	Palmitic acid	C16H32O2	[M-H]-	0.96	-0.19	0.24	No filter	Yes
pos	79.0205	Dimethylsulfoxide	C2H6OS	[M+H]+	0.97	-0.09	0.08	No filter	No
neg	283.264	Stearic acid	C18H36O2	[M-H]-	0.97	-0.19	0.26	No filter	Yes
neg	78.9585	Phosphoric acid	H3PO4	[M-H2O-H]-	0.98	-0.06	0.04	No filter	No
pos	123.091	Dimethylaminopyridine	C7H10N2	[M+H]+	0.99	-0.05	0.06	No filter	No
neg	96.969	Phosphoric acid	H3PO4	[M-H]-	0.99	-0.07	0.05	No filter	Yes
neg	96.9595	Sulfuric acid	H3PO4	[M-H]-	0.99	-0.03	0.07	No filter	No
pos	100.075	N-methyl 2-pyrrolidone	C5H10NO	[M+H]+	0.99	-0.03	0.04	No filter	No
neg	94.9805	Methanesulfuric acid	CH3SO3H	[M-H]-	0.99	0.02	-0.03	Filter	Yes
Not found in data									
pos	59.0603	Acetonitrile	CH3CN	[M+NH4]+					
pos	74.0595	Acetonitrile/Methanol	(CH3CN)n(CH3OH)m	[A1B1+H]+					
pos	83.0603	Acetonitrile	CH3CN	[M2+H]+					
pos	85.0588	d6-Dimethylsulfoxide	C2D6OS	[M+H]+					
pos	101.0031	Dimethylsulfoxide	C2H6OS	[M+Na]+					
pos	101.0808	Methanol/Water	[MeOH]n[H2O]m	[A2B2+H]+					
pos	115.0865	Acetonitrile/Dimethyl formamide	(CH3CN)n(C3H7NO)m	[A1B1+H]+					
pos	120.0477	Dimethylsulfoxide	C2H6OS	[M+CH3CN+H]+					
pos	130.159	Diisopropylethylamine	C8H19N	[M+H]+					
pos	133.107	Methanol/Water	[MeOH]n[H2O]m	[A3B2+H]+					

pos	137.0743	Dimethylsulfoxide	C2H6OS	[M+CH3CN +NH4]+					
pos	144.1746	Tripropylamine	C9H21N	[M+H]+					
pos	147.1128	Acetonitrile/Methano l	(CH3CN)n(CH3OH)m	[A2B2+H]+					
pos	150.1277	Phenyldiethylamine	C10H15N	[M+H]+					
pos	157.0351	Dimethylsulfoxide	C2H6OS	[M2+H]+					
pos	163.0389	Dimethyl phthalate	C10H10O4	[M-CH3OH+H]+					
pos	169.1104	d6-Dimethylsulfoxide	C2D6OS	[M2+H]+					
pos	183.1438	Methanol/Water	[MeOH]n[H2O]m	[A4B3+H]+					
pos	186.2216	Tributylamine	C12H27N	[M+H]+					
pos	214.0896	n-butyl benzenesulfonamide	C10H15NO2S	[M+H]+					
pos	231.1161	n-butyl benzenesulfonamide	C10H15NO2S	[M+NH4]+					
pos	315.2529	Dibutyl sebacate	C18H34O4	[M+H]+					
pos	371.3155	Bis(2-ethylhexyl) adipate	C22H42O4	[M+H]+					
pos	371.3155	Diisooctyl phthalate	C22H42O4	[M+H]+					
pos	391.2842	Diisooctyl phthalate	C24H38O4	[M+H]+					
neg	59.0138	Acetic acid	CH3COOH	[M-H]-					
neg	79.9573	Sulfuric acid	H2SO4	[M-H2O]-					
neg	112.9855	Trifluoroacetic acid	CF3COOH	[M-H]-					
neg	162.9824	Pentafluoropropionic acid	CF3CF2COOH	[M-H]-					
neg	226.9784	Trifluoroacetic acid	CF3COOH	[M2-H]-					
neg	281.2486	Oleic acid	C18H34O2	[M-H]-					

All contaminants except for acetamide (ID5, contaminant of our lab) were extracted from the list of Keller et al. [16].
Abbreviations: Mod. = Ionization mode, Ion. = Ionization, CCC = Concordance correlation coefficient, LS = Location shift, BA = Bland-Altman, Elev. in = Elevated in, Amb. air = Ambient air, Ref = Reference

3. Supplementary figures

3.1 Figure S1

Bland-Altman plots and CCC plots of all detected metabolites in exhaled breath samples. Plots on the left represent Bland-Altman plots with bias (mean of with filter - without filter differences) as dashed line enclosed in either pink or blue colored ribbon representing. Pink colour indicates a negative bias, i.e. signal intensity reduction with filter usage and blue colour indicates a positive bias, i.e. signal intensity increase with filter usage. The ribbon itself is equal to the 95% confidence interval of the bias. The top and the bottom lines represent the

agreement limits, i.e. +/- 1.96s, where s is the standard deviation of differences. The horizontal black line represents the 0 difference. Plots on the right capture signal intensities without filter usage (x-axis) and with filter usage (y-axis). The diagonal black line represents the line of perfect agreement. The dashed line represents the simple linear regression for signal intensities with filter usage as a function of intensities without filter usage. Included in the bottom right corner are concordance correlation coefficient (CCC), the location shift and the scale shift. The plots are ordered by CCC (from lower to higher).

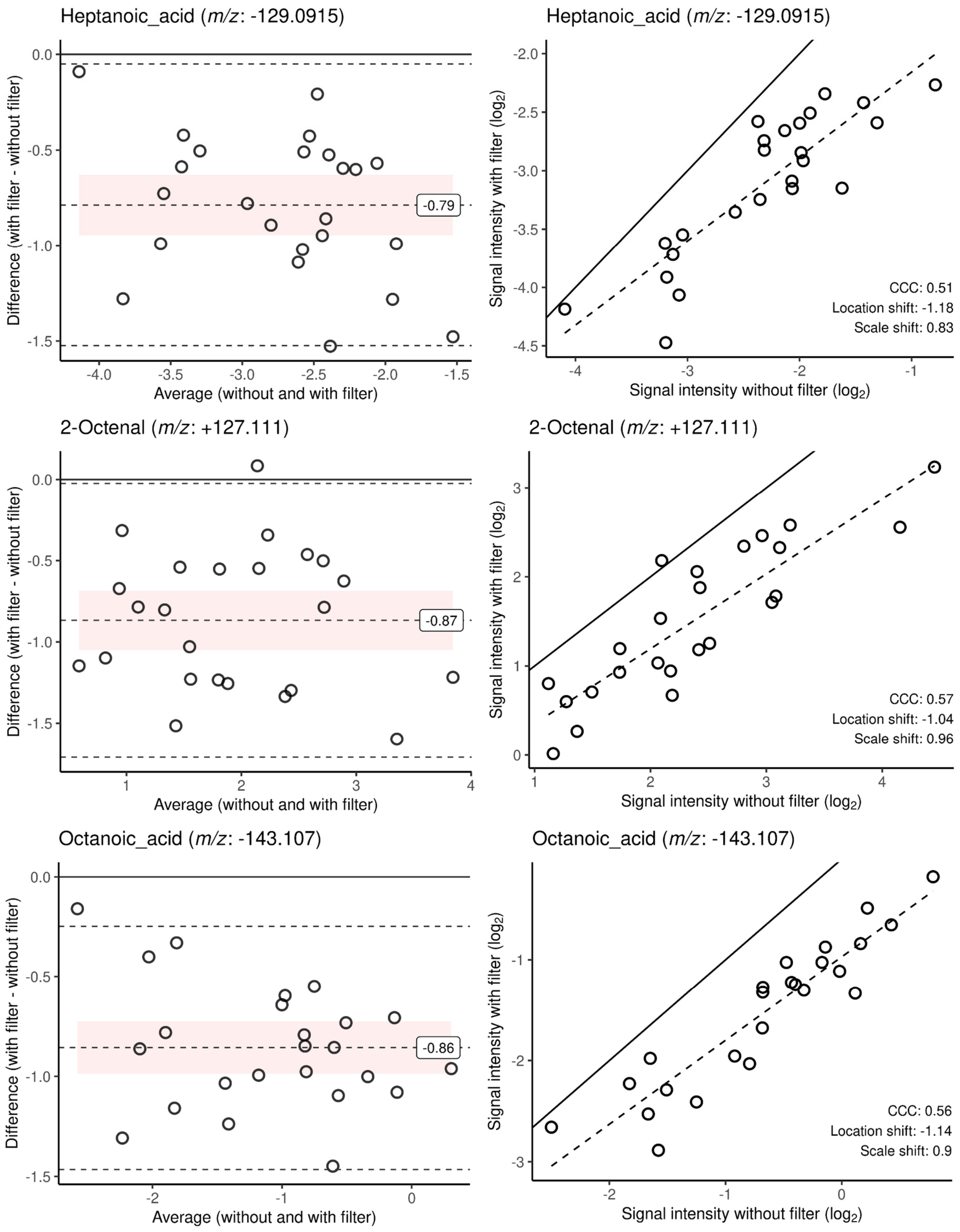

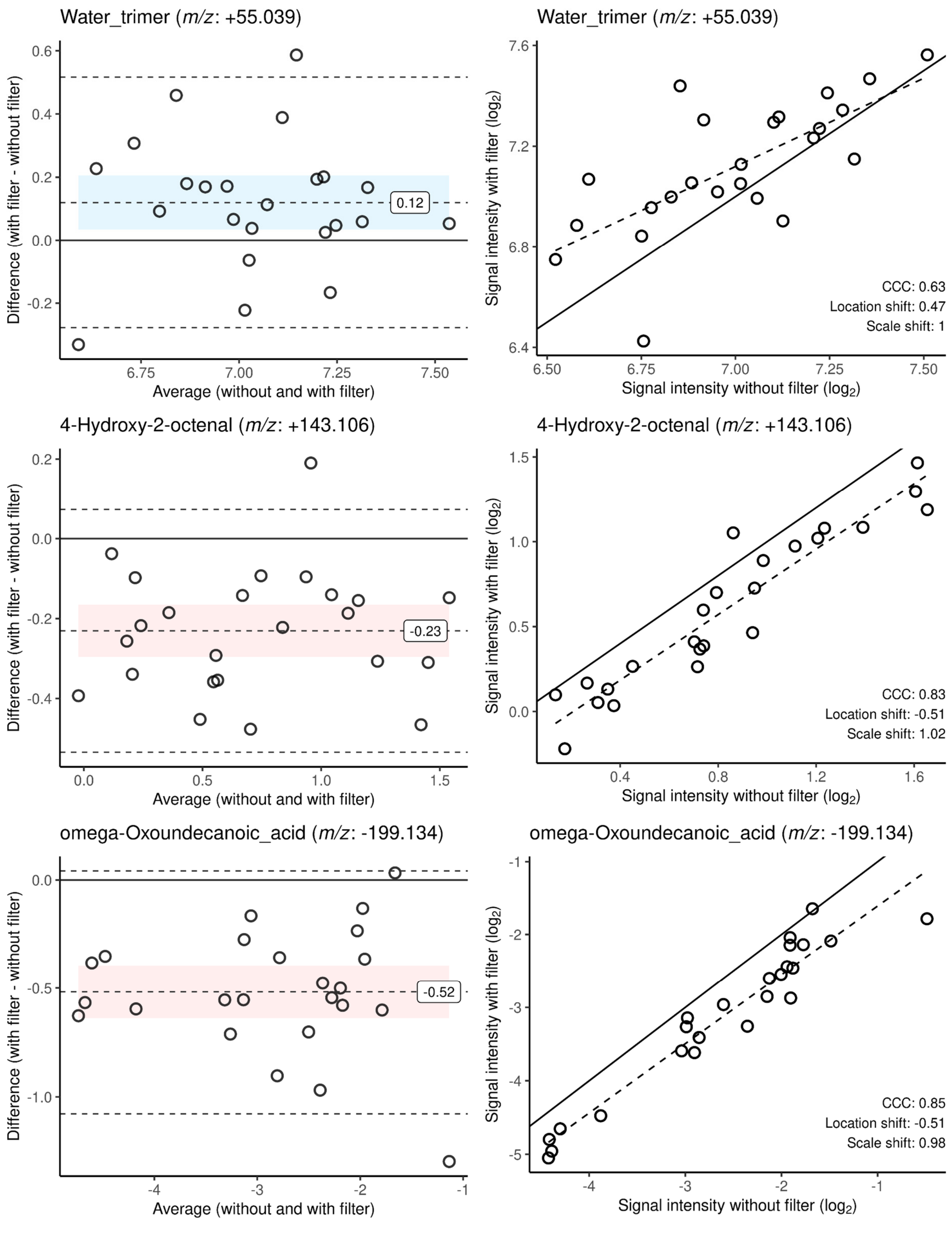

Water_trimer (m/z: +55.039)
Difference (with filter - without filter)
Average (without and with filter)
0.12
Water_trimer (m/z: +55.039)
Signal intensity with filter (log2)
Signal intensity without filter (log2)
CCC: 0.63
Location shift: 0.47
Scale shift: 1
4-Hydroxy-2-octenal (m/z: +143.106)
Difference (with filter - without filter)
Average (without and with filter)
-0.23
4-Hydroxy-2-octenal (m/z: +143.106)
Signal intensity with filter (log2)
Signal intensity without filter (log2)
CCC: 0.83
Location shift: -0.51
Scale shift: 1.02
omega-Oxoundecanoic_acid (m/z: -199.134)
Difference (with filter - without filter)
Average (without and with filter)
-0.52
omega-Oxoundecanoic_acid (m/z: -199.134)
Signal intensity with filter (log2)
Signal intensity without filter (log2)
CCC: 0.85
Location shift: -0.51
Scale shift: 0.98

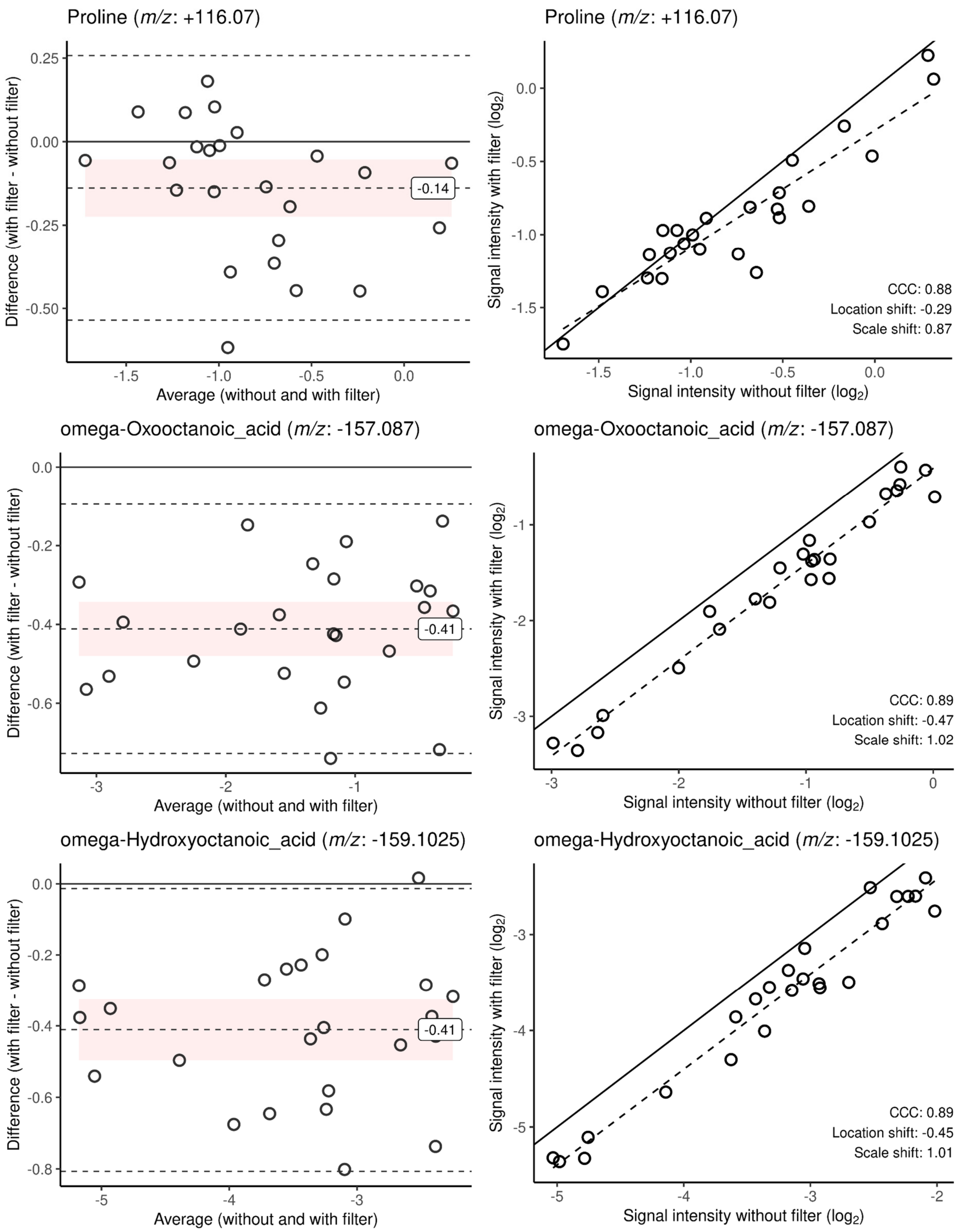
Proline (m/z: +116.07)
Difference (with filter - without filter)
Average (without and with filter)
-0.14
Proline (m/z: +116.07)
Signal intensity with filter (log2)
Signal intensity without filter (log2)
CCC: 0.88
Location shift: -0.29
Scale shift: 0.87
omega-Oxooctanoic_acid (m/z: -157.087)
Difference (with filter - without filter)
Average (without and with filter)
-0.41
omega-Oxooctanoic_acid (m/z: -157.087)
Signal intensity with filter (log2)
Signal intensity without filter (log2)
CCC: 0.89
Location shift: -0.47
Scale shift: 1.02
omega-Hydroxyoctanoic_acid (m/z: -159.1025)
Difference (with filter - without filter)
Average (without and with filter)
-0.41
omega-Hydroxyoctanoic_acid (m/z: -159.1025)
Signal intensity with filter (log2)
Signal intensity without filter (log2)
CCC: 0.89
Location shift: -0.45
Scale shift: 1.01

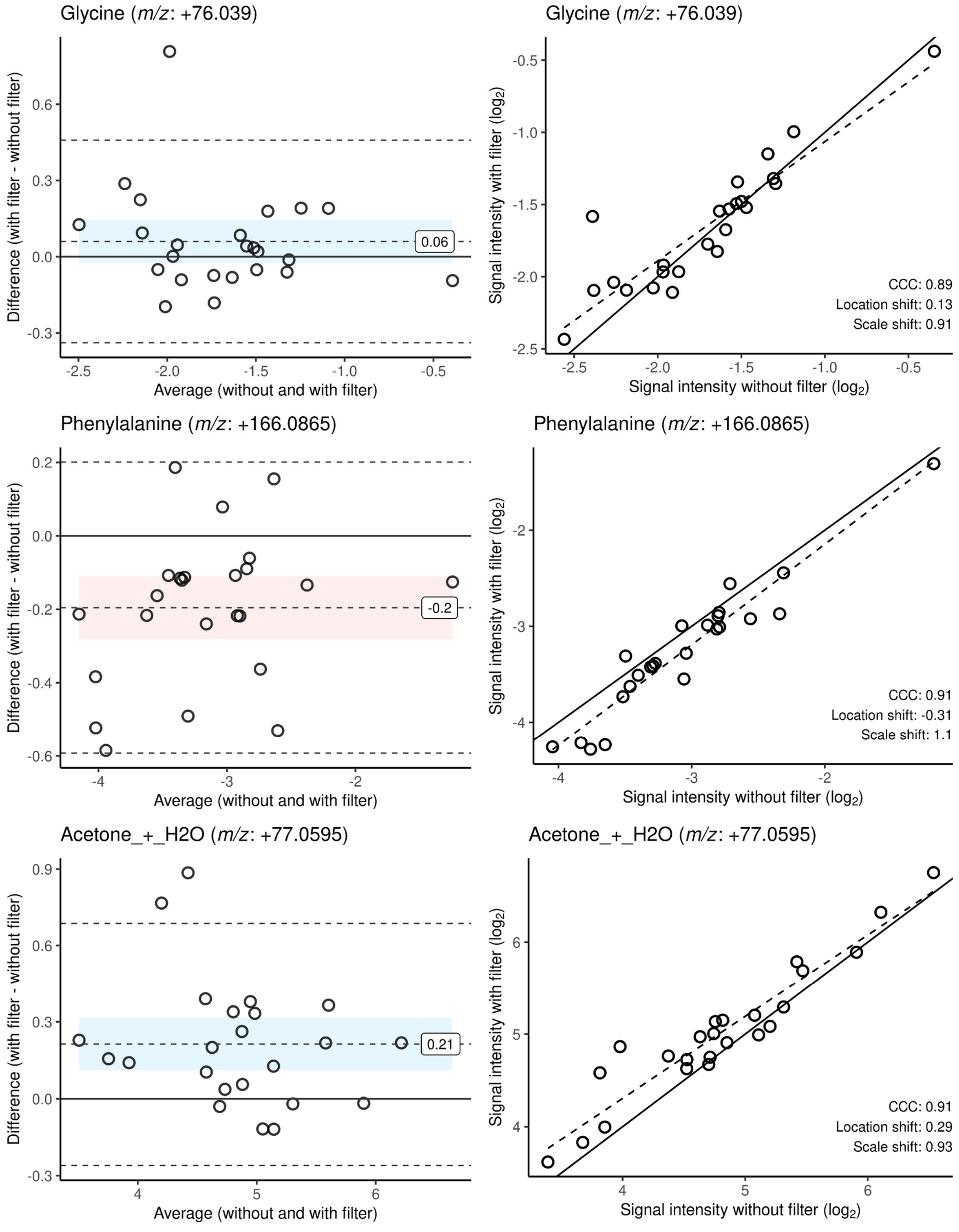
Glycine (m/z: +76.039)
Difference (with filter - without filter)
Average (without and with filter)
0.06
Glycine (m/z: +76.039)
Signal intensity with filter (log2)
Signal intensity without filter (log2)
CCC: 0.89
Location shift: 0.13
Scale shift: 0.91
Phenylalanine (m/z: +166.0865)
Difference (with filter - without filter)
Average (without and with filter)
-0.2
Phenylalanine (m/z: +166.0865)
Signal intensity with filter (log2)
Signal intensity without filter (log2)
CCC: 0.91
Location shift: -0.31
Scale shift: 1.1
Acetone_+_H2O (m/z: +77.0595)
Difference (with filter - without filter)
Average (without and with filter)
0.21
Acetone_+_H2O (m/z: +77.0595)
Signal intensity with filter (log2)
Signal intensity without filter (log2)
CCC: 0.91
Location shift: 0.29
Scale shift: 0.93

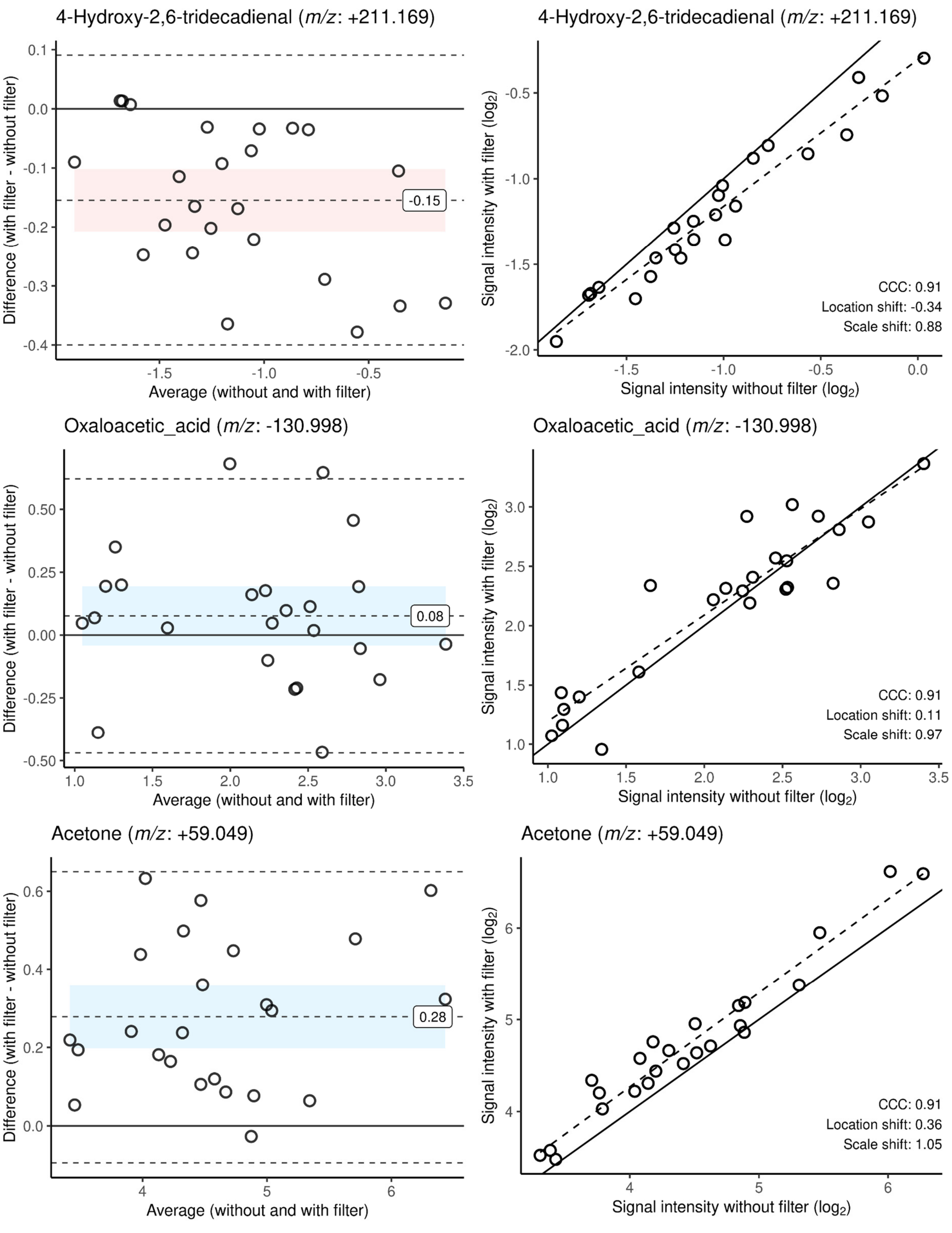

4-Hydroxy-2,6-tridecadienal (m/z: +211.169)
Difference (with filter - without filter)
Average (without and with filter)
-0.15
4-Hydroxy-2,6-tridecadienal (m/z: +211.169)
Signal intensity with filter (log2)
Signal intensity without filter (log2)
CCC: 0.91
Location shift: -0.34
Scale shift: 0.88
Oxaloacetic_acid (m/z: -130.998)
Difference (with filter - without filter)
Average (without and with filter)
0.08
Oxaloacetic_acid (m/z: -130.998)
Signal intensity with filter (log2)
Signal intensity without filter (log2)
CCC: 0.91
Location shift: 0.11
Scale shift: 0.97
Acetone (m/z: +59.049)
Difference (with filter - without filter)
Average (without and with filter)
0.28
Acetone (m/z: +59.049)
Signal intensity with filter (log2)
Signal intensity without filter (log2)
CCC: 0.91
Location shift: 0.36
Scale shift: 1.05

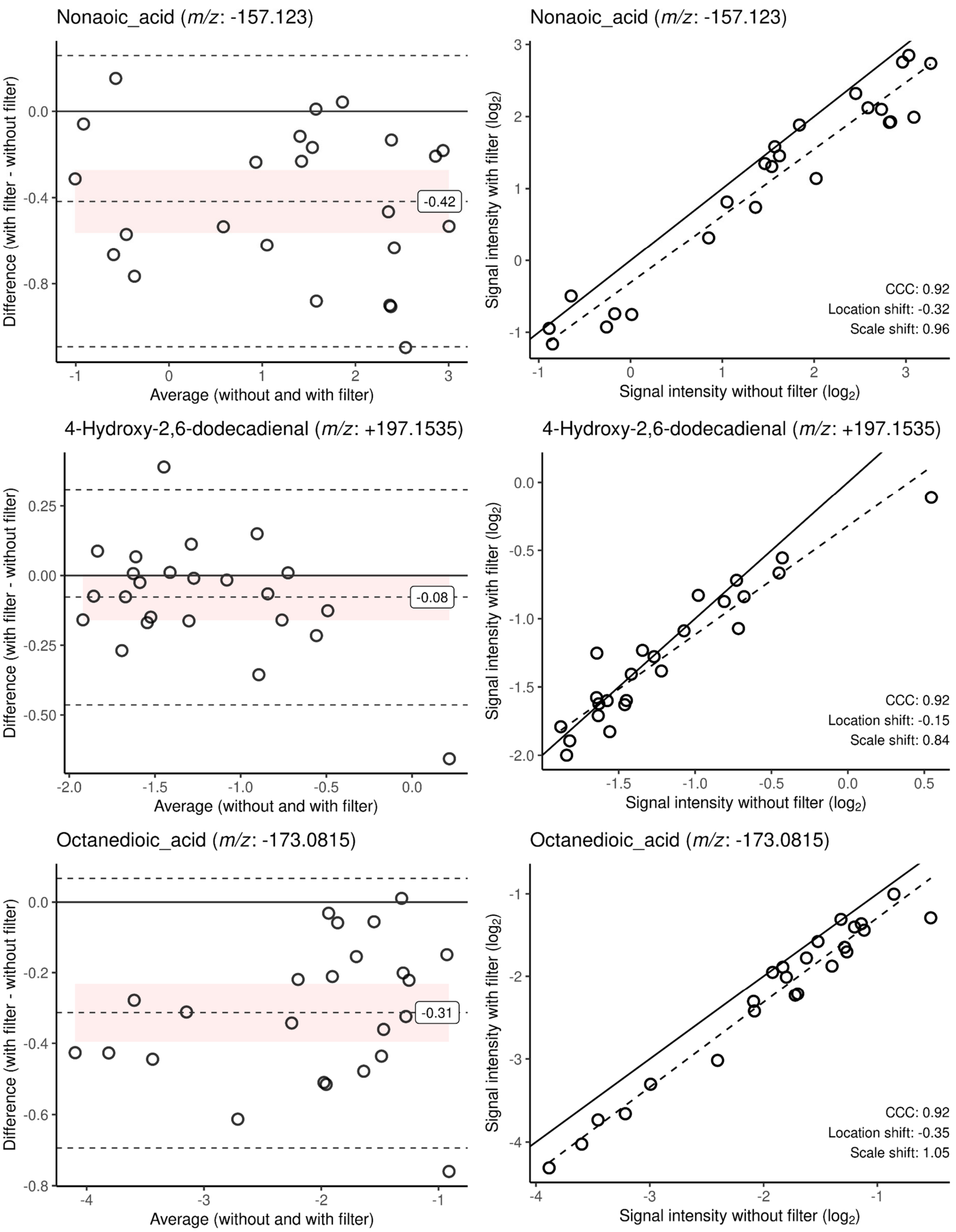
Nonaoic_acid (m/z: -157.123)
Difference (with filter - without filter)
Average (without and with filter)
-0.42
Nonaoic_acid (m/z: -157.123)
Signal intensity with filter (log2)
Signal intensity without filter (log2)
CCC: 0.92
Location shift: -0.32
Scale shift: 0.96
4-Hydroxy-2,6-dodecadienal (m/z: +197.1535)
Difference (with filter - without filter)
Average (without and with filter)
-0.08
4-Hydroxy-2,6-dodecadienal (m/z: +197.1535)
Signal intensity with filter (log2)
Signal intensity without filter (log2)
CCC: 0.92
Location shift: -0.15
Scale shift: 0.84
Octanedioic_acid (m/z: -173.0815)
Difference (with filter - without filter)
Average (without and with filter)
-0.31
Octanedioic_acid (m/z: -173.0815)
Signal intensity with filter (log2)
Signal intensity without filter (log2)
CCC: 0.92
Location shift: -0.35
Scale shift: 1.05

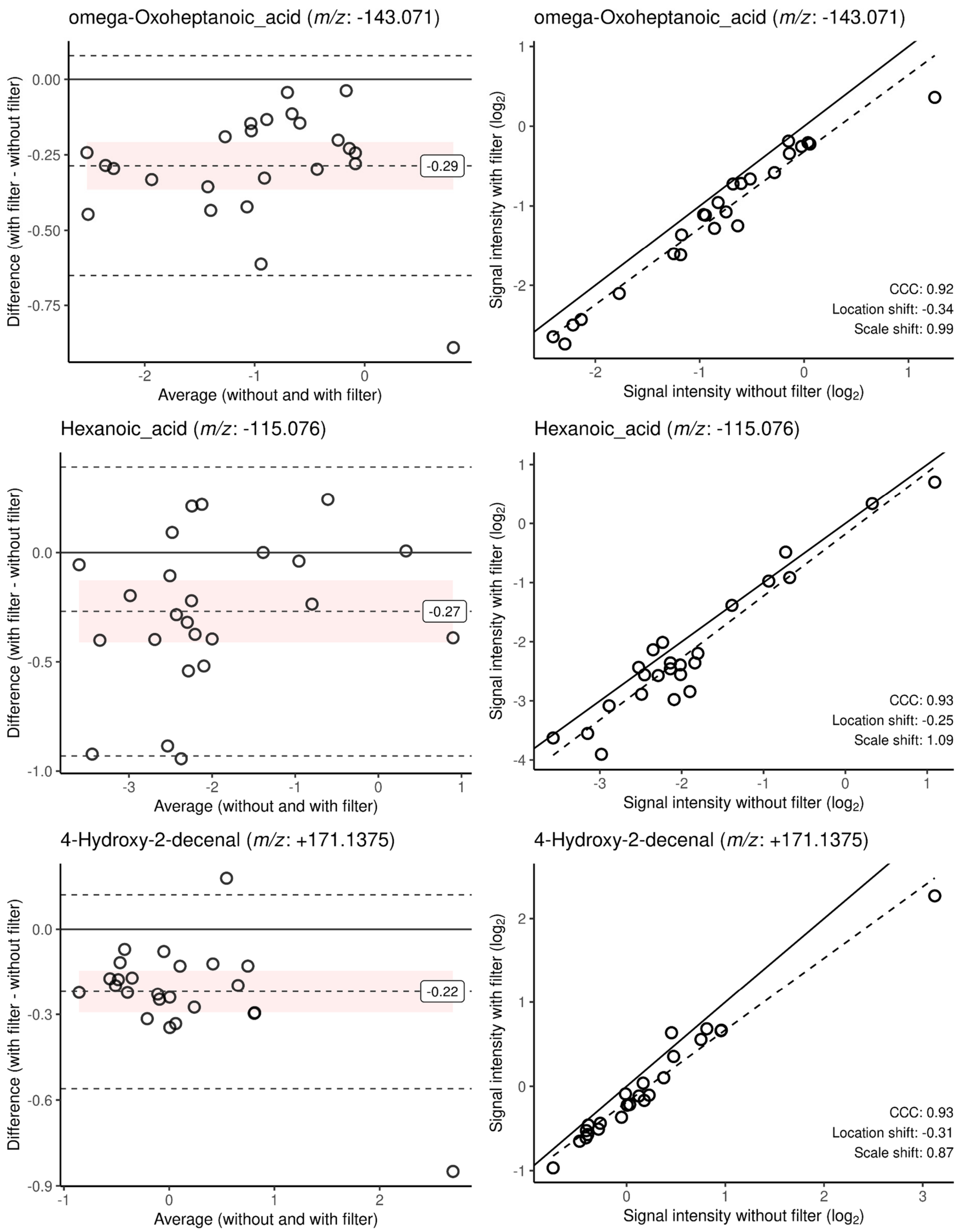

omega-Oxoheptanoic_acid (m/z: -143.071)
Difference (with filter - without filter)
Average (without and with filter)
-0.29
omega-Oxoheptanoic_acid (m/z: -143.071)
Signal intensity with filter (log2)
Signal intensity without filter (log2)
CCC: 0.92
Location shift: -0.34
Scale shift: 0.99
Hexanoic_acid (m/z: -115.076)
Difference (with filter - without filter)
Average (without and with filter)
-0.27
Hexanoic_acid (m/z: -115.076)
Signal intensity with filter (log2)
Signal intensity without filter (log2)
CCC: 0.93
Location shift: -0.25
Scale shift: 1.09
4-Hydroxy-2-decenal (m/z: +171.1375)
Difference (with filter - without filter)
Average (without and with filter)
-0.22
4-Hydroxy-2-decenal (m/z: +171.1375)
Signal intensity with filter (log2)
Signal intensity without filter (log2)
CCC: 0.93
Location shift: -0.31
Scale shift: 0.87

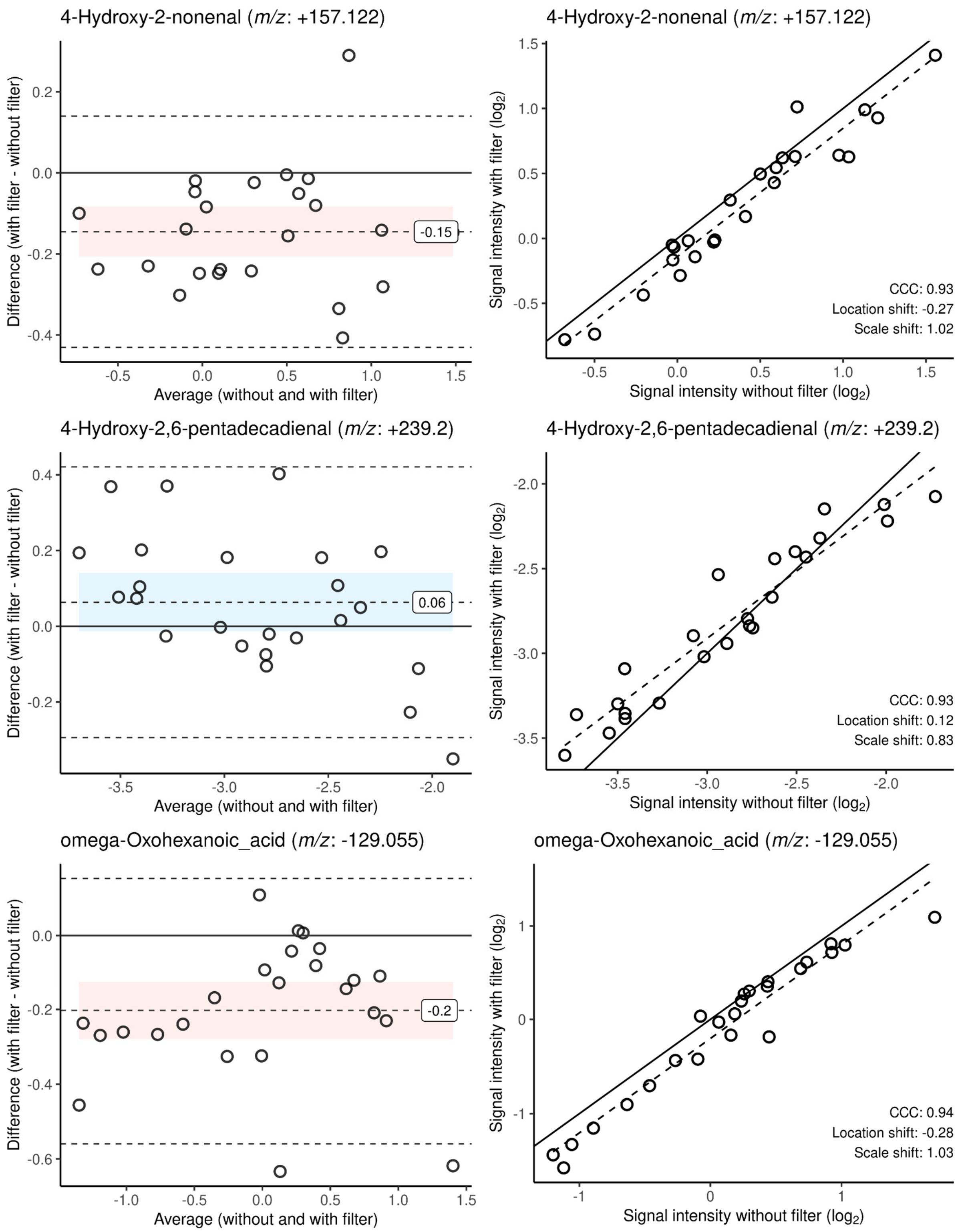
4-Hydroxy-2-nonenal (m/z: +157.122)
Difference (with filter - without filter)
Average (without and with filter)
-0.15
4-Hydroxy-2-nonenal (m/z: +157.122)
Signal intensity with filter (log2)
Signal intensity without filter (log2)
CCC: 0.93
Location shift: -0.27
Scale shift: 1.02
4-Hydroxy-2,6-pentadecadienal (m/z: +239.2)
Difference (with filter - without filter)
Average (without and with filter)
0.06
4-Hydroxy-2,6-pentadecadienal (m/z: +239.2)
Signal intensity with filter (log2)
Signal intensity without filter (log2)
CCC: 0.93
Location shift: 0.12
Scale shift: 0.83
omega-Oxohexanoic_acid (m/z: -129.055)
Difference (with filter - without filter)
Average (without and with filter)
-0.2
omega-Oxohexanoic_acid (m/z: -129.055)
Signal intensity with filter (log2)
Signal intensity without filter (log2)
CCC: 0.94
Location shift: -0.28
Scale shift: 1.03

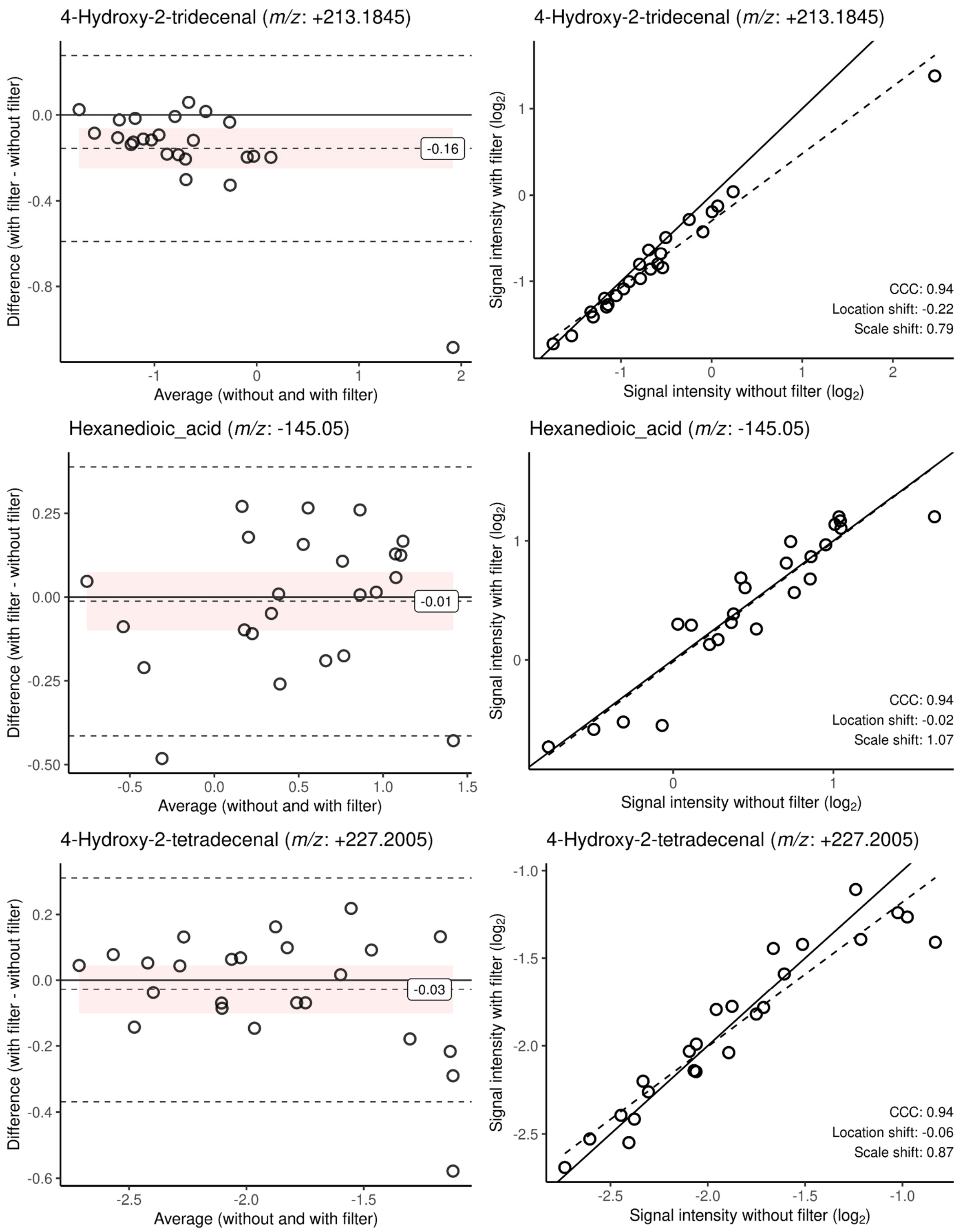

4-Hydroxy-2-tridecenal (m/z: +213.1845)
Difference (with filter - without filter)
Average (without and with filter)
-0.16
4-Hydroxy-2-tridecenal (m/z: +213.1845)
Signal intensity with filter (log2)
Signal intensity without filter (log2)
CCC: 0.94
Location shift: -0.22
Scale shift: 0.79
Hexanedioic_acid (m/z: -145.05)
Difference (with filter - without filter)
Average (without and with filter)
-0.01
Hexanedioic_acid (m/z: -145.05)
Signal intensity with filter (log2)
Signal intensity without filter (log2)
CCC: 0.94
Location shift: -0.02
Scale shift: 1.07
4-Hydroxy-2-tetradecenal (m/z: +227.2005)
Difference (with filter - without filter)
Average (without and with filter)
-0.03
4-Hydroxy-2-tetradecenal (m/z: +227.2005)
Signal intensity with filter (log2)
Signal intensity without filter (log2)
CCC: 0.94
Location shift: -0.06
Scale shift: 0.87

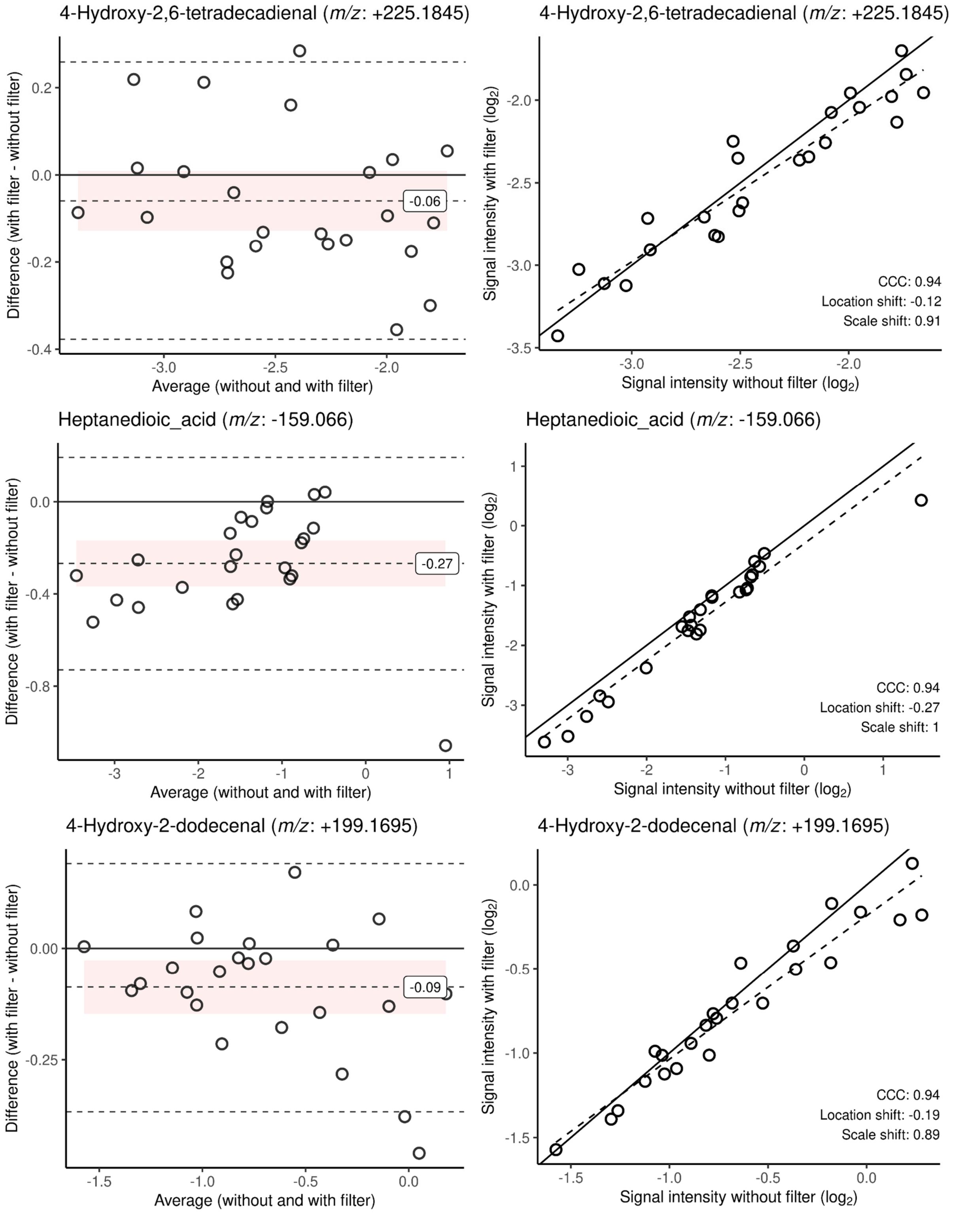
4-Hydroxy-2,6-tetradecadienal (m/z: +225.1845)
Difference (with filter - without filter)
Average (without and with filter)
-0.06
4-Hydroxy-2,6-tetradecadienal (m/z: +225.1845)
Signal intensity with filter (log2)
Signal intensity without filter (log2)
CCC: 0.94
Location shift: -0.12
Scale shift: 0.91
Heptanedioic_acid (m/z: -159.066)
Difference (with filter - without filter)
Average (without and with filter)
-0.27
Heptanedioic_acid (m/z: -159.066)
Signal intensity with filter (log2)
Signal intensity without filter (log2)
CCC: 0.94
Location shift: -0.27
Scale shift: 1
4-Hydroxy-2-dodecenal (m/z: +199.1695)
Difference (with filter - without filter)
Average (without and with filter)
-0.09
4-Hydroxy-2-dodecenal (m/z: +199.1695)
Signal intensity with filter (log2)
Signal intensity without filter (log2)
CCC: 0.94
Location shift: -0.19
Scale shift: 0.89

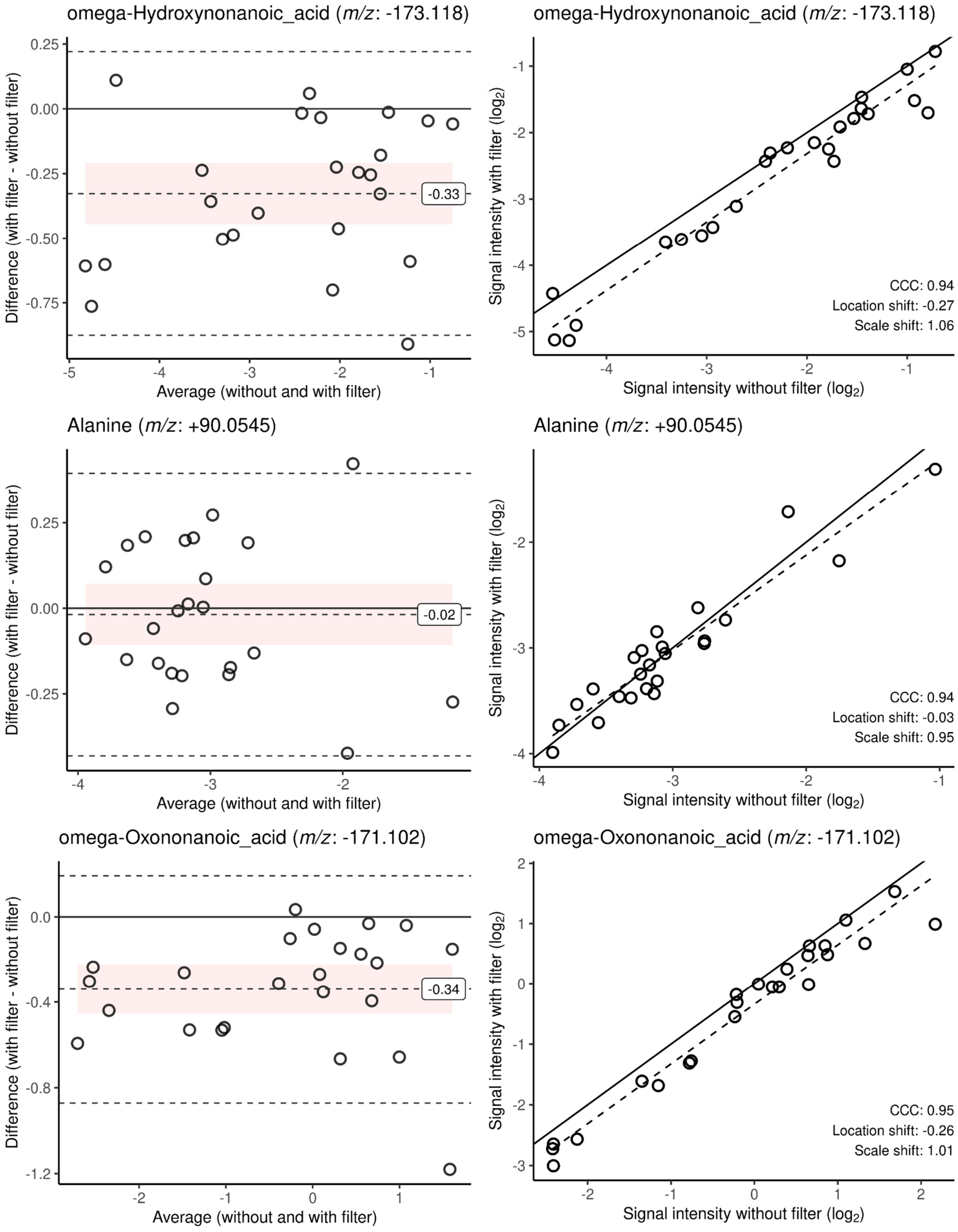
omega-Hydroxynonanoic_acid (m/z: -173.118)
Difference (with filter - without filter)
Average (without and with filter)
-0.33
omega-Hydroxynonanoic_acid (m/z: -173.118)
Signal intensity with filter (log2)
Signal intensity without filter (log2)
CCC: 0.94
Location shift: -0.27
Scale shift: 1.06
Alanine (m/z: +90.0545)
Difference (with filter - without filter)
Average (without and with filter)
-0.02
Alanine (m/z: +90.0545)
Signal intensity with filter (log2)
Signal intensity without filter (log2)
CCC: 0.94
Location shift: -0.03
Scale shift: 0.95
omega-Oxononanoic_acid (m/z: -171.102)
Difference (with filter - without filter)
Average (without and with filter)
-0.34
omega-Oxononanoic_acid (m/z: -171.102)
Signal intensity with filter (log2)
Signal intensity without filter (log2)
CCC: 0.95
Location shift: -0.26
Scale shift: 1.01

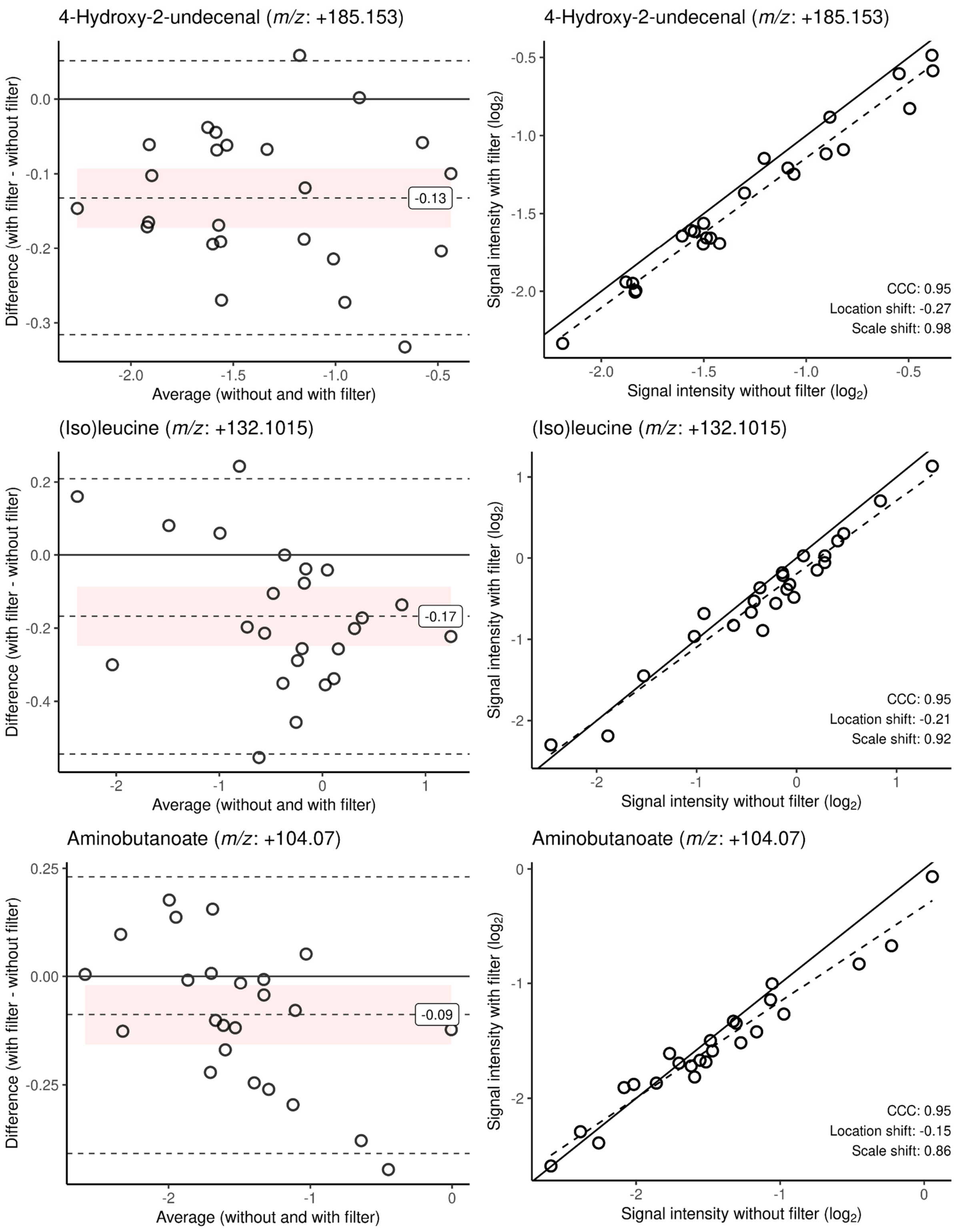
4-Hydroxy-2-undecenal (m/z: +185.153)
Difference (with filter - without filter)
Average (without and with filter)
-0.13
4-Hydroxy-2-undecenal (m/z: +185.153)
Signal intensity with filter (log2)
Signal intensity without filter (log2)
CCC: 0.95
Location shift: -0.27
Scale shift: 0.98
(Iso)leucine (m/z: +132.1015)
Difference (with filter - without filter)
Average (without and with filter)
-0.17
(Iso)leucine (m/z: +132.1015)
Signal intensity with filter (log2)
Signal intensity without filter (log2)
CCC: 0.95
Location shift: -0.21
Scale shift: 0.92
Aminobutanoate (m/z: +104.07)
Difference (with filter - without filter)
Average (without and with filter)
-0.09
Aminobutanoate (m/z: +104.07)
Signal intensity with filter (log2)
Signal intensity without filter (log2)
CCC: 0.95
Location shift: -0.15
Scale shift: 0.86

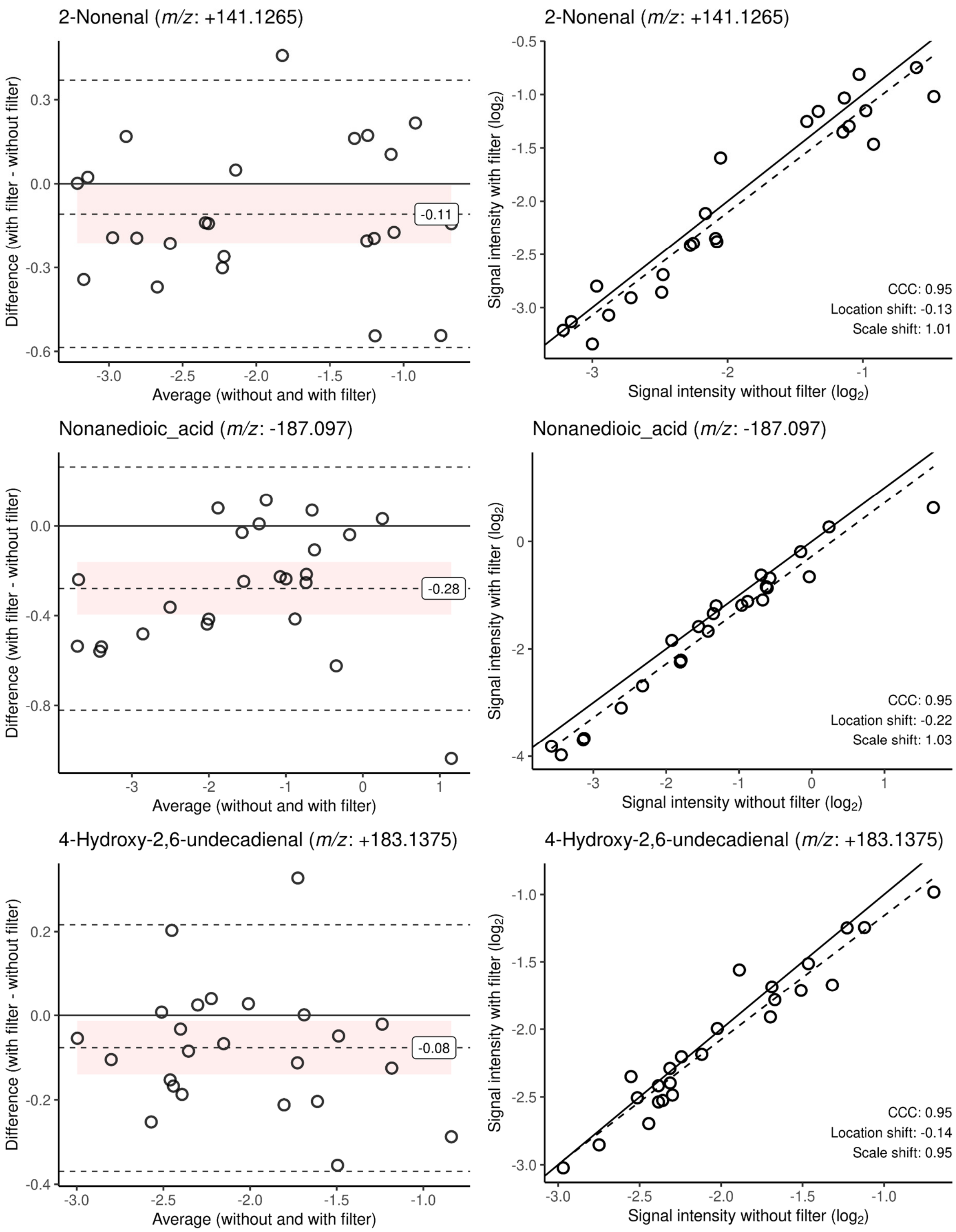
2-Nonenal (m/z: +141.1265)
Difference (with filter - without filter)
Average (without and with filter)
-0.11
2-Nonenal (m/z: +141.1265)
Signal intensity with filter (log2)
Signal intensity without filter (log2)
CCC: 0.95
Location shift: -0.13
Scale shift: 1.01
Nonanedioic_acid (m/z: -187.097)
Difference (with filter - without filter)
Average (without and with filter)
-0.28
Nonanedioic_acid (m/z: -187.097)
Signal intensity with filter (log2)
Signal intensity without filter (log2)
CCC: 0.95
Location shift: -0.22
Scale shift: 1.03
4-Hydroxy-2,6-undecadienal (m/z: +183.1375)
Difference (with filter - without filter)
Average (without and with filter)
-0.08
4-Hydroxy-2,6-undecadienal (m/z: +183.1375)
Signal intensity with filter (log2)
Signal intensity without filter (log2)
CCC: 0.95
Location shift: -0.14
Scale shift: 0.95

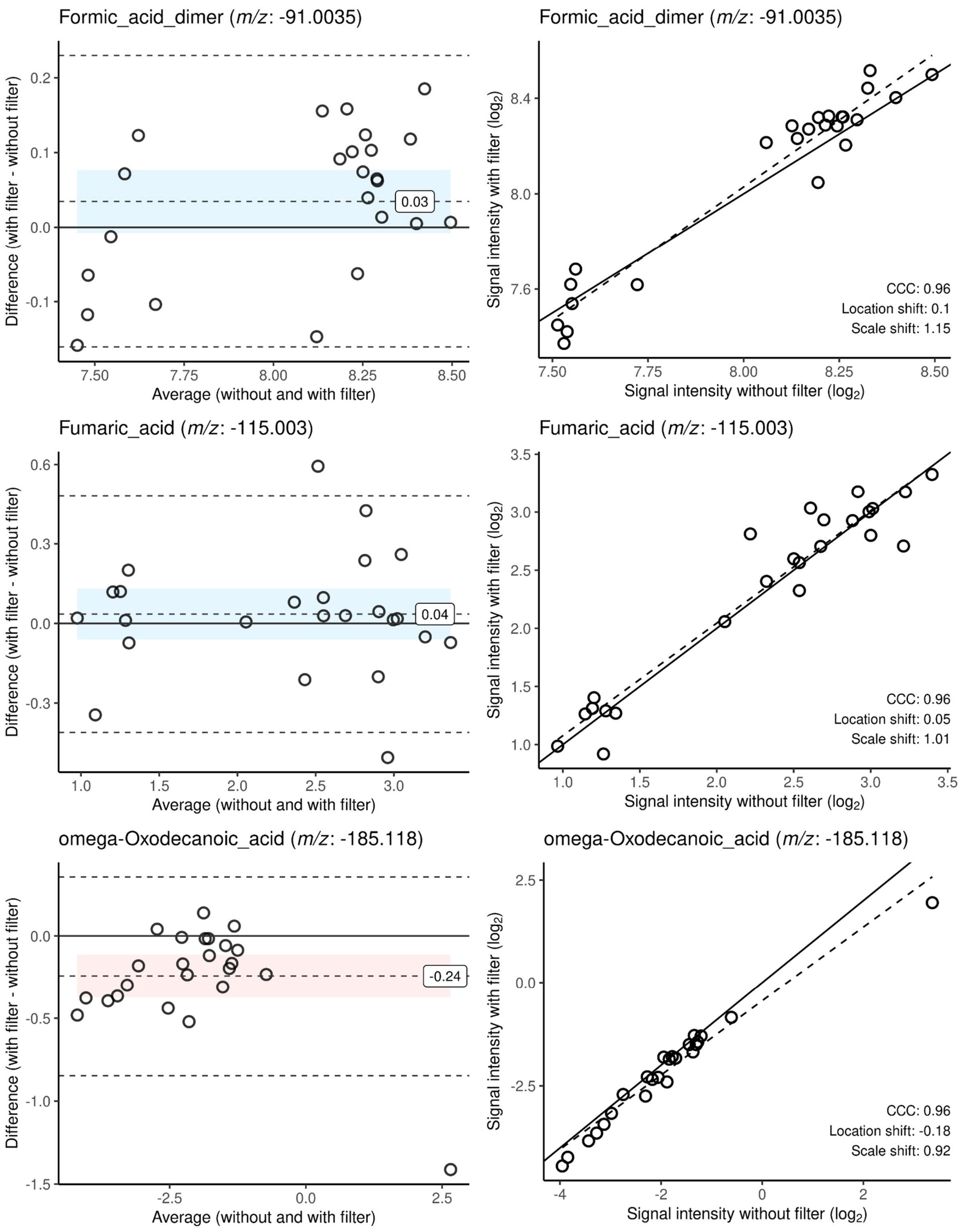
Formic_acid_dimer (m/z: -91.0035)
Difference (with filter - without filter)
Average (without and with filter)
0.03
Formic_acid_dimer (m/z: -91.0035)
Signal intensity with filter (log2)
Signal intensity without filter (log2)
CCC: 0.96
Location shift: 0.1
Scale shift: 1.15
Fumaric_acid (m/z: -115.003)
Difference (with filter - without filter)
Average (without and with filter)
0.04
Fumaric_acid (m/z: -115.003)
Signal intensity with filter (log2)
Signal intensity without filter (log2)
CCC: 0.96
Location shift: 0.05
Scale shift: 1.01
omega-Oxodecanoic_acid (m/z: -185.118)
Difference (with filter - without filter)
Average (without and with filter)
-0.24
omega-Oxodecanoic_acid (m/z: -185.118)
Signal intensity with filter (log2)
Signal intensity without filter (log2)
CCC: 0.96
Location shift: -0.18
Scale shift: 0.92

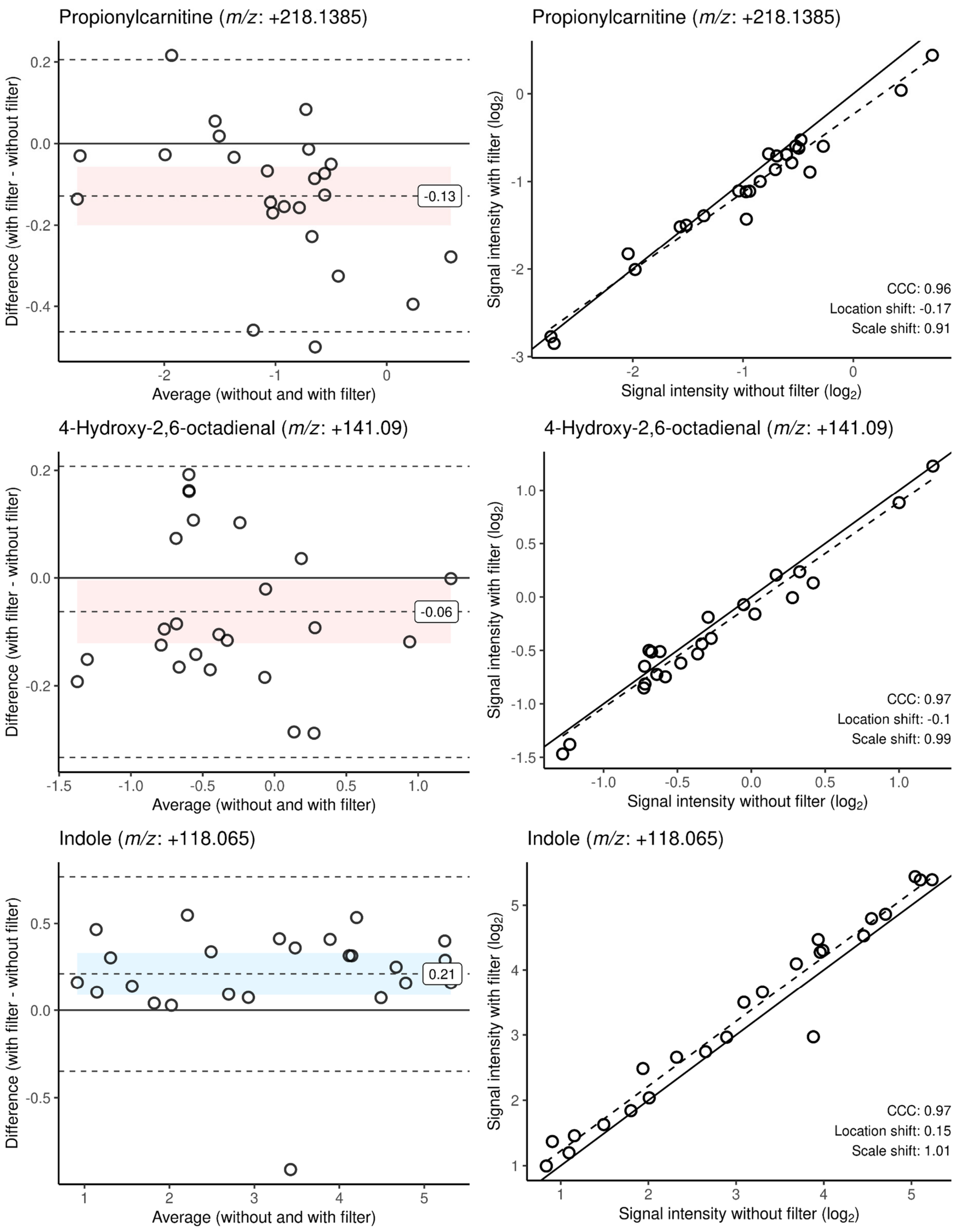
Propionylcarnitine (m/z: +218.1385)
Difference (with filter - without filter)
Average (without and with filter)
-0.13
Propionylcarnitine (m/z: +218.1385)
Signal intensity with filter (log2)
Signal intensity without filter (log2)
CCC: 0.96
Location shift: -0.17
Scale shift: 0.91
4-Hydroxy-2,6-octadienal (m/z: +141.09)
Difference (with filter - without filter)
Average (without and with filter)
-0.06
4-Hydroxy-2,6-octadienal (m/z: +141.09)
Signal intensity with filter (log2)
Signal intensity without filter (log2)
CCC: 0.97
Location shift: -0.1
Scale shift: 0.99
Indole (m/z: +118.065)
Difference (with filter - without filter)
Average (without and with filter)
0.21
Indole (m/z: +118.065)
Signal intensity with filter (log2)
Signal intensity without filter (log2)
CCC: 0.97
Location shift: 0.15
Scale shift: 1.01

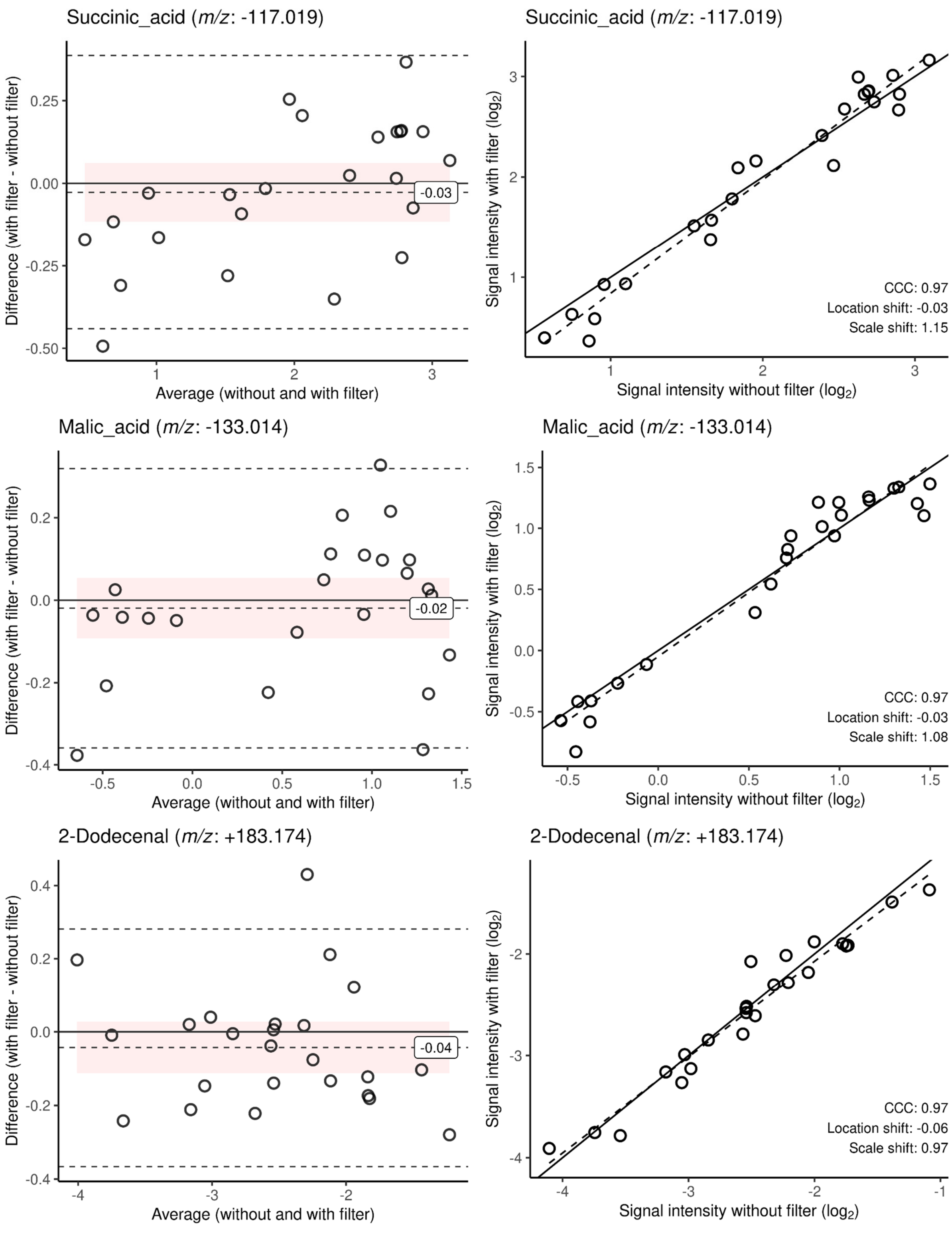
Succinic_acid (m/z: -117.019)
Difference (with filter - without filter)
Average (without and with filter)
-0.03
Succinic_acid (m/z: -117.019)
Signal intensity with filter (log2)
Signal intensity without filter (log2)
CCC: 0.97
Location shift: -0.03
Scale shift: 1.15
Malic_acid (m/z: -133.014)
Difference (with filter - without filter)
Average (without and with filter)
-0.02
Malic_acid (m/z: -133.014)
Signal intensity with filter (log2)
Signal intensity without filter (log2)
CCC: 0.97
Location shift: -0.03
Scale shift: 1.08
2-Dodecenal (m/z: +183.174)
Difference (with filter - without filter)
Average (without and with filter)
-0.04
2-Dodecenal (m/z: +183.174)
Signal intensity with filter (log2)
Signal intensity without filter (log2)
CCC: 0.97
Location shift: -0.06
Scale shift: 0.97

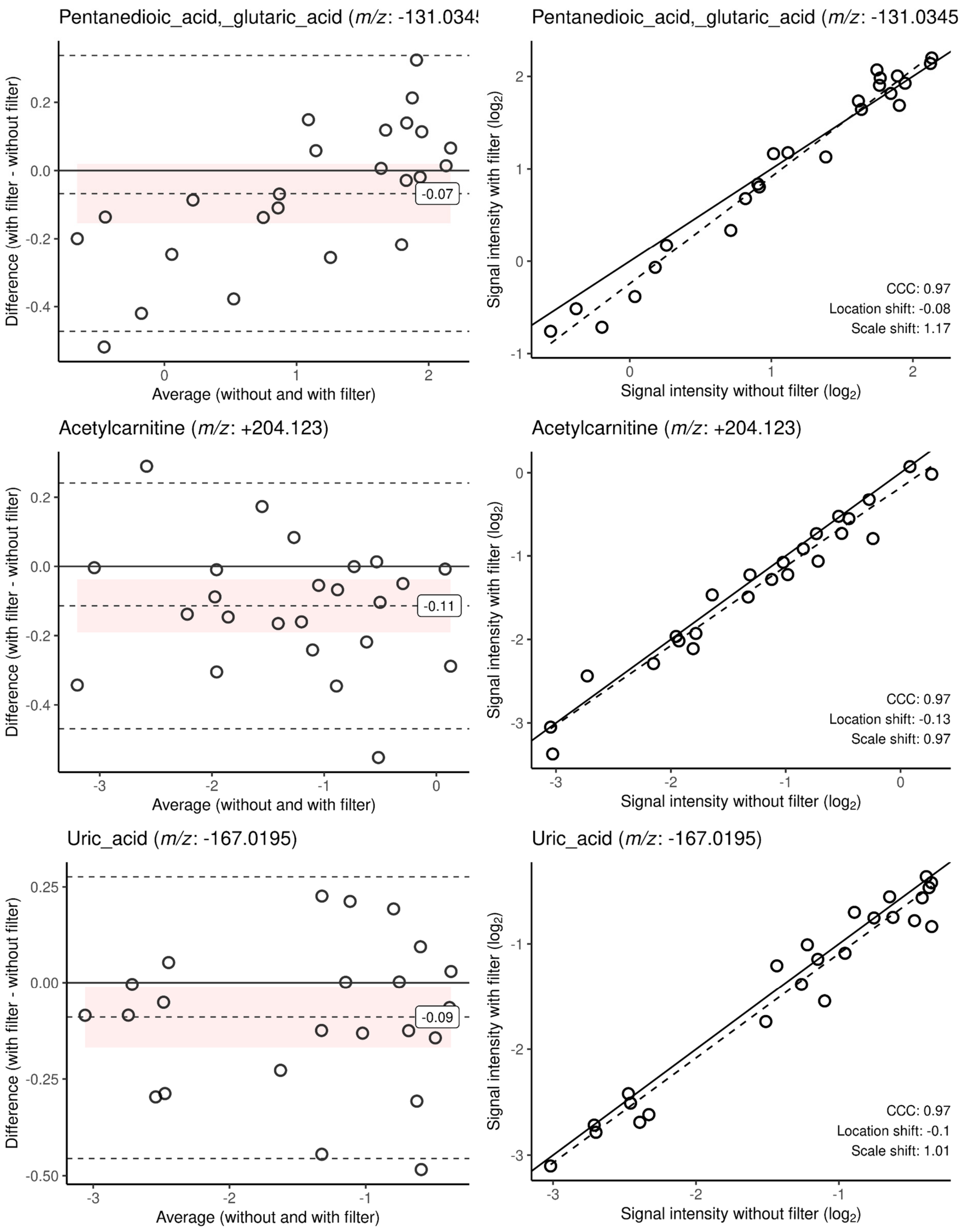

Pentanedioic_acid,_glutaric_acid (m/z: -131.034
Difference (with filter - without filter)
Average (without and with filter)
-0.07
Pentanedioic_acid,_glutaric_acid (m/z: -131.0345
Signal intensity with filter (log2)
Signal intensity without filter (log2)
CCC: 0.97
Location shift: -0.08
Scale shift: 1.17
Acetylcarnitine (m/z: +204.123)
Difference (with filter - without filter)
Average (without and with filter)
-0.11
Acetylcarnitine (m/z: +204.123)
Signal intensity with filter (log2)
Signal intensity without filter (log2)
CCC: 0.97
Location shift: -0.13
Scale shift: 0.97
Uric_acid (m/z: -167.0195)
Difference (with filter - without filter)
Average (without and with filter)
-0.09
Uric_acid (m/z: -167.0195)
Signal intensity with filter (log2)
Signal intensity without filter (log2)
CCC: 0.97
Location shift: -0.1
Scale shift: 1.01

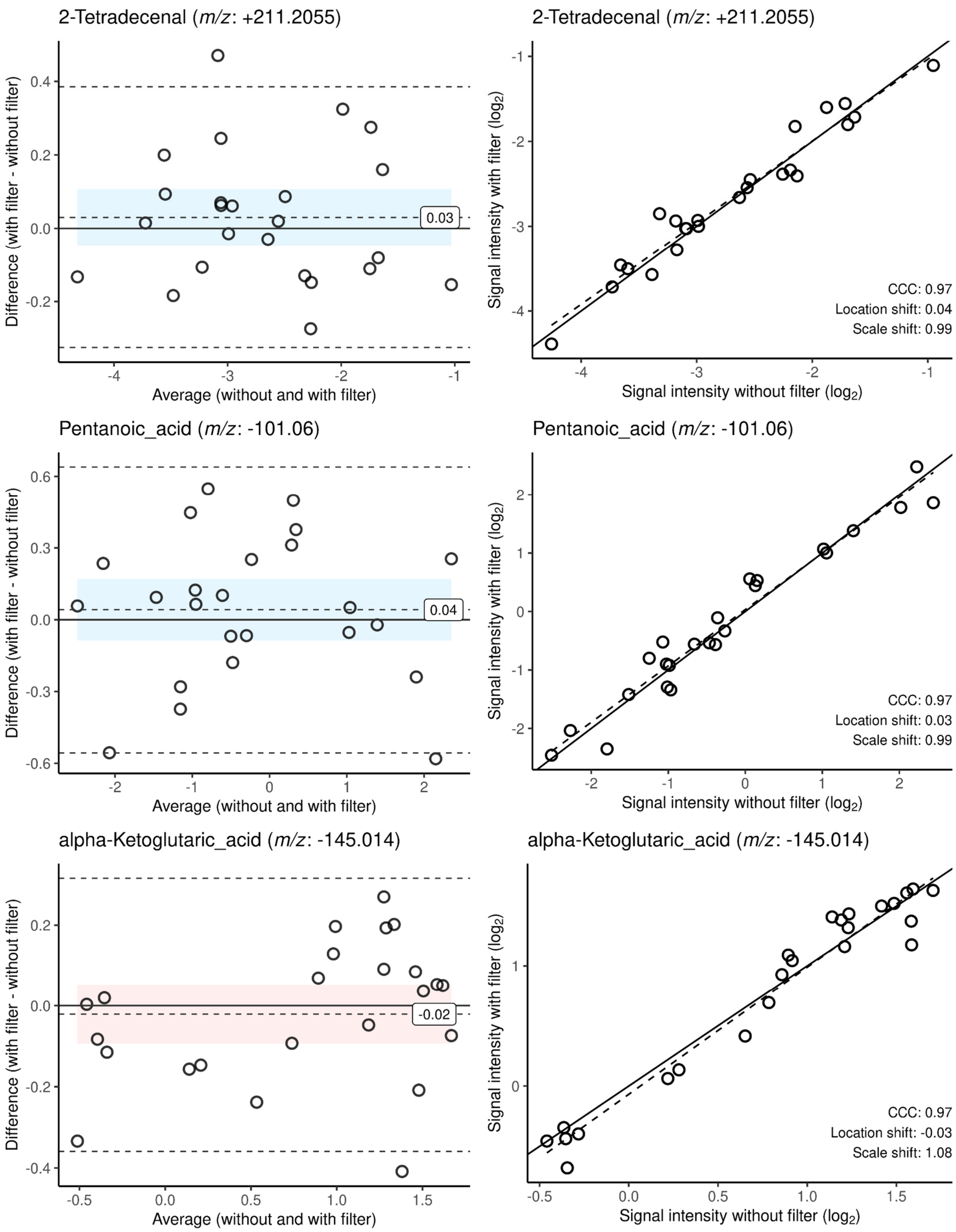
2-Tetradecenal (m/z: +211.2055)
Difference (with filter - without filter)
Average (without and with filter)
0.03
2-Tetradecenal (m/z: +211.2055)
Signal intensity with filter (log2)
Signal intensity without filter (log2)
CCC: 0.97
Location shift: 0.04
Scale shift: 0.99
Pentanoic_acid (m/z: -101.06)
Difference (with filter - without filter)
Average (without and with filter)
0.04
Pentanoic_acid (m/z: -101.06)
Signal intensity with filter (log2)
Signal intensity without filter (log2)
CCC: 0.97
Location shift: 0.03
Scale shift: 0.99
alpha-Ketoglutaric_acid (m/z: -145.014)
Difference (with filter - without filter)
Average (without and with filter)
-0.02
alpha-Ketoglutaric_acid (m/z: -145.014)
Signal intensity with filter (log2)
Signal intensity without filter (log2)
CCC: 0.97
Location shift: -0.03
Scale shift: 1.08

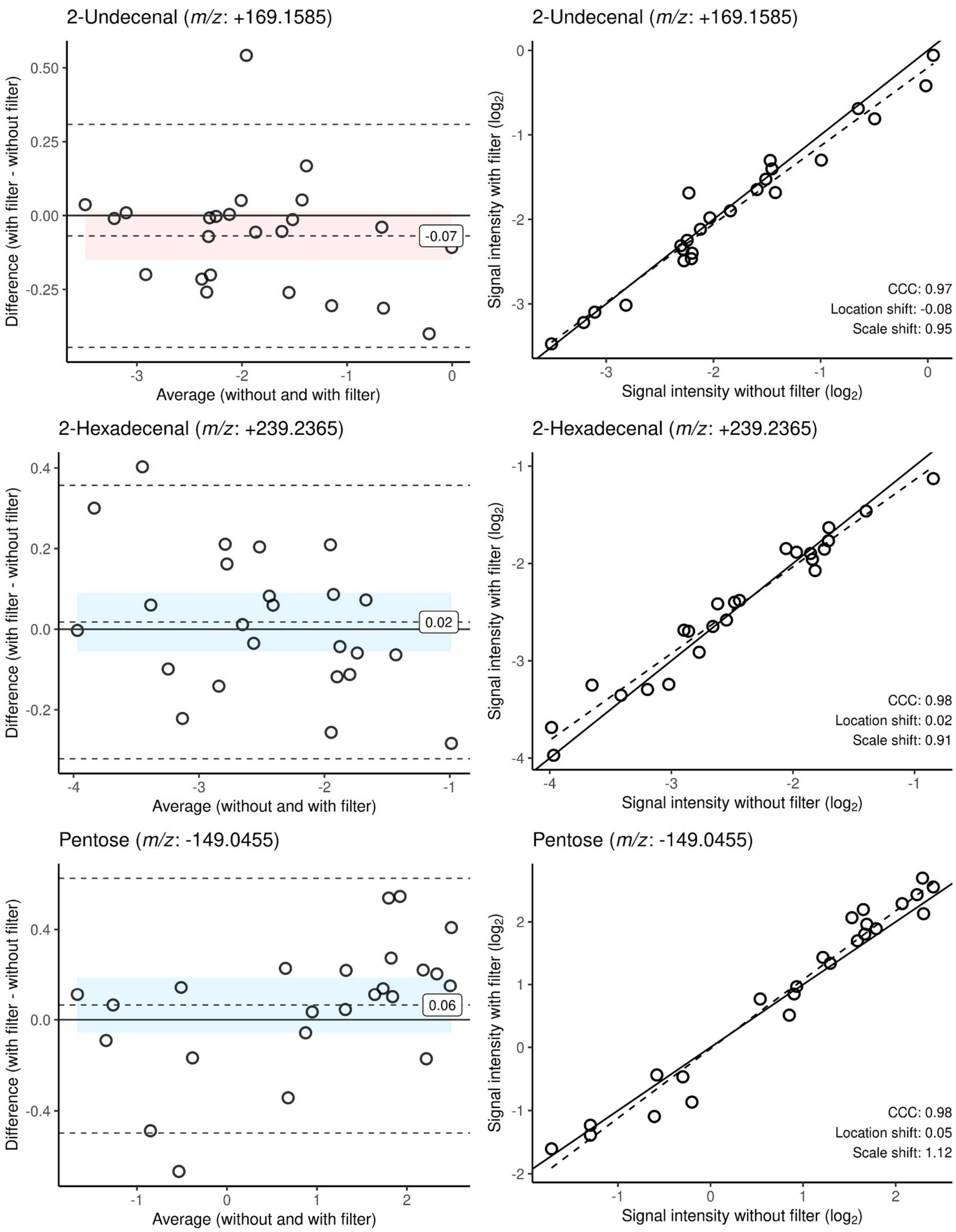
2-Undecenal (m/z: +169.1585)
Difference (with filter - without filter)
Average (without and with filter)
-0.07
2-Undecenal (m/z: +169.1585)
Signal intensity with filter ($\log_2$)
Signal intensity without filter ($\log_2$)
CCC: 0.97
Location shift: -0.08
Scale shift: 0.95
2-Hexadecenal (m/z: +239.2365)
Difference (with filter - without filter)
Average (without and with filter)
0.02
2-Hexadecenal (m/z: +239.2365)
Signal intensity with filter ($\log_2$)
Signal intensity without filter ($\log_2$)
CCC: 0.98
Location shift: 0.02
Scale shift: 0.91
Pentose (m/z: -149.0455)
Difference (with filter - without filter)
Average (without and with filter)
0.06
Pentose (m/z: -149.0455)
Signal intensity with filter ($\log_2$)
Signal intensity without filter ($\log_2$)
CCC: 0.98
Location shift: 0.05
Scale shift: 1.12

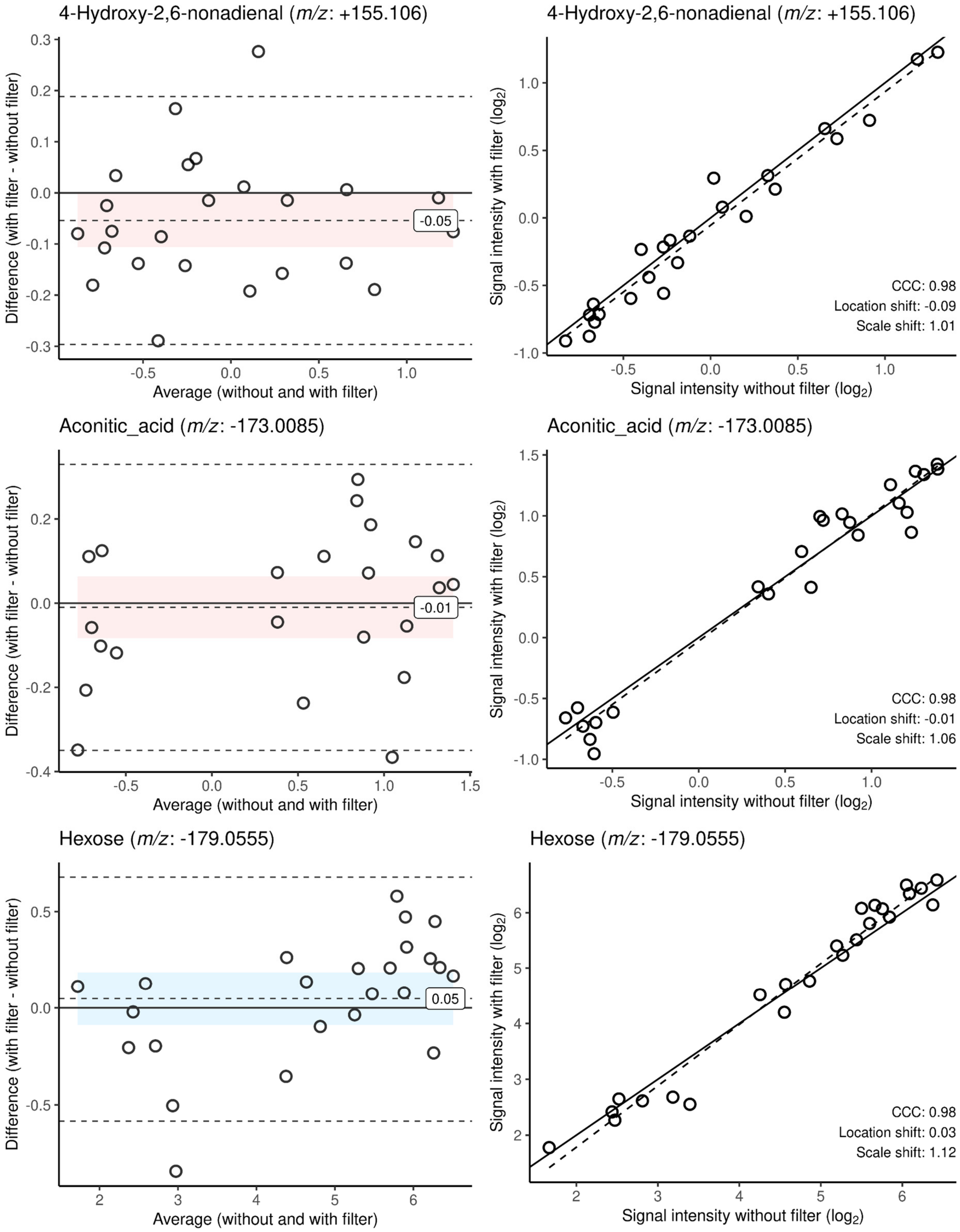

4-Hydroxy-2,6-nonadienal (m/z: +155.106)
Difference (with filter - without filter)
Average (without and with filter)
-0.05
4-Hydroxy-2,6-nonadienal (m/z: +155.106)
Signal intensity with filter (log2)
Signal intensity without filter (log2)
CCC: 0.98
Location shift: -0.09
Scale shift: 1.01
Aconitic_acid (m/z: -173.0085)
Difference (with filter - without filter)
Average (without and with filter)
-0.01
Aconitic_acid (m/z: -173.0085)
Signal intensity with filter (log2)
Signal intensity without filter (log2)
CCC: 0.98
Location shift: -0.01
Scale shift: 1.06
Hexose (m/z: -179.0555)
Difference (with filter - without filter)
Average (without and with filter)
0.05
Hexose (m/z: -179.0555)
Signal intensity with filter (log2)
Signal intensity without filter (log2)
CCC: 0.98
Location shift: 0.03
Scale shift: 1.12

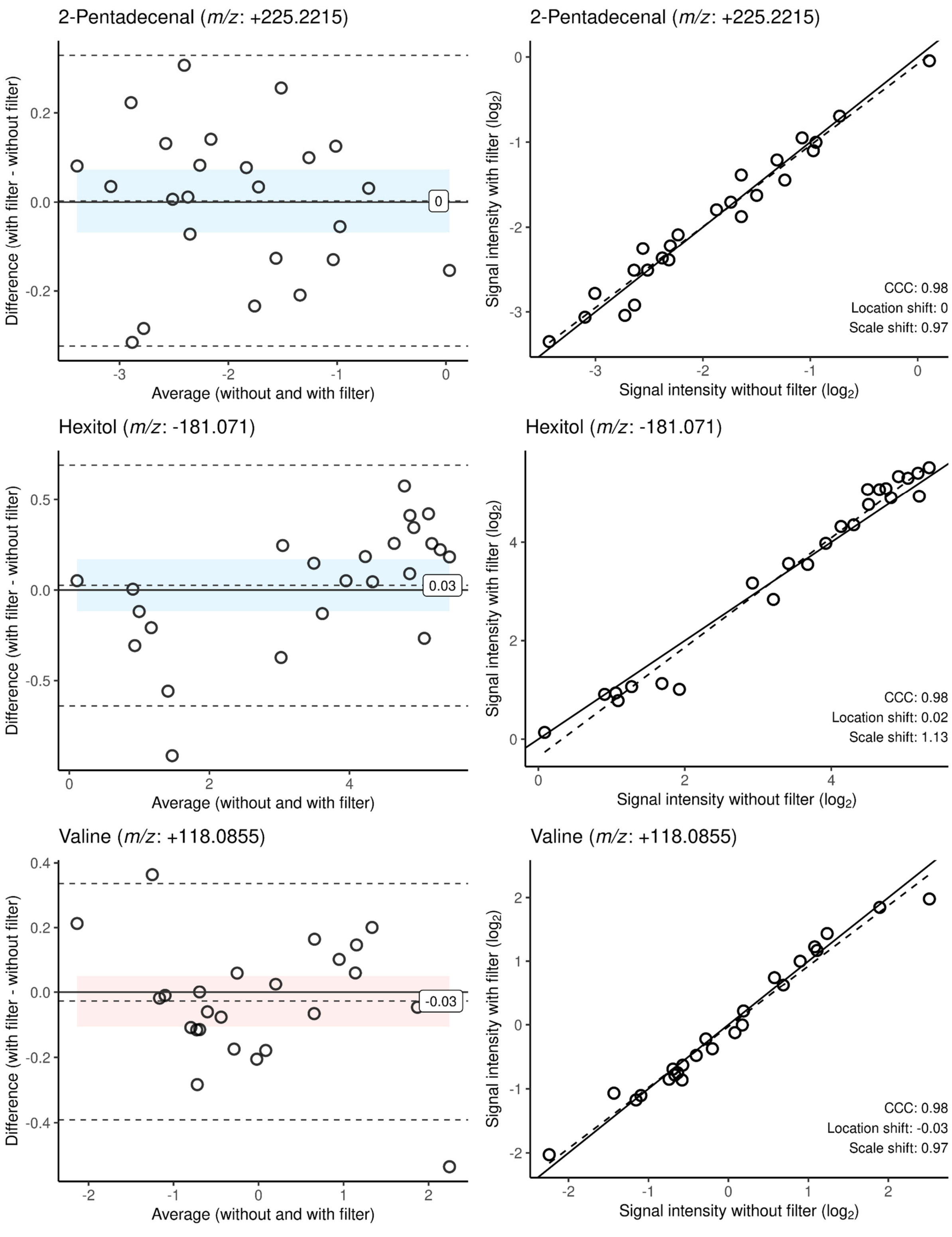
2-Pentadecenal (m/z: +225.2215)
Difference (with filter - without filter)
Average (without and with filter)
0
2-Pentadecenal (m/z: +225.2215)
Signal intensity with filter (log2)
Signal intensity without filter (log2)
CCC: 0.98
Location shift: 0
Scale shift: 0.97
Hexitol (m/z: -181.071)
Difference (with filter - without filter)
Average (without and with filter)
0.03
Hexitol (m/z: -181.071)
Signal intensity with filter (log2)
Signal intensity without filter (log2)
CCC: 0.98
Location shift: 0.02
Scale shift: 1.13
Valine (m/z: +118.0855)
Difference (with filter - without filter)
Average (without and with filter)
-0.03
Valine (m/z: +118.0855)
Signal intensity with filter (log2)
Signal intensity without filter (log2)
CCC: 0.98
Location shift: -0.03
Scale shift: 0.97

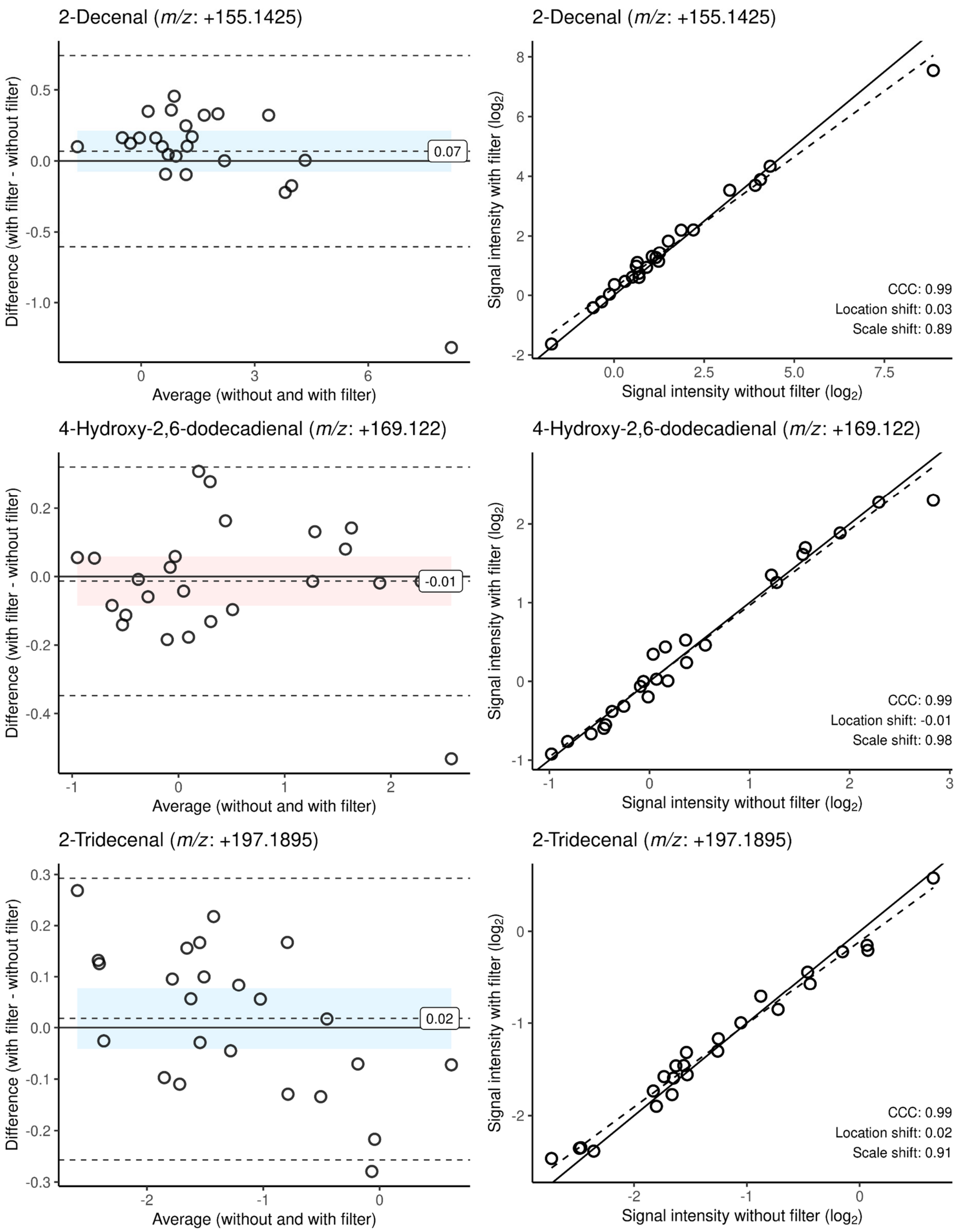
2-Decenal (m/z: +155.1425)
Difference (with filter - without filter)
Average (without and with filter)
0.07
2-Decenal (m/z: +155.1425)
Signal intensity with filter (log2)
Signal intensity without filter (log2)
CCC: 0.99
Location shift: 0.03
Scale shift: 0.89
4-Hydroxy-2,6-dodecadienal (m/z: +169.122)
Difference (with filter - without filter)
Average (without and with filter)
-0.01
4-Hydroxy-2,6-dodecadienal (m/z: +169.122)
Signal intensity with filter (log2)
Signal intensity without filter (log2)
CCC: 0.99
Location shift: -0.01
Scale shift: 0.98
2-Tridecenal (m/z: +197.1895)
Difference (with filter - without filter)
Average (without and with filter)
0.02
2-Tridecenal (m/z: +197.1895)
Signal intensity with filter (log2)
Signal intensity without filter (log2)
CCC: 0.99
Location shift: 0.02
Scale shift: 0.91

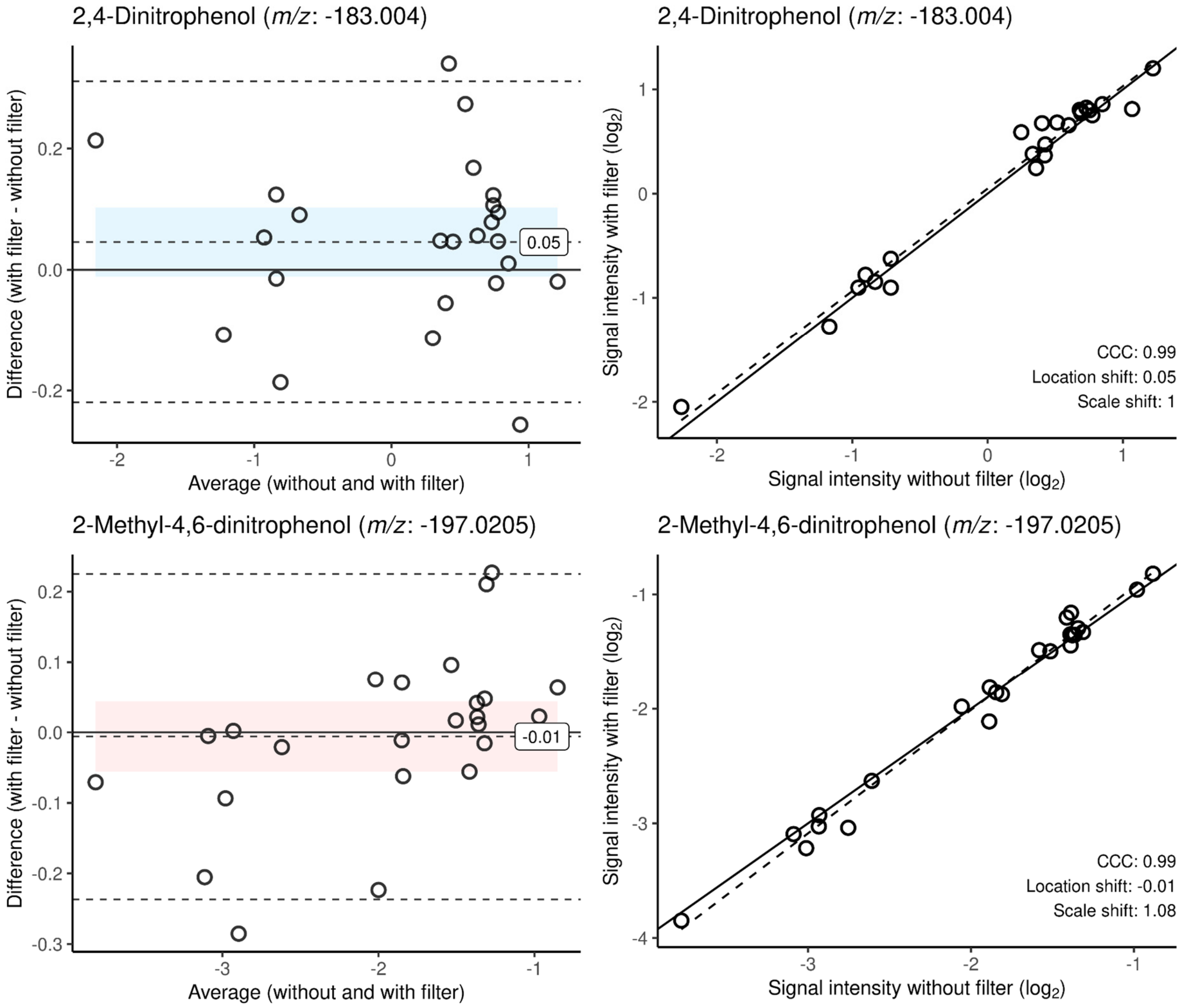

3.2 Figure S2

Bland-Altman and CCC plots for all detected contaminants in the exhaled breath profiles. For explanation of the plots see text of Figure S1.

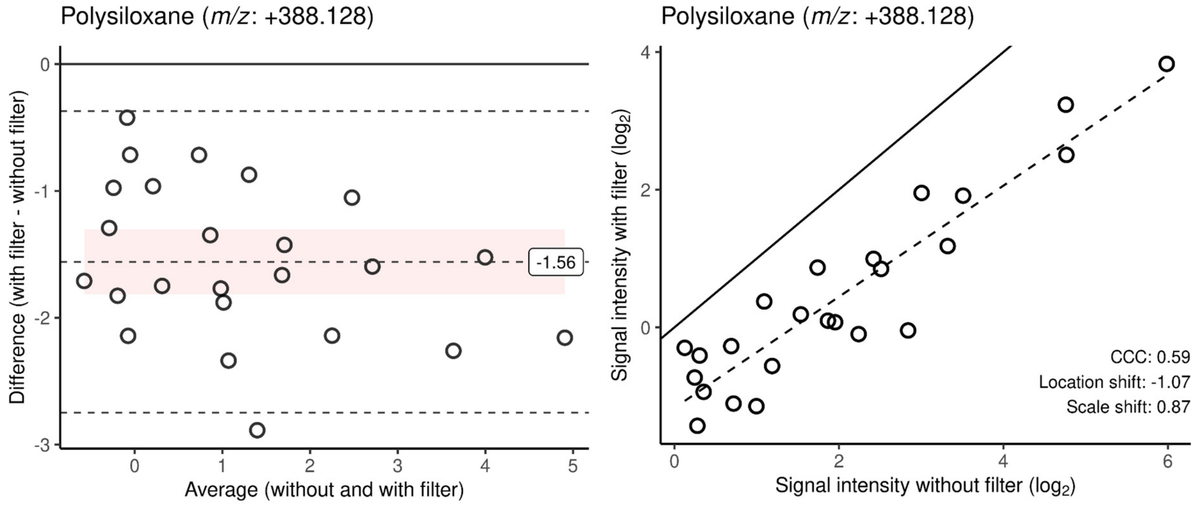

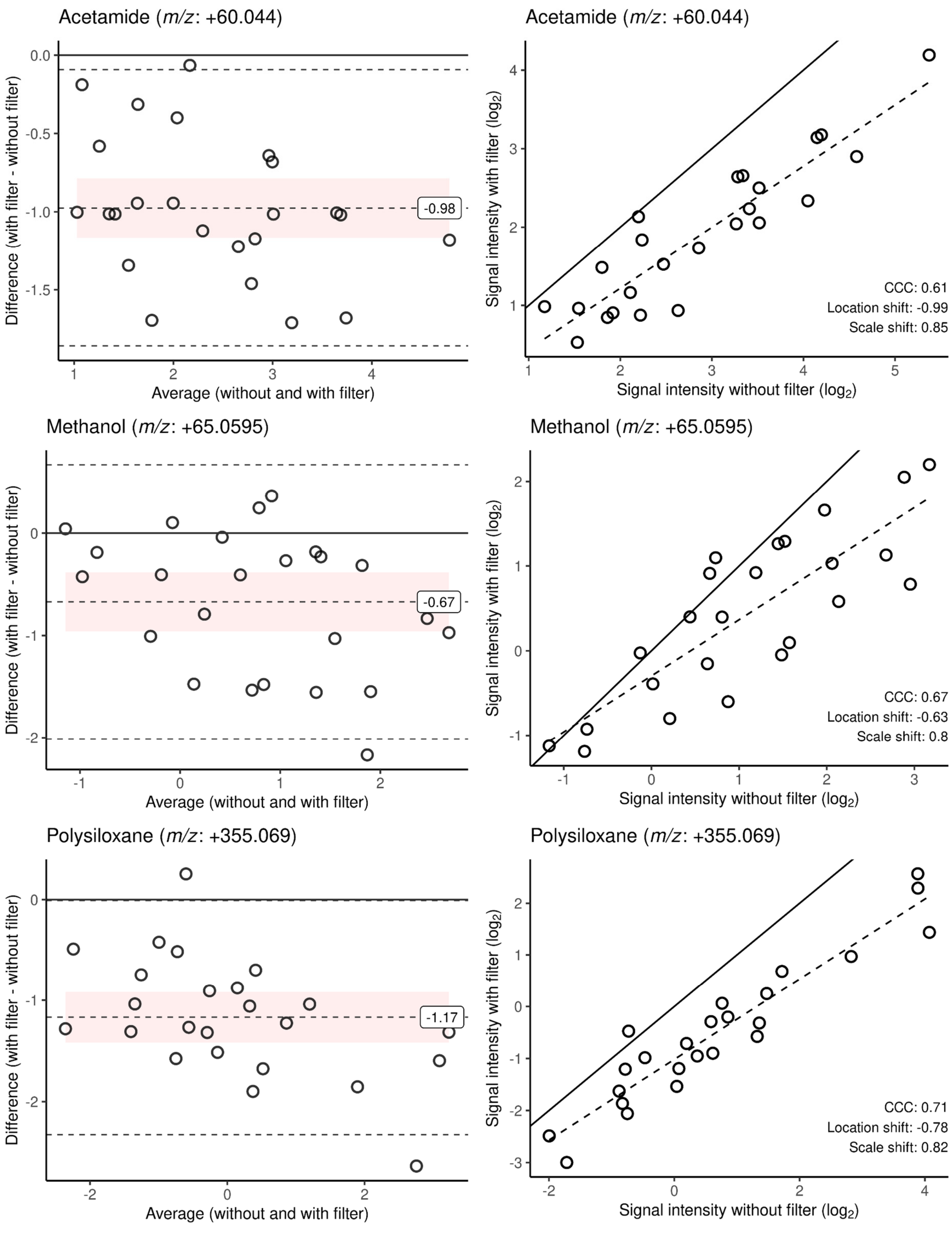

Acetamide (m/z: +60.044)
Difference (with filter - without filter)
Average (without and with filter)
-0.98
Acetamide (m/z: +60.044)
Signal intensity with filter (log2)
Signal intensity without filter (log2)
CCC: 0.61
Location shift: -0.99
Scale shift: 0.85
Methanol (m/z: +65.0595)
Difference (with filter - without filter)
Average (without and with filter)
-0.67
Methanol (m/z: +65.0595)
Signal intensity with filter (log2)
Signal intensity without filter (log2)
CCC: 0.67
Location shift: -0.63
Scale shift: 0.8
Polysiloxane (m/z: +355.069)
Difference (with filter - without filter)
Average (without and with filter)
-1.17
Polysiloxane (m/z: +355.069)
Signal intensity with filter (log2)
Signal intensity without filter (log2)
CCC: 0.71
Location shift: -0.78
Scale shift: 0.82

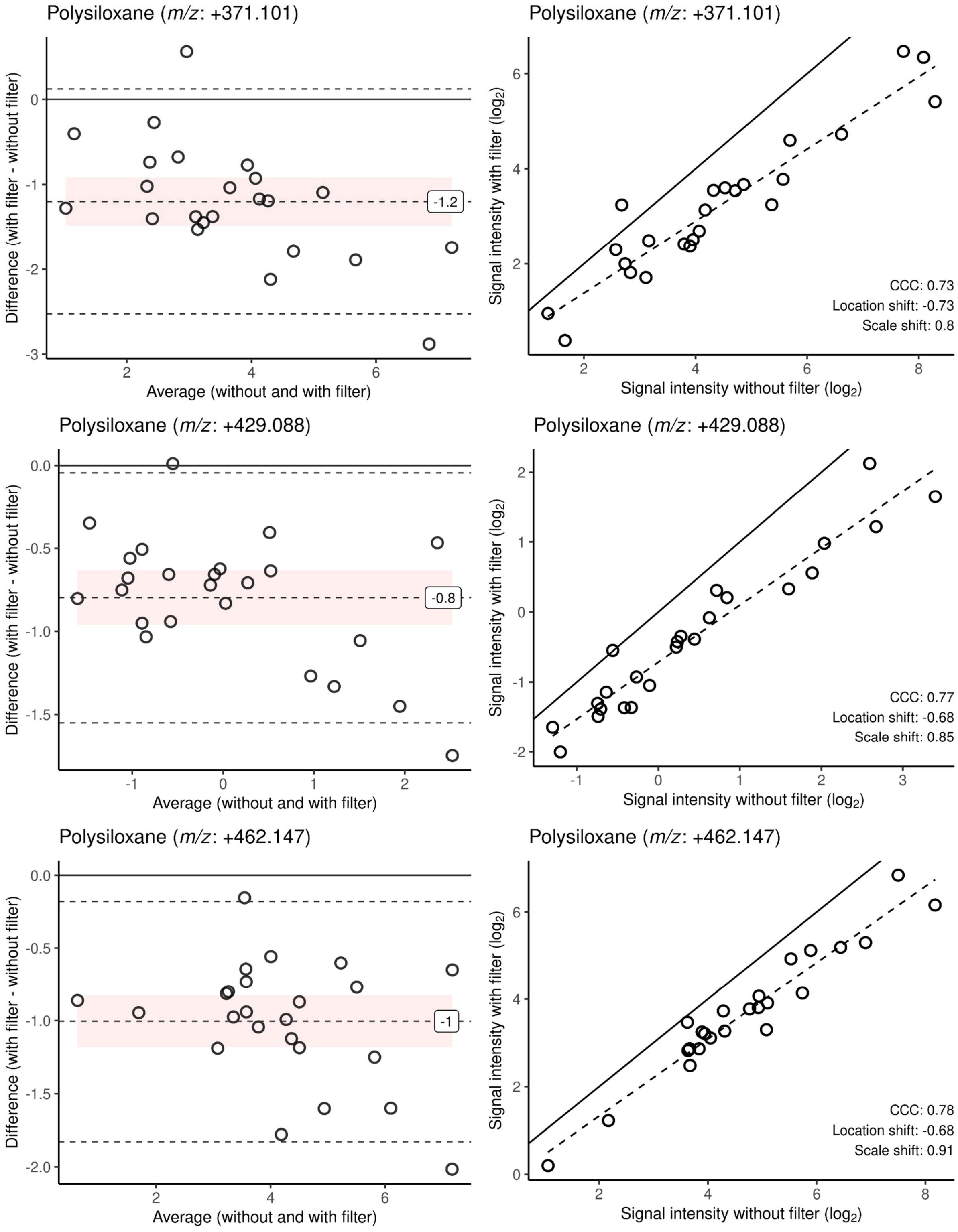
Polysiloxane (m/z: +371.101)
Difference (with filter - without filter)
Average (without and with filter)
-1.2
Polysiloxane (m/z: +371.101)
Signal intensity with filter (log2)
Signal intensity without filter (log2)
CCC: 0.73
Location shift: -0.73
Scale shift: 0.8
Polysiloxane (m/z: +429.088)
Difference (with filter - without filter)
Average (without and with filter)
-0.8
Polysiloxane (m/z: +429.088)
Signal intensity with filter (log2)
Signal intensity without filter (log2)
CCC: 0.77
Location shift: -0.68
Scale shift: 0.85
Polysiloxane (m/z: +462.147)
Difference (with filter - without filter)
Average (without and with filter)
-1
Polysiloxane (m/z: +462.147)
Signal intensity with filter (log2)
Signal intensity without filter (log2)
CCC: 0.78
Location shift: -0.68
Scale shift: 0.91

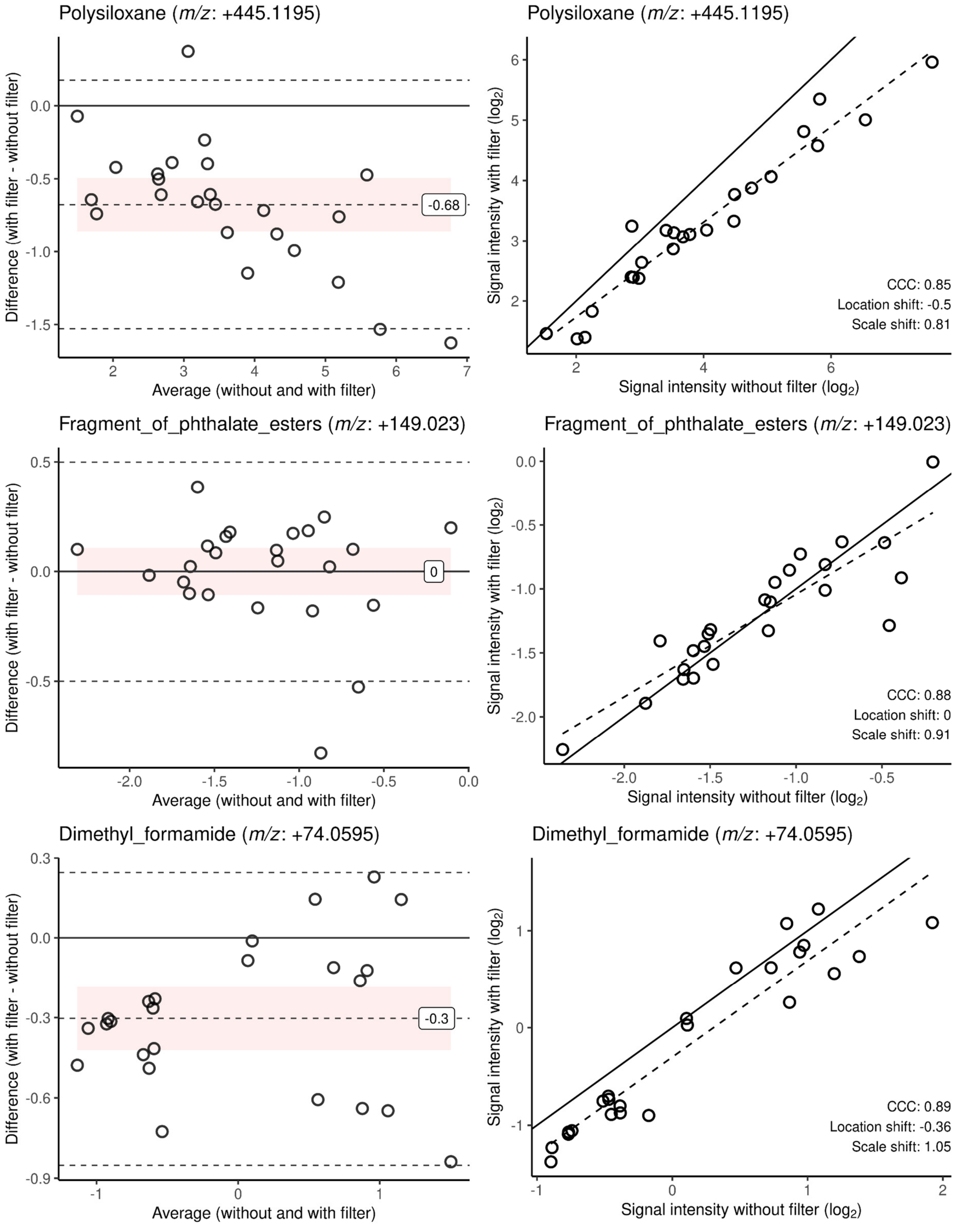

Polysiloxane (m/z: +445.1195)
Difference (with filter - without filter)
Average (without and with filter)
-0.68
Polysiloxane (m/z: +445.1195)
Signal intensity with filter (log2)
Signal intensity without filter (log2)
CCC: 0.85
Location shift: -0.5
Scale shift: 0.81
Fragment_of_phthalate_esters (m/z: +149.023)
Difference (with filter - without filter)
Average (without and with filter)
0
Fragment_of_phthalate_esters (m/z: +149.023)
Signal intensity with filter (log2)
Signal intensity without filter (log2)
CCC: 0.88
Location shift: 0
Scale shift: 0.91
Dimethyl_formamide (m/z: +74.0595)
Difference (with filter - without filter)
Average (without and with filter)
-0.3
Dimethyl_formamide (m/z: +74.0595)
Signal intensity with filter (log2)
Signal intensity without filter (log2)
CCC: 0.89
Location shift: -0.36
Scale shift: 1.05

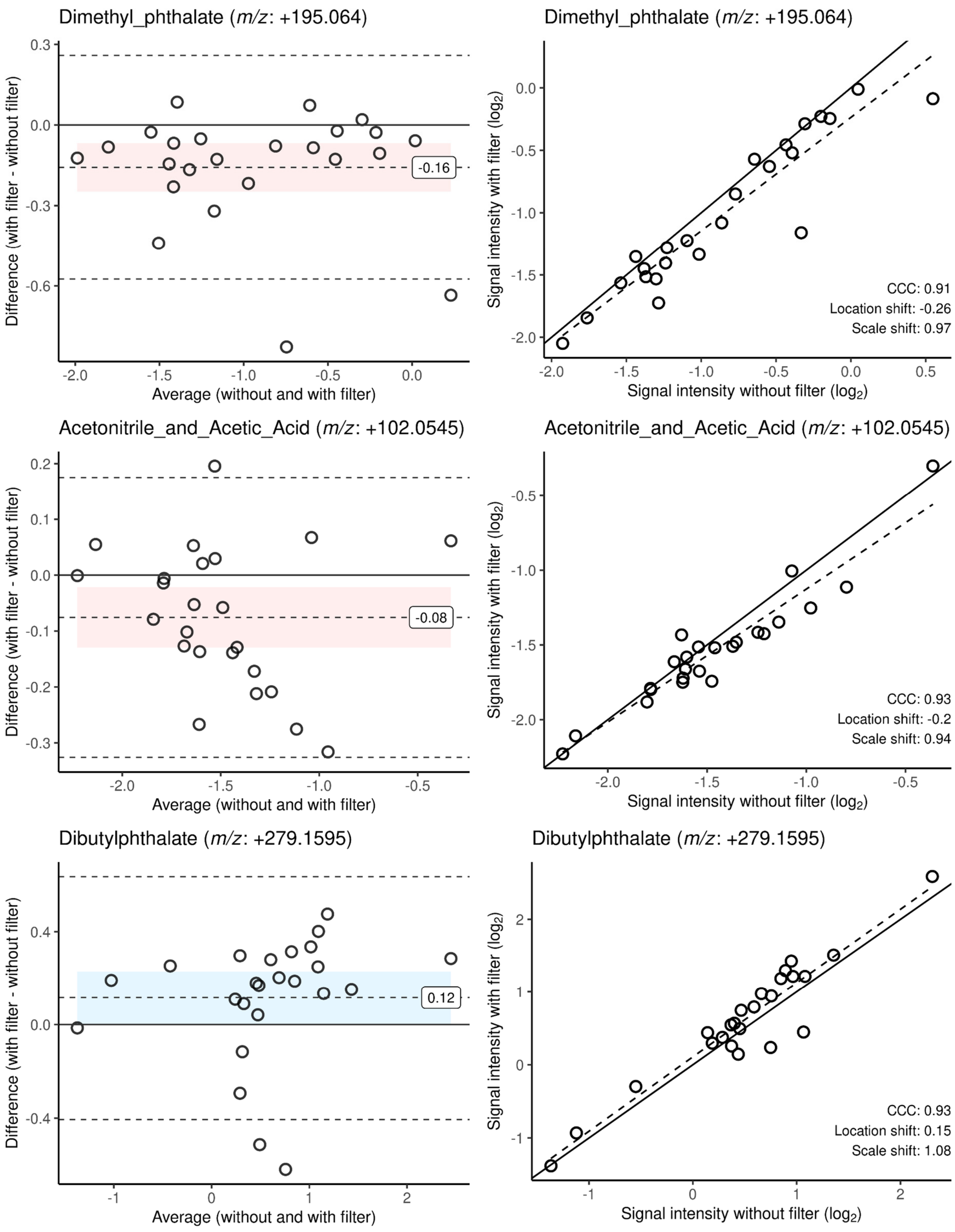

Dimethyl_phthalate (m/z: +195.064)
Difference (with filter - without filter)
Average (without and with filter)
-0.16
Dimethyl_phthalate (m/z: +195.064)
Signal intensity with filter (log2)
Signal intensity without filter (log2)
CCC: 0.91
Location shift: -0.26
Scale shift: 0.97
Acetonitrile_and_Acetic_Acid (m/z: +102.0545)
Difference (with filter - without filter)
Average (without and with filter)
-0.08
Acetonitrile_and_Acetic_Acid (m/z: +102.0545)
Signal intensity with filter (log2)
Signal intensity without filter (log2)
CCC: 0.93
Location shift: -0.2
Scale shift: 0.94
Dibutylphthalate (m/z: +279.1595)
Difference (with filter - without filter)
Average (without and with filter)
0.12
Dibutylphthalate (m/z: +279.1595)
Signal intensity with filter (log2)
Signal intensity without filter (log2)
CCC: 0.93
Location shift: 0.15
Scale shift: 1.08

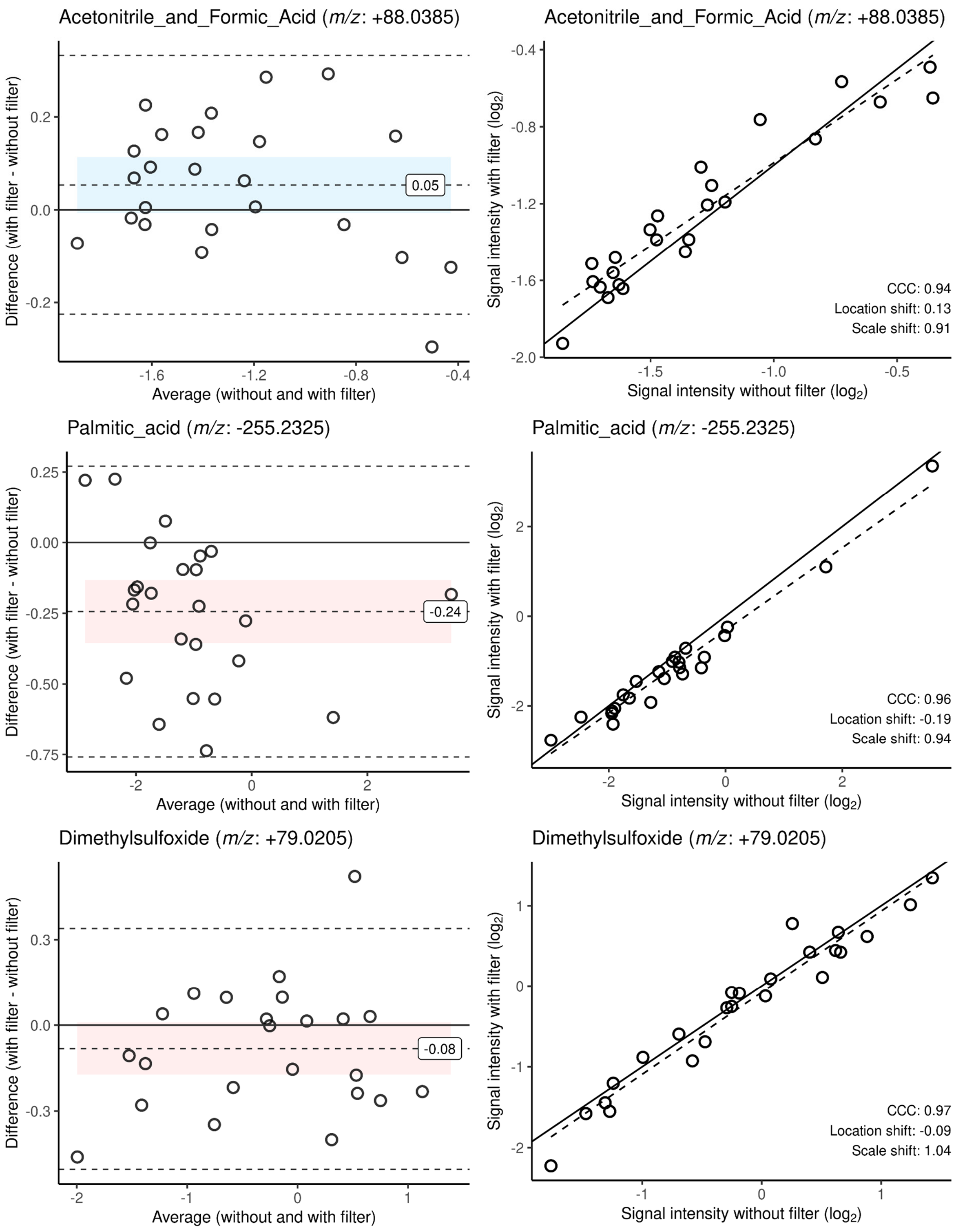
Acetonitrile_and_Formic_Acid (m/z: +88.0385)
Difference (with filter - without filter)
Average (without and with filter)
0.05
Acetonitrile_and_Formic_Acid (m/z: +88.0385)
Signal intensity with filter (log2)
Signal intensity without filter (log2)
CCC: 0.94
Location shift: 0.13
Scale shift: 0.91
Palmitic_acid (m/z: -255.2325)
Difference (with filter - without filter)
Average (without and with filter)
-0.24
Palmitic_acid (m/z: -255.2325)
Signal intensity with filter (log2)
Signal intensity without filter (log2)
CCC: 0.96
Location shift: -0.19
Scale shift: 0.94
Dimethylsulfoxide (m/z: +79.0205)
Difference (with filter - without filter)
Average (without and with filter)
-0.08
Dimethylsulfoxide (m/z: +79.0205)
Signal intensity with filter (log2)
Signal intensity without filter (log2)
CCC: 0.97
Location shift: -0.09
Scale shift: 1.04

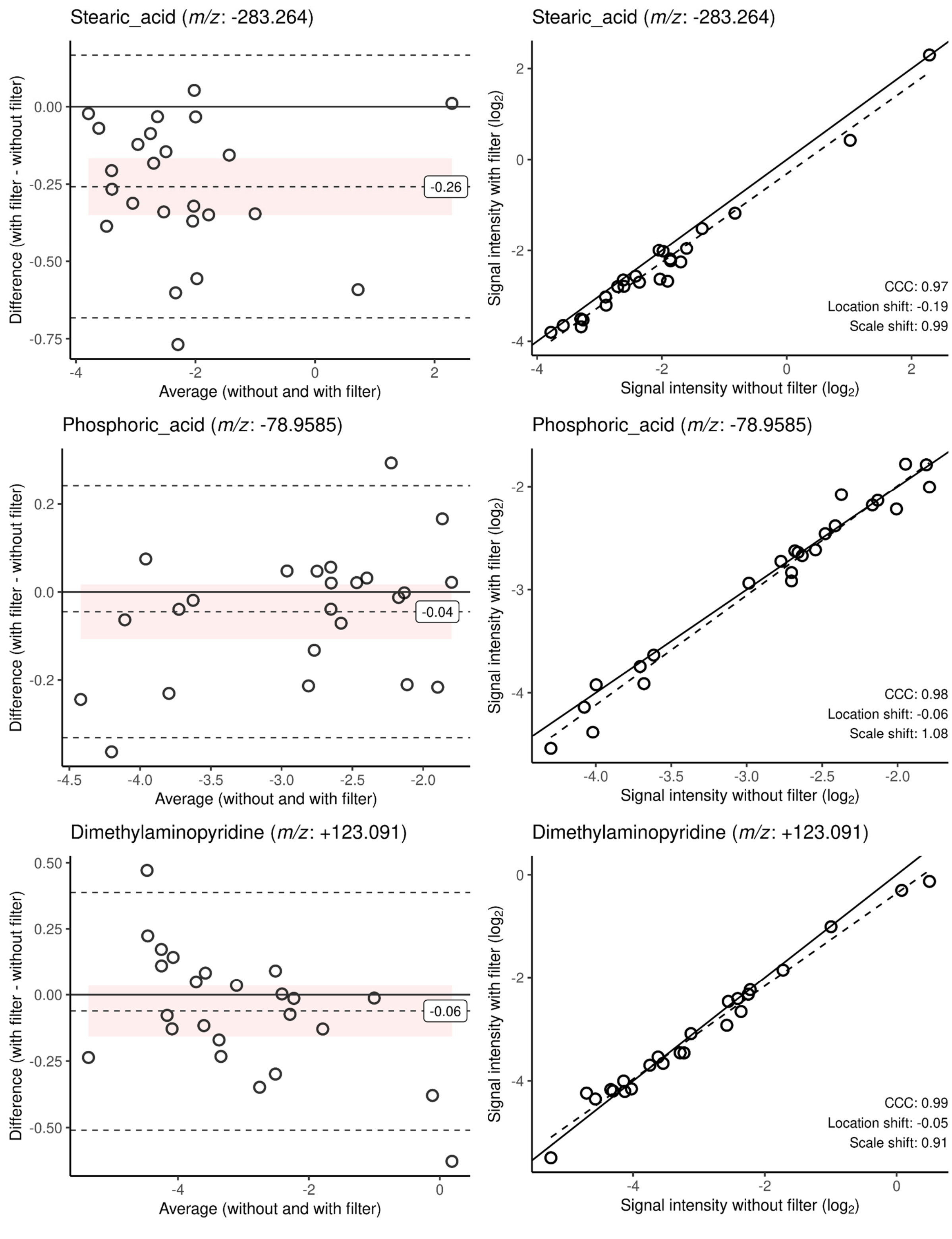

Stearic_acid (*m/z*: -283.264)
Difference (with filter - without filter)
Average (without and with filter)
-0.26
Stearic_acid (*m/z*: -283.264)
Signal intensity with filter (log_2)
Signal intensity without filter (log_2)
CCC: 0.97
Location shift: -0.19
Scale shift: 0.99
Phosphoric_acid (*m/z*: -78.9585)
Difference (with filter - without filter)
Average (without and with filter)
-0.04
Phosphoric_acid (*m/z*: -78.9585)
Signal intensity with filter (log_2)
Signal intensity without filter (log_2)
CCC: 0.98
Location shift: -0.06
Scale shift: 1.08
Dimethylaminopyridine (*m/z*: +123.091)
Difference (with filter - without filter)
Average (without and with filter)
-0.06
Dimethylaminopyridine (*m/z*: +123.091)
Signal intensity with filter (log_2)
Signal intensity without filter (log_2)
CCC: 0.99
Location shift: -0.05
Scale shift: 0.91

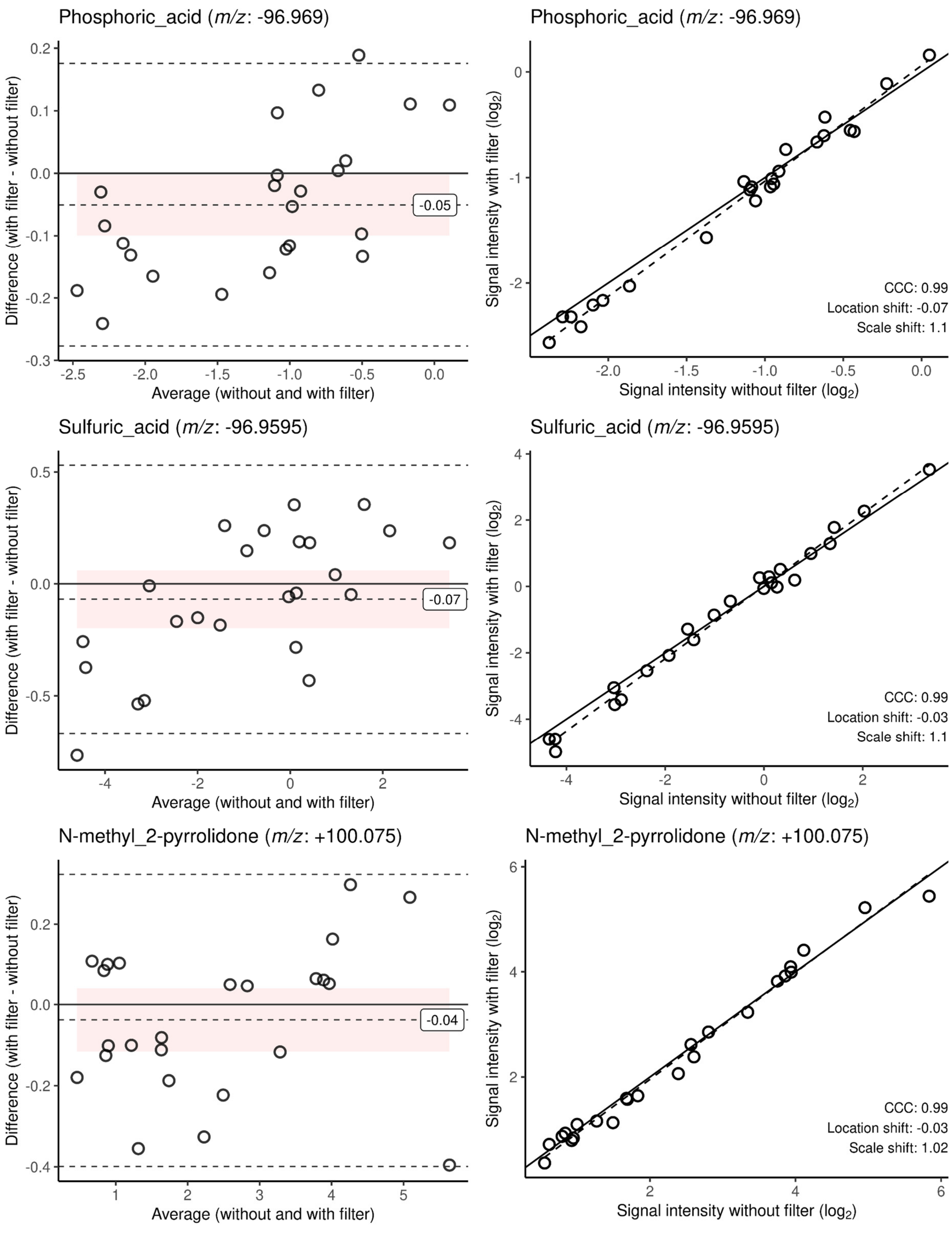

Phosphoric_acid (m/z: -96.969)
Difference (with filter - without filter)
Average (without and with filter)
-0.05
Phosphoric_acid (m/z: -96.969)
Signal intensity with filter (log2)
Signal intensity without filter (log2)
CCC: 0.99
Location shift: -0.07
Scale shift: 1.1
Sulfuric_acid (m/z: -96.9595)
Difference (with filter - without filter)
Average (without and with filter)
-0.07
Sulfuric_acid (m/z: -96.9595)
Signal intensity with filter (log2)
Signal intensity without filter (log2)
CCC: 0.99
Location shift: -0.03
Scale shift: 1.1
N-methyl_2-pyrrolidone (m/z: +100.075)
Difference (with filter - without filter)
Average (without and with filter)
-0.04
N-methyl_2-pyrrolidone (m/z: +100.075)
Signal intensity with filter (log2)
Signal intensity without filter (log2)
CCC: 0.99
Location shift: -0.03
Scale shift: 1.02

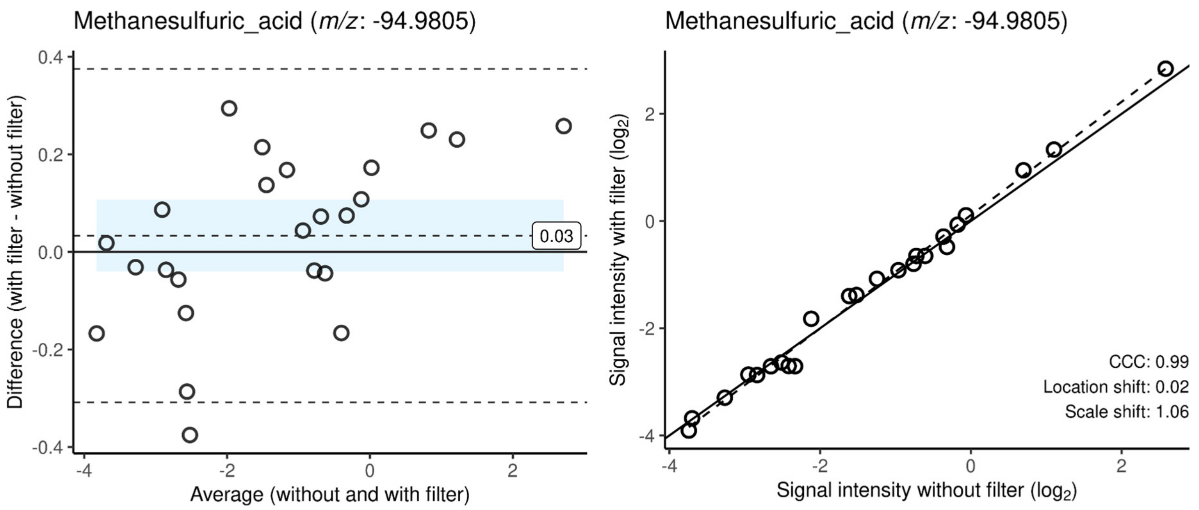

3.3 Figure S3

Plots consisting of contaminants found in ambient air samples measured immediately together with human breath samples (7 plot points). X-axis: within-subject signal intensity differences (without filter - with filter) of the corresponding *m/z* feature. Y-axis: signal intensities in humidified ambient air samples of the corresponding *m/z* feature. The dashed line represents the regression line with a gray area enclosed by the 95% confidence interval limits. Bottom right corner: R^2 and adjusted R^2.

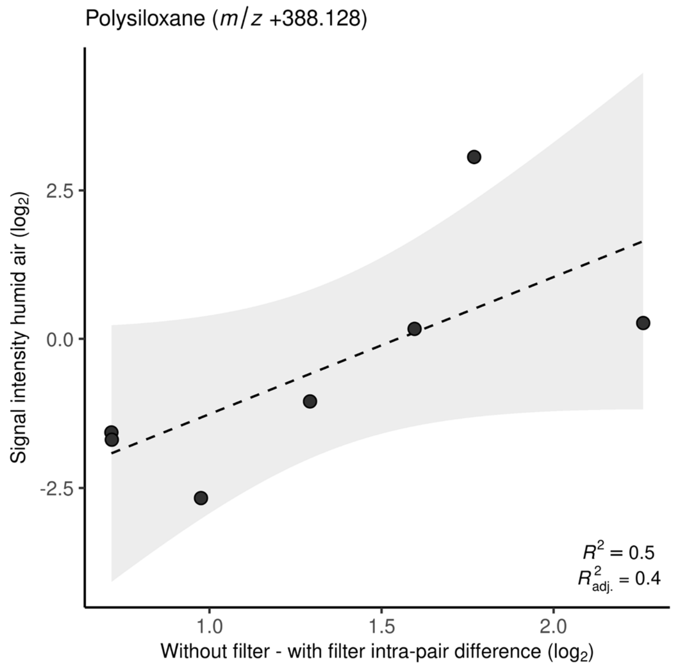

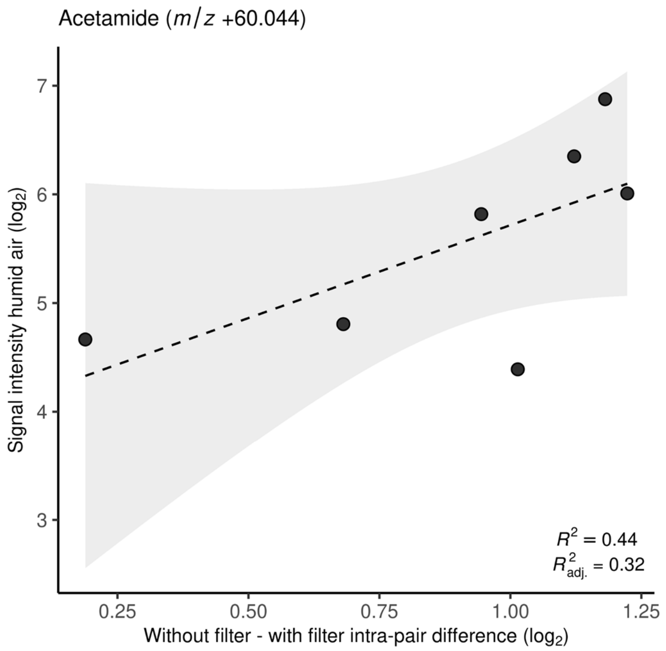

Acetamide (m/z +60.044)
Signal intensity humid air ($\log_2$)
3
4
5
6
7
0.25
0.50
0.75
1.00
1.25
Without filter - with filter intra-pair difference ($\log_2$)
$R^2 = 0.44$
$R^2_{adj.} = 0.32$

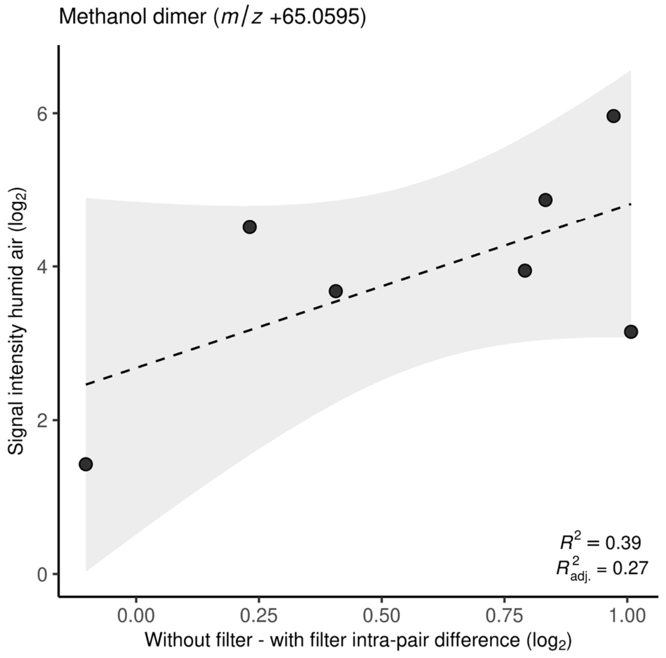

Methanol dimer (m/z +65.0595)
Signal intensity humid air ($\log_2$)
0
2
4
6
0.00
0.25
0.50
0.75
1.00
Without filter - with filter intra-pair difference ($\log_2$)
$R^2 = 0.39$
$R^2_{adj.} = 0.27$

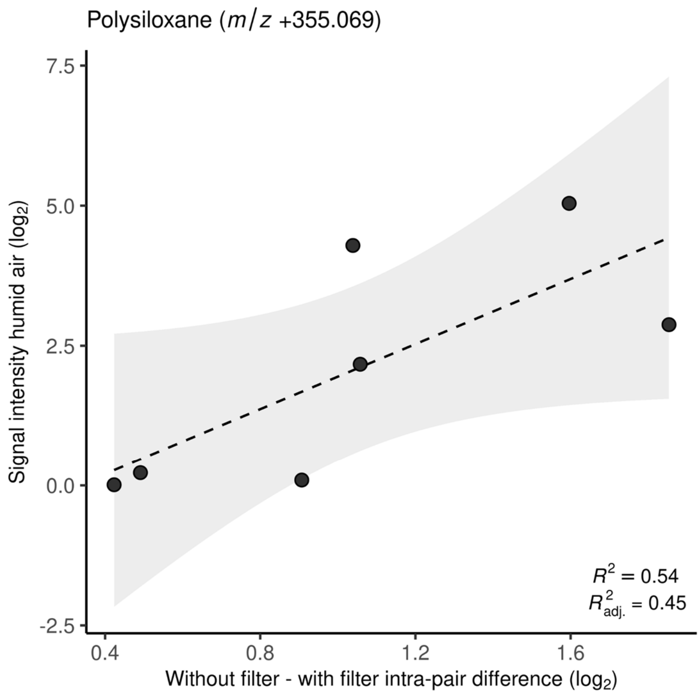

Polysiloxane (m/z +355.069)
Signal intensity humid air (log_2)
7.5
5.0
2.5
0.0
-2.5
R^2 = 0.54
$R^2_{adj.}$ = 0.45
0.4
0.8
1.2
1.6
Without filter - with filter intra-pair difference (log_2)

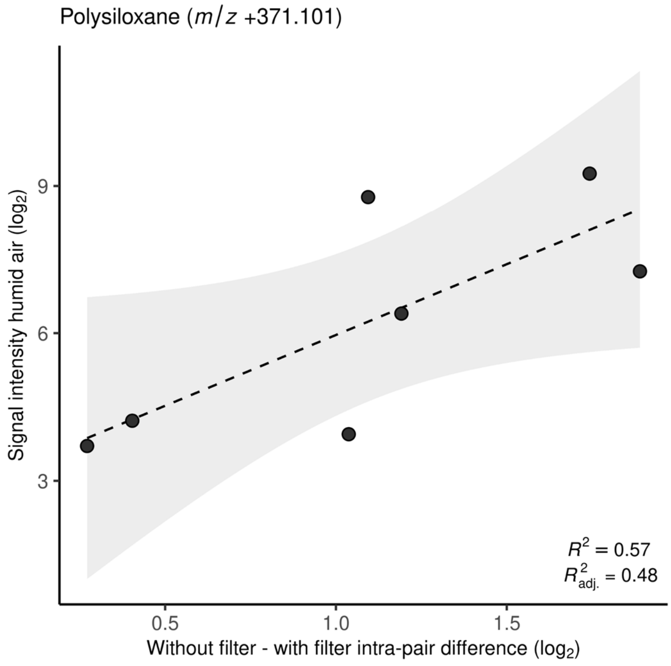

Polysiloxane (m/z +371.101)
Signal intensity humid air (log_2)
9
6
3
R^2 = 0.57
$R^2_{adj.}$ = 0.48
0.5
1.0
1.5
Without filter - with filter intra-pair difference (log_2)

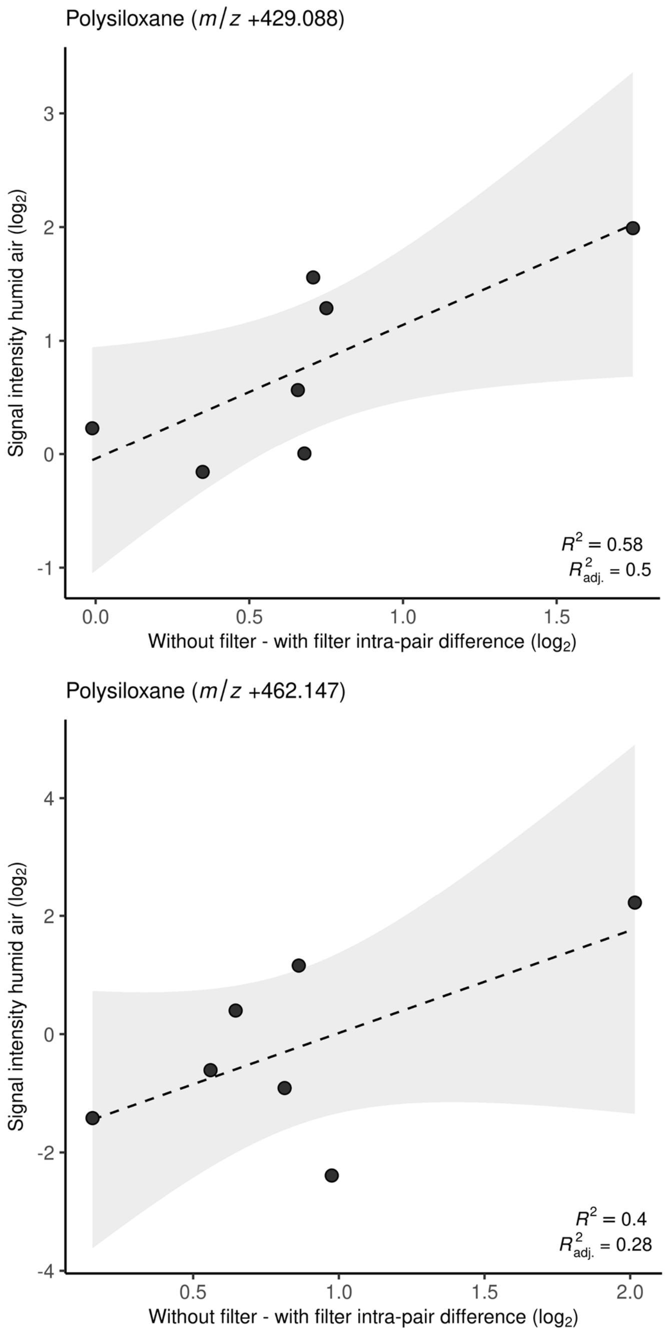

Polysiloxane (m/z +429.088)
Signal intensity humid air (log2)
Without filter - with filter intra-pair difference (log2)
R2 = 0.58
R2adj. = 0.5
3
2
1
0
-1
0.0
0.5
1.0
1.5
Polysiloxane (m/z +462.147)
Signal intensity humid air (log2)
Without filter - with filter intra-pair difference (log2)
R2 = 0.4
R2adj. = 0.28
4
2
0
-2
-4
0.5
1.0
1.5
2.0

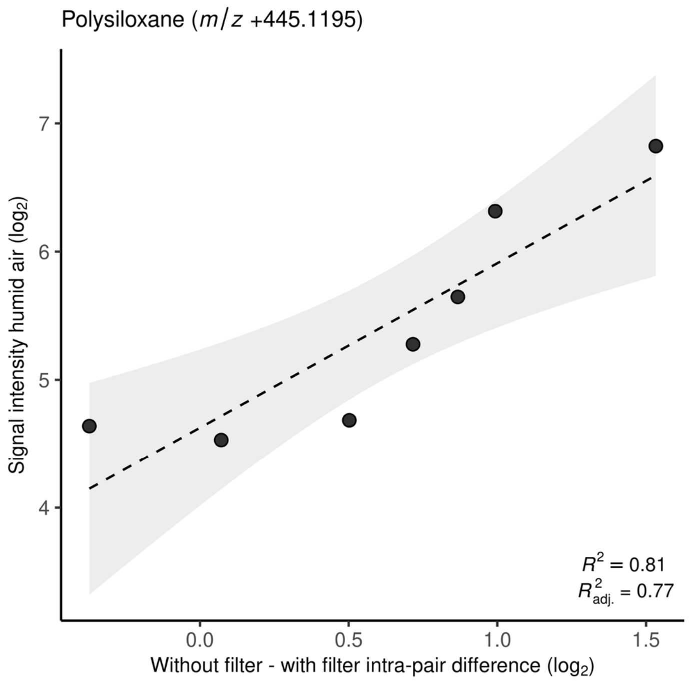

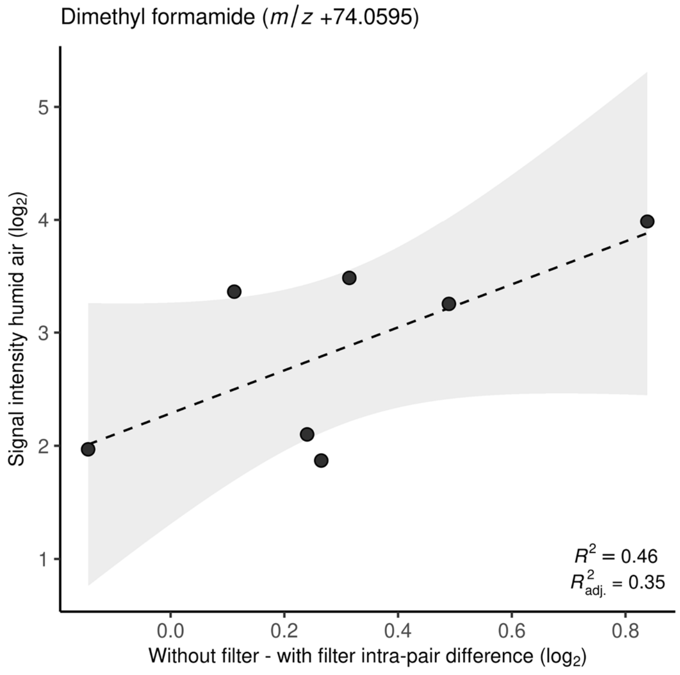

3.4 Figure S4

Plots consisting of metabolites found in ambient air samples measured immediately together with human breath samples (7 plot points). X-axis: within-subject signal intensity differences (without filter - with filter) of the corresponding *m/z* feature. Y-axis: signal intensities in humidified ambient air samples of the corresponding *m/z* feature. The dashed line represents the regression line with a gray area enclosed by the 95% confidence interval limits. Bottom right corner: R^2 and adjusted R^2.

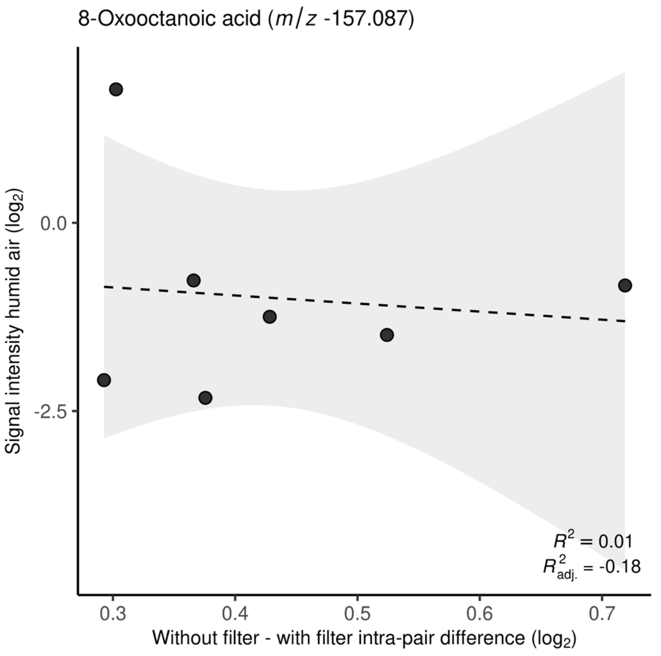
8-Oxooctanoic acid (m/z -157.087)
Signal intensity humid air (log2)
0.0
-2.5
0.3
0.4
0.5
0.6
0.7
Without filter - with filter intra-pair difference (log2)
R^2 = 0.01
$R^2_{adj.}$ = -0.18

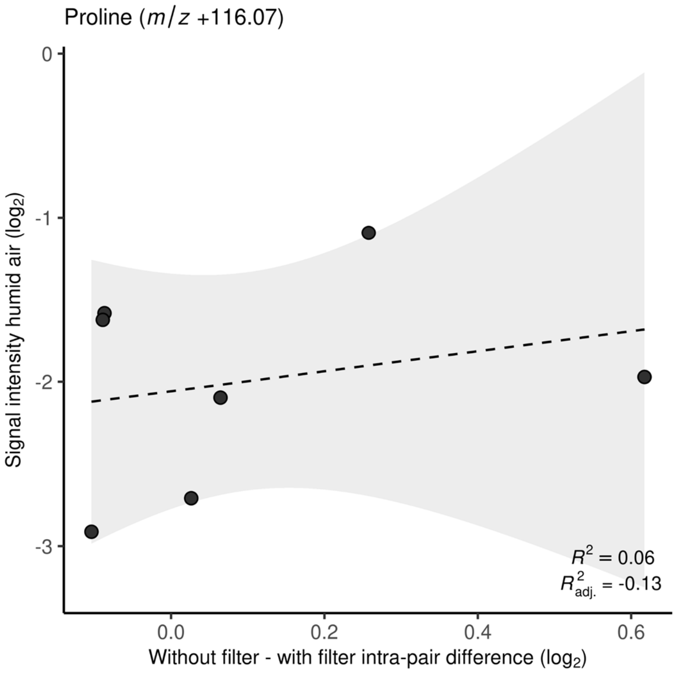
Proline (m/z +116.07)
Signal intensity humid air (log2)
0
-1
-2
-3
0.0
0.2
0.4
0.6
Without filter - with filter intra-pair difference (log2)
R^2 = 0.06
$R^2_{adj.}$ = -0.13

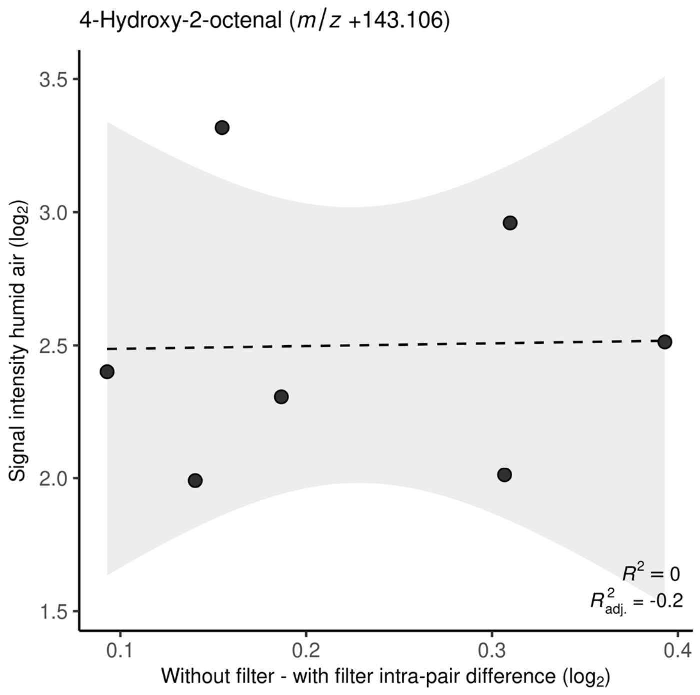
4-Hydroxy-2-octenal (m/z +143.106)
Signal intensity humid air (log2)
3.5
3.0
2.5
2.0
1.5
0.1
0.2
0.3
0.4
R2 = 0
R2adj. = -0.2
Without filter - with filter intra-pair difference (log2)

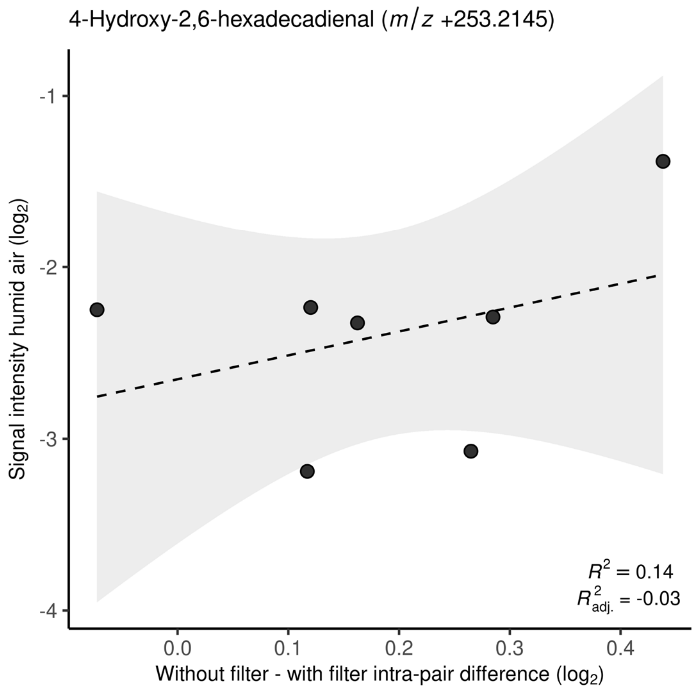
4-Hydroxy-2,6-hexadecadienal (m/z +253.2145)
Signal intensity humid air (log2)
-1
-2
-3
-4
0.0
0.1
0.2
0.3
0.4
R2 = 0.14
R2adj. = -0.03
Without filter - with filter intra-pair difference (log2)

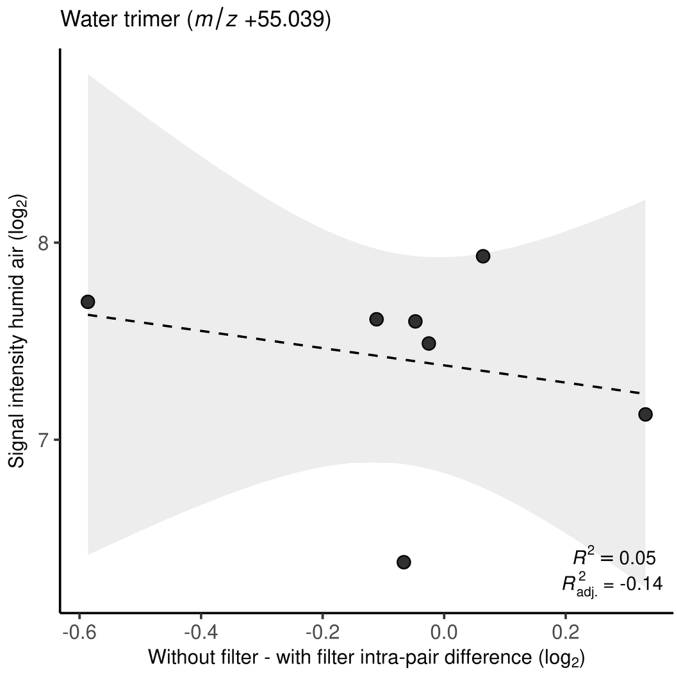
Water trimer (m/z +55.039)
Signal intensity humid air (log_2)
8
7
R^2 = 0.05
$R^2_{adj.}$ = -0.14
-0.6
-0.4
-0.2
0.0
0.2
Without filter - with filter intra-pair difference (log_2)

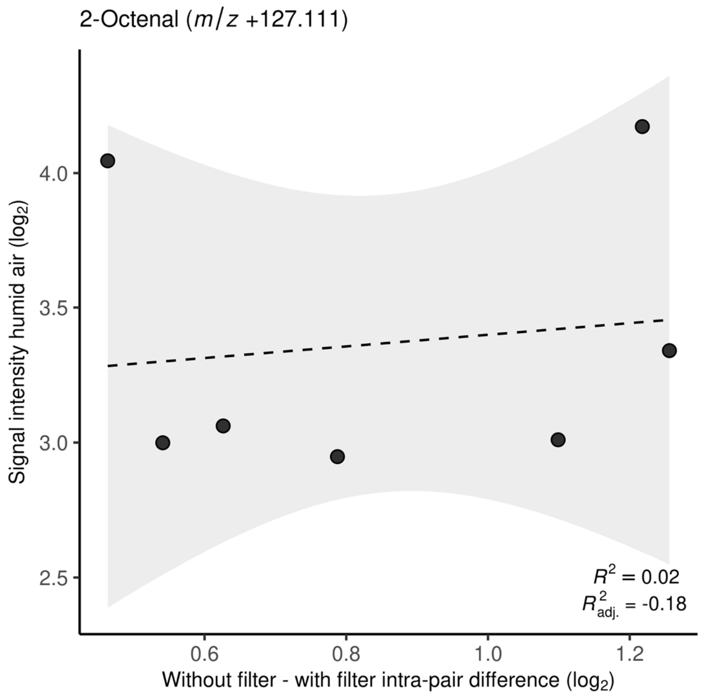
2-Octenal (m/z +127.111)
Signal intensity humid air (log_2)
4.0
3.5
3.0
2.5
R^2 = 0.02
$R^2_{adj.}$ = -0.18
0.6
0.8
1.0
1.2
Without filter - with filter intra-pair difference (log_2)

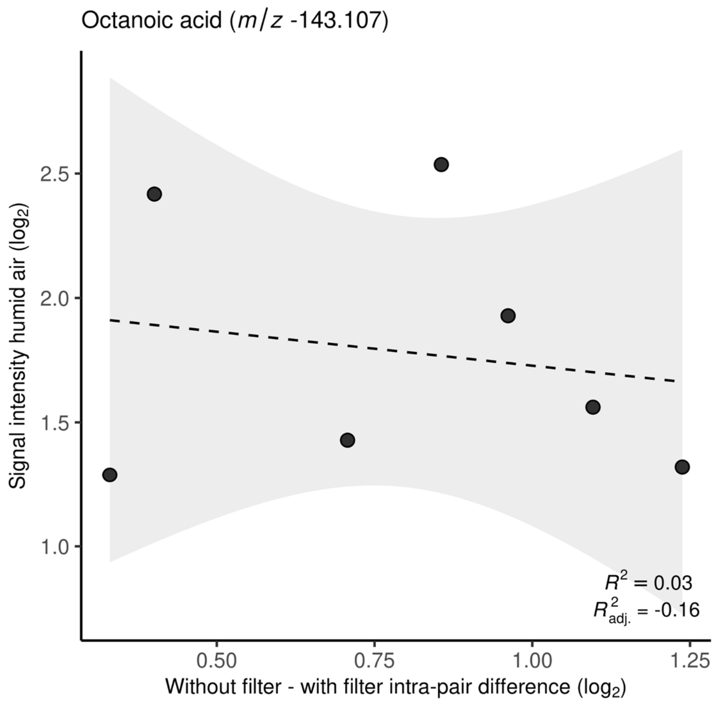
Octanoic acid (m/z -143.107)
Signal intensity humid air (log_2)
2.5
2.0
1.5
1.0
R^2 = 0.03
$R^2_{adj.}$ = -0.16
0.50
0.75
1.00
1.25
Without filter - with filter intra-pair difference (log_2)

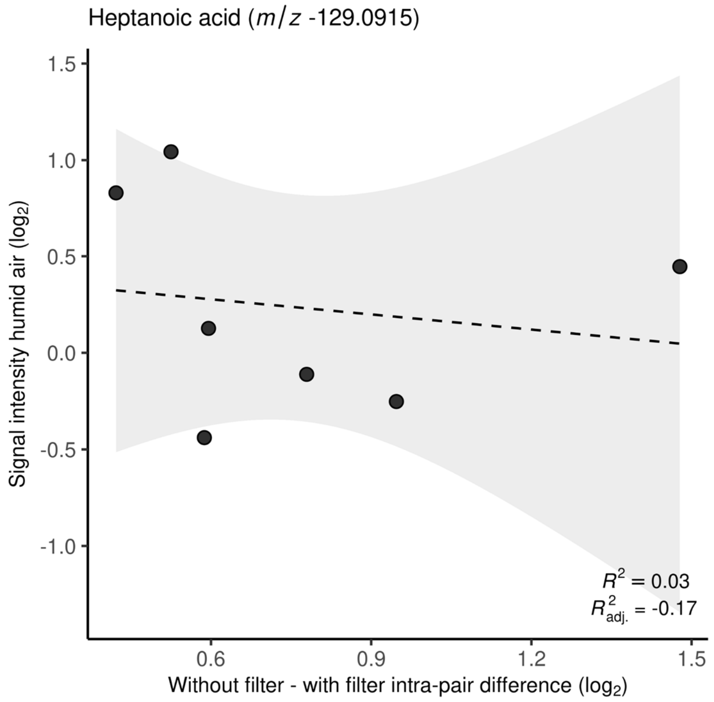
Heptanoic acid (m/z -129.0915)
Signal intensity humid air (log_2)
1.5
1.0
0.5
0.0
-0.5
-1.0
R^2 = 0.03
$R^2_{adj.}$ = -0.17
0.6
0.9
1.2
1.5
Without filter - with filter intra-pair difference (log_2)

References

1. Weber, R.; Haas, N.; Baghdasaryan, A.; Bruderer, T.; Inci, D.; Micic, S.; Perkins, N.; Spinas, R.; Zenobi, R.; Moeller, A. Volatile Organic Compound Breath Signatures of Children with Cystic Fibrosis by Real-Time SESI-HRMS. *ERJ Open Res* **2020**, *6*, 00171–02019.
2. Kaeslin, J.; Micic, S.; Weber, R.; Müller, S.; Perkins, N.; Berger, C.; Zenobi, R.; Bruderer, T.; Moeller, A. Differentiation of Cystic Fibrosis-Related Pathogens by Volatile Organic Compound Analysis with Secondary Electrospray Ionization Mass Spectrometry. *Metabolites* **2021**, *11*, 773.
3. Weber, R.; Streckenbach, B.; Welt, L.; Inci, D.; Kohler, M.; Perkins, N.; Zenobi, R.; Micic, S.; Moeller, A. Metabolic Signatures for Allergic Asthma in Children by On-Line Breath Analysis. *Submitted to the Journal of Allergy and Clinical Immunology* **2022**.
4. Gaugg, M.T.; Engler, A.; Nussbaumer-Ochsner, Y.; Bregy, L.; Stöberl, A.S.; Gaisl, T.; Bruderer, T.; Zenobi, R.; Kohler, M.; Martinez-Lozano Sinues, P. Metabolic Effects of Inhaled Salbutamol Determined by Exhaled Breath Analysis. *J Breath Res* **2017**, *11*, 046004.
5. García-Gómez, D.; Martínez-Lozano Sinues, P.; Barrios-Collado, C.; Vidal-de-Miguel, G.; Gaugg, M.; Zenobi, R. Identification of 2-Alkenals, 4-Hydroxy-2-Alkenals, and 4-Hydroxy-2,6-Alkadienals in Exhaled Breath Condensate by UHPLC-HRMS and in Breath by Real-Time HRMS. *Anal Chem* **2015**, *87*, 3087–3093.
6. Singh, K.D.; Tancev, G.; Decrue, F.; Usemann, J.; Appenzeller, R.; Barreiro, P.; Jaumà, G.; Macia Santiago, M.; Vidal de Miguel, G.; Frey, U.; et al. Standardization Procedures for Real-Time Breath Analysis by Secondary Electrospray Ionization High-Resolution Mass Spectrometry. *Anal Bioanal Chem* **2019**, *411*, 4883–4898.
7. Gaugg, M.T.; Bruderer, T.; Nowak, N.; Eiffert, L.; Martinez-Lozano Sinues, P.; Kohler, M.; Zenobi, R. Mass-Spectrometric Detection of Omega-Oxidation Products of Aliphatic Fatty Acids in Exhaled Breath. *Anal Chem* **2017**, *89*, 10329–10334.
8. García-Gómez, D.; Gaisl, T.; Bregy, L.; Cremonesi, A.; Sinues, P.M.-L.; Kohler, M.; Zenobi, R. Real-Time Quantification of Amino Acids in the Exhalome by Secondary Electrospray Ionization–Mass Spectrometry: A Proof-of-Principle Study. *Clin Chem* **2016**, *62*, 1230–1237.
9. Rioseras, A.T.; Singh, K.D.; Nowak, N.; Gaugg, M.T.; Bruderer, T.; Zenobi, R.; Sinues, P.M.-L. Real-Time Monitoring of Tricarboxylic Acid Metabolites in Exhaled Breath. *Anal Chem* **2018**, *90*, 6453–6460.
10. Martinez-Lozano Sinues, P.; Meier, L.; Berchtold, C.; Ivanov, M.; Sievi, N.; Camen, G.; Kohler, M.; Zenobi, R. Breath Analysis in Real Time by Mass Spectrometry in Chronic Obstructive Pulmonary Disease. *Respiration* **2014**, *87*, 301–310.
11. Nowak, N.; Gaisl, T.; Miladinovic, D.; Marcinkevics, R.; Osswald, M.; Bauer, S.; Buhmann, J.; Zenobi, R.; Sinues, P.; Brown, S.A.; et al. Rapid and Reversible Control of Human Metabolism by Individual Sleep States. *Cell Rep* **2021**, *37*.
12. Martínez-Lozano, P.; Zingaro, L.; Finiguerra, A.; Cristoni, S. Secondary Electrospray Ionization-Mass Spectrometry: Breath Study on a Control Group. *J Breath Res* **2011**, *5*, 016002.
13. Gaugg, M.T.; Nussbaumer-Ochsner, Y.; Bregy, L.; Engler, A.; Stebler, N.; Gaisl, T.; Bruderer, T.; Nowak, N.; Sinues, P.; Zenobi, R.; et al. Real-Time Breath Analysis Reveals Specific Metabolic Signatures of COPD Exacerbations. *Chest* **2019**, *156*, 269–276.
14. Schwarz, E.I.; Martinez-Lozano Sinues, P.; Bregy, L.; Gaisl, T.; Garcia Gomez, D.; Gaugg, M.T.; Suter, Y.; Stebler, N.; Nussbaumer-Ochsner, Y.; Bloch, K.E.; et al. Effects of CPAP Therapy Withdrawal on Exhaled Breath Pattern in Obstructive Sleep Apnoea. *Thorax* **2016**, *71*, 110.
15. García-Gómez, D.; Gaisl, T.; Bregy, L.; Martínez-Lozano Sinues, P.; Kohler, M.; Zenobi, R. Secondary Electrospray Ionization Coupled to High-Resolution Mass Spectrometry Reveals Tryptophan Pathway Metabolites in Exhaled Human Breath. *Chemical Communications* **2016**, *52*, 8526–8528.

16. Keller, B.O.; Sui, J.; Young, A.B.; Whittal, R.M. Interferences and Contaminants Encountered in Modern Mass Spectrometry. *Anal Chim Acta* **2008**, *627*, 71–81.
